# Genetic architecture of quantitative traits in beef cattle revealed by genome wide association studies of imputed whole genome sequence variants: I: feed efficiency and component traits

**DOI:** 10.1186/s12864-019-6362-1

**Published:** 2020-01-13

**Authors:** Feng Zhang, Yining Wang, Robert Mukiibi, Liuhong Chen, Michael Vinsky, Graham Plastow, John Basarab, Paul Stothard, Changxi Li

**Affiliations:** 1 Lacombe Research and Development Centre, Agriculture and Agri-Food Canada, Lacombe, AB Canada; 2 Department of Agricultural, Food and Nutritional Science, University of Alberta, Edmonton, AB Canada; 3 State Key Laboratory for Swine Genetics, Breeding and Production Technology, Jiangxi Agricultural University, Nanchang, Jiangxi China; 4 Present Address: Institute of Translational Medicine, Nanchang University, Nanchang, Jiangxi China; 5 Alberta Agriculture and Forestry, Lacombe Research and Development Centre, 6000 C&E Trail, Lacombe, AB Canada

**Keywords:** Genetic architecture, Imputed whole genome sequence variants, Genome wide association studies, Feed efficiency, Beef cattle

## Abstract

**Background:**

Genome wide association studies (GWAS) on residual feed intake (RFI) and its component traits including daily dry matter intake (DMI), average daily gain (ADG), and metabolic body weight (MWT) were conducted in a population of 7573 animals from multiple beef cattle breeds based on 7,853,211 imputed whole genome sequence variants. The GWAS results were used to elucidate genetic architectures of the feed efficiency related traits in beef cattle.

**Results:**

The DNA variant allele substitution effects approximated a bell-shaped distribution for all the traits while the distribution of additive genetic variances explained by single DNA variants followed a scaled inverse chi-squared distribution to a greater extent. With a threshold of *P*-value < 1.00E-05, 16, 72, 88, and 116 lead DNA variants on multiple chromosomes were significantly associated with RFI, DMI, ADG, and MWT, respectively. In addition, lead DNA variants with potentially large pleiotropic effects on DMI, ADG, and MWT were found on chromosomes 6, 14 and 20. On average, missense, 3’UTR, 5’UTR, and other regulatory region variants exhibited larger allele substitution effects in comparison to other functional classes. Intergenic and intron variants captured smaller proportions of additive genetic variance per DNA variant. Instead 3’UTR and synonymous variants explained a greater amount of genetic variance per DNA variant for all the traits examined while missense, 5’UTR and other regulatory region variants accounted for relatively more additive genetic variance per sequence variant for RFI and ADG, respectively. In total, 25 to 27 enriched cellular and molecular functions were identified with lipid metabolism and carbohydrate metabolism being the most significant for the feed efficiency traits.

**Conclusions:**

RFI is controlled by many DNA variants with relatively small effects whereas DMI, ADG, and MWT are influenced by a few DNA variants with large effects and many DNA variants with small effects. Nucleotide polymorphisms in regulatory region and synonymous functional classes play a more important role per sequence variant in determining variation of the feed efficiency traits. The genetic architecture as revealed by the GWAS of the imputed 7,853,211 DNA variants will improve our understanding on the genetic control of feed efficiency traits in beef cattle.

## Background

Improving animal meat production efficiency has become an imperative goal for the industry to achieve as the global demand for meat products continues to increase due to population growth and improved economic prosperity in the developed and developing countries. Animal meat production efficiency is primarily determined by an animal’s ability to convert consumed feed into saleable meat as feeding related cost is the single largest variable expense in animal production [1–3]. Of meat production animals, beef cattle are the largest and the feed provision accounts for up to 70% of total production costs [4]. In addition, studies have shown that more efficient beef cattle not only consume less feed for the same amount of meat produced but also have less methane emission [5–7]. Therefore, improving feed efficiency will increase profitability, reduce environmental footprints, and thus lead to a more sustainable beef production industry.

Feed efficiency can be measured in different ways [8–12], of which residual feed intake has gained popularity as it is phenotypically independent of growth and body size [12]. Residual feed intake (RFI) is usually defined as the difference between the actual daily dry matter intake (DMI) of an animal and the expected daily DMI required for average daily gain (ADG) and metabolic body weight (MWT) [11]. RFI has shown considerable variations among animals with a moderate heritability estimate [13, 14], which allows a reasonable response to genetic/genomic selection for more efficient beef cattle. Furthermore, feed efficiency traits are relatively difficult and expensive to measure, which makes them good candidates for genomic selection. However, genomic prediction accuracy of feed efficiency traits in beef cattle has been relatively low [15–17], largely due to limited numbers of animals in the reference population and/or a lack of information on causative DNA variants on the trait. Therefore, identification of DNA variants responsible for variation in feed efficiency traits of beef cattle will help design a better genomic prediction strategy to improve genomic selection accuracy.

Feed efficiency is a complex trait and it is likely controlled by multiple genes involved in several physical, physiological and metabolic processes such as feed intake, digestion, body composition, tissue metabolism, activity and thermoregulation [18–20]. Research has been conducted to identify chromosomal regions or gene polymorphisms that are associated with the trait through linkage and association studies, and a Cattle QTL database including RFI is available [21]. The detection of these QTLs has improved our understanding on the genetic control of different quantitative traits. However, the genetic mechanism of feed efficiency traits still remains largely unknown as previous studies used a relatively low density of DNA markers, which limited the power to identify causative mutations. Although sequencing whole genome DNA variants represents an ideal way to genotype animals for genome wide association studies (GWAS), full sequencing a large cohort of animals is not feasible at this stage due to its prohibitive costs. Therefore, an alternative way is to impute genotypes of individuals from low density DNA markers to whole genome sequence (WGS) variants. The improved power of GWAS based on imputed WGS variants was reported in studies on milk protein composition in dairy cattle [22], lumbar number in Sutai pigs [23], fertility and calving traits in Brown Swiss cattle [24], and milk fat percentage in Fleckvieh and Holstein cattle [25]. In this study, we imputed 50 K SNP genotypes to whole genome sequence variants and investigated the effect for each of imputed 7,853,211 DNA variants (SNPs and INDELs) based on a sample of 7573 Canadian beef cattle, with an aim to elucidate genetic architectures of RFI and its component traits DMI, ADG, and MWT.

## Results

### Descriptive statistics and genomic heritability estimation

The descriptive statistics of four feed efficiency related traits including mean, standard deviation, additive genetic variances (±SE), and heritability estimates (±SE) obtained based on the 50 K SNP and 7,853,211 DNA variant (or 7.8 M sequence variant) panels were shown in Table 1. The means and standard deviations were calculated based on raw phenotypic values (i.e. unadjusted phenotypic values), and they were consistent with those previously reported by Lu et al. [17], Mao et al. [13], and Zhang et al. [26]. The heritability estimates for RFI based on the imputed 7.8 M sequence variants (0.26 ± 0.02) and the 50 K SNP panel (0.22 ± 0.02) were comparable to those reported by Nkrumah et al. [14] (0.21 ± 0.12) and Zhang et al. [26] (0.23 ± 0.06) in Canadian crossbred beef cattle but tended to be in the lower range of RFI heritability values reported from other research [13, 27–31]. The heritability estimates of DMI and ADG with the 50 K SNPs (0.32 ± 0.02 and 0.21 ± 0.02, respectively) and the 7.8 M sequence variant panel (0.39 ± 0.02 and 0.26 ± 0.02, respectively) were similar to those reported by Arthur et al. in Charolais [28] (0.34 ± 0.07 and 0.20 ± 0.06) and in Angus [27] (0.39 ± 0.03 and 0.28 ± 0.04), but lower than those reported by other studies (0.39 ± 0.10 to 0.54 ± 0.13 and 0.30 ± 0.06 to 0.59 ± 0.17 in [13, 14, 29]), and greater than the estimates in [26, 30] (ranging from 0.18 ± 0.10 to 0.27 ± 0.15) and 0.09 ± 0.04 to 0.11 ± 0.04 reported by Zhang et al. [26]). The heritability estimates of MWT obtained based on the 50 K SNPs (0.44 ± 0.02) and the 7.8 M sequence variants (0.53 ± 0.02) were greater than most other reports [13, 14, 26, 27, 31]. Notably, the amounts of additive genetic variance obtained by the imputed 7.8 M sequence variant panel and subsequently the heritability estimates were 18.2% for RFI to 23.8% for ADG greater than that obtained using the 50 K SNP panel for all traits (Table 1), indicating that the imputed 7.8 M sequence variant panel captures more additive genetic variance for the traits in comparison to the 50 K SNP panel.

**Table 1:** Descriptive statistics of phenotypic data, additive genetic variances and heritability estimates based on the 50 K SNP and the imputed 7.8 M whole genome sequence (WGS) variants in a beef cattle multibreed population (*N* = 7573) for RFI and its component traits

Traits^a^	mean (SD)	σ_a_^2^ ± SE_50K	h^2^ ± SE_50K	σ_a_^2^ ± SE_7.8 M	h^2^ ± SE_7.8 M
RFI	0 (0.68)	0.10 ± 0.01	0.22 ± 0.02	0.12 ± 0.01	0.26 ± 0.02
DMI	9.27 (1.61)	0.30 ± 0.02	0.32 ± 0.02	0.36 ± 0.02	0.39 ± 0.02
ADG	1.44 (0.4)	0.011 ± 0.001	0.21 ± 0.02	0.014 ± 0.001	0.26 ± 0.02
MWT	93.69 (13.23)	17.85 ± 0.70	0.44 ± 0.02	21.34 ± 1.01	0.53 ± 0.02

^a^RFI residual feed intake in kg of DMI per day, *DMI* daily dry matter intake in kg per day, *ADG* average daily gain in kg, *MWT* metabolic body weight in kg. *Mean (SD)* mean of raw phenotypic values and standard deviation (SD), *σ*_*a*_^*2*^ *± SE* additive genetic variance ± standard error (SE), *h*^*2*^ *± SE* heritability estimate ± SE

### Comparison of GWAS results between 7.8 M and 50 K SNP panels

A summary of numbers of significant SNPs at the suggestive *P*-value < 0.005, significant *P*-value < 1.00E-05 and FDR < 0.10, and numbers of corresponding lead SNPs (or DNA variants) were presented in Table 2 for the 7.8 M DNA variant panel. The GWAS results were compared between the 7.8 M sequence variant panel and 50 K SNP panel. It was found that the majority of significant SNPs at the suggestive significance threshold *P*-value < 0.005 detected by the 50 K SNP panel for RFI, DMI, ADG, and MWT were also identified by the 7.8 M sequence variant panel with a *P*-value < 0.005. The rest of the suggestive SNPs (12 or 0.1% for RFI to 39 or 0.2% for MWT) were detected by the 7.8 M sequence variant panel with a relaxed significance threshold of *P*-value < 2 × 0.005 = 0.01. Since all SNPs in the 50 K SNP panel were included in the 7.8 M sequence variant panel, it is expected that the SNP allele substitution effects and their significance test of *P*-value would be the same for both GWAS analyses if the same G matrix was used. The slight difference of *P*-values observed in this study is likely due to the different G matrix used in the 7.8 M sequence variant and 50 K SNP GWAS analyses. However, it is clearly shown that the 7.8 M sequence variant panel detected additional or novel significant SNPs at various significant thresholds for all the traits than the 50 K SNP panel as summarized in Table 2, indicating that the 7.8 M sequence variant panel improved the power of GWAS to detect associations for the traits. Therefore, we will focus on the GWAS results of the 7.8 M sequence variant panel in the subsequent result sections. For simplicity, we will refer all the 7.8 M sequence variants (SNPs and INDELs) as SNPs in some cases.

**Table 2:** A summary of number of significant SNPs detected by the 7.8 M WGS variant GWAS for RFI and its component traits in a beef cattle multibreed population

Trait^a^	RFI	DMI	ADG	MWT
Suggestive (*p* < 0.005)	41,248 (31,385)	46,455 (32,230)	44,746 (30,447)	47,923 (31,012)
Lead Suggestive	4048 (3729)	4104 (3772)	3881 (3547)	4143 (3764)
Significant (*p* < 1.00E-05)	54 (35)	2024 (431)	2584 (759)	4011 (935)
Lead Significant	16 (12)	72 (35)	88 (45)	116 (56)
FDR (FDR < 0.10)	0 (0)	2727 (431)	3952 (759)	5897 (935)
Lead FDR	0 (0)	72 (35)	88 (45)	116 (56)

^a^*RFI* residual feed intake in kg of DMI per day, *DMI* daily dry matter intake in kg per day, *ADG* average daily gain in kg, *MWT* metabolic body weight in kg. *FDR* genome-wise false discovery rate (FDR) calculated followed the Benjamini-Hochberg procedure [32]. The numbers of additional or novel significant SNPs in comparison to the 50 K SNP panel were presented in the parentheses

### Distributions of SNP effects

Distribution of SNP allele substitution effects were obtained with all 7,853,211 DNA variants, which showed a clear bell-shaped distribution for all the traits (Additional file 1: Figure S1), with the majority of the variants having zero or near zero effects on all traits. Of all the 7,853,211 SNP allele substitution effects, only a very small proportion reached a suggestive *P*-value < 0.005, ranging from 0.53% for RFI to 0.61% for MWT (Table 2). The distributions of additive genetic variances explained by individual sequence variants were more like a scaled inverse chi-squared distribution (Additional file 1: Figure S1).

### Average SNP effects and additive genetic variance estimates related to functional classes

To quantify the relative importance of functional SNP classes on the traits, the average of squared SNP allele substitution effects and the additive genetic variance captured by the DNA variants in each functional class were presented in Table 3. In terms of the average of squared SNP allele substitution effects for a functional class (i.e. class mean effect), missense variants, 3’UTR variants, 5’UTR variants, and other regulatory variants were among the top important functional classes as measured by the ratio of their class mean effect to the weighted average of squared SNP allele substitution effects of all functional classes, whereas synonymous variants, intron variants, and intergenic region variants were among the least important functional classes (Table 3). For the additive genetic variance, it was observed that intergenic region and intron variants captured relatively more total additive genetic variance than other functional classes for all the traits. However, their amounts of additive genetic variance explained per DNA variant were smaller for all the traits investigated (Table 3). Instead, 3’UTR and synonymous variants accounted for a greater amount of additive genetic variance per DNA variant for all the traits examined (Table 3). In addition, missense variants and 5’UTR variants explained relatively more additive genetic variance per sequence variant for RFI while other regulatory variants had more additive genetic variance captured per DNA variant for ADG.

**Table 3:** A summary of SNP allele substitution effect and additive genetic variance for each functional class based on imputed 7.8 M variant GWAS for RFI and its component traits in a beef cattle multibreed population

Trait^1^	Class^2^	no_of_SNP^3^	class_mean^4^	Ratio^5^	Vgf ± SE^6^	Vgo ± SE^7^	Vg_total ± SE^8^	Vgf/SNP^9^	Vgf_Ratio^10^
RFI	Intergenic region variants	5,251,680	0.000461	0.997835	0.067 ± 0.015	0.048 ± 0.014	0.12 ± 0.01	0.001283	0.05765900
	Downstream gene variants	253,163	0.000478	1.034632	0.01 ± 0.012	0.105 ± 0.015	0.12 ± 0.01	0.004142	0.18608265
	Upstream gene variants	285,798	0.000480	1.038961	0.002 ± 0.011	0.114 ± 0.015	0.12 ± 0.01	0.000644	0.02894225
	Synonymous variants	32,019	0.000454	0.982684	0.01 ± 0.01	0.106 ± 0.014	0.12 ± 0.01	0.031869	1.43185934
	Intron variants	1,987,366	0.000461	0.997835	0.039 ± 0.014	0.077 ± 0.015	0.12 ± 0.01	0.001966	0.08835385
	Missense variants	17,654	0.000522	1.129870	0.006 ± 0.008	0.11 ± 0.013	0.12 ± 0.01	0.036643	1.64638613
	3′ UTR variants	15,851	0.000490	1.060606	0.011 ± 0.007	0.105 ± 0.012	0.12 ± 0.01	0.070273	3.15738258
	5′ UTR variants	3309	0.000515	1.114719	0.002 ± 0.005	0.114 ± 0.011	0.12 ± 0.01	0.053490	2.40333421
	Other regulatory regions	6371	0.000501	1.084416	0 ± 0.007	0.119 ± 0.012	0.12 ± 0.01	0.000000	0.0000000
DMI	Intergenic region variants	5,251,680	0.000946	0.998944	0.219 ± 0.032	0.141 ± 0.03	0.36 ± 0.03	0.004173	0.15156143
	Downstream gene variants	253,163	0.000970	1.024287	0.011 ± 0.025	0.348 ± 0.033	0.36 ± 0.03	0.004527	0.16439637
	Upstream gene variants	285,798	0.000967	1.021119	0.00001 ± 0.024	0.362 ± 0.033	0.36 ± 0.03	0.000000	0.00001300
	Synonymous variants	32,019	0.000924	0.975713	0.009 ± 0.021	0.35 ± 0.031	0.36 ± 0.03	0.029379	1.06696756
	Intron variants	1,987,366	0.000944	0.996832	0.119 ± 0.029	0.241 ± 0.032	0.36 ± 0.03	0.005984	0.21733452
	Missense variants	17,654	0.001038	1.096093	0.00001 ± 0.02	0.362 ± 0.029	0.36 ± 0.02	0.000006	0.00020571
	3′ UTR variants	15,851	0.001009	1.065470	0.032 ± 0.016	0.327 ± 0.027	0.36 ± 0.02	0.203703	7.39785415
	5' UTR variants	3309	0.000978	1.032735	0.00001 ± 0.011	0.365 ± 0.026	0.37 ± 0.02	0.000030	0.00109752
	Other regulatory regions	6371	0.001017	1.073918	0.00001 ± 0.015	0.362 ± 0.028	0.36 ± 0.02	0.000016	0.00057003
ADG	Intergenic region variants	5,251,680	0.000052	1.000000	0.009 ± 0.002	0.004 ± 0.002	0.014 ± 0.002	0.000178	0.05631654
	Downstream gene variants	253,163	0.000054	1.038462	0.0004 ± 0.001	0.013 ± 0.002	0.014 ± 0.002	0.000143	0.04529727
	Upstream gene variants	285,798	0.000054	1.038462	0 ± 0.001	0.014 ± 0.002	0.014 ± 0.001	0.000000	0.00000000
	Synonymous variants	32,019	0.000051	0.980769	0.001 ± 0.001	0.013 ± 0.002	0.014 ± 0.001	0.003891	1.22935097
	Intron variants	1,987,366	0.000051	0.980769	0.003 ± 0.002	0.01 ± 0.002	0.014 ± 0.002	0.000176	0.05555651
	Missense variants	17,654	0.000058	1.115385	0 ± 0.001	0.014 ± 0.001	0.014 ± 0.001	0.000000	0.00000000
	3′ UTR variants	15,851	0.000054	1.038462	0.001 ± 0.001	0.013 ± 0.001	0.014 ± 0.001	0.005924	1.87143409
	5' UTR variants	3309	0.000055	1.057692	0 ± 0.001	0.014 ± 0.001	0.014 ± 0.001	0.000000	0.00000000
	Other regulatory regions	6371	0.000060	1.153846	0.001 ± 0.001	0.013 ± 0.001	0.014 ± 0.001	0.018176	5.74204463
MWT	Intergenic region variants	5,251,680	0.040609	0.998795	13.14 ± 1.47	7.93 ± 1.38	21.07 ± 1.42	0.250139	0.43808451
	Downstream gene variants	253,163	0.041833	1.028900	0.9 ± 1.1	20.14 ± 1.53	21.04 ± 1.34	0.354482	0.62082809
	Upstream gene variants	285,798	0.041653	1.024472	0.76 ± 1.09	20.27 ± 1.53	21.03 ± 1.33	0.265853	0.46560607
	Synonymous variants	32,019	0.040382	0.993212	0.71 ± 0.97	20.34 ± 1.45	21.05 ± 1.24	2.216215	3.88140336
	Intron variants	1,987,366	0.040446	0.994786	6.3 ± 1.32	14.77 ± 1.48	21.07 ± 1.4	0.317024	0.55522447
	Missense variants	17,654	0.044912	1.104629	0.00004 ± 0.75	21.14 ± 1.33	21.14 ± 1.08	0.000227	0.00039682
	3′ UTR variants	15,851	0.041232	1.014118	0.27 ± 0.63	20.75 ± 1.27	21.03 ± 1.01	1.733070	3.03524000
	5' UTR variants	3309	0.041624	1.023759	0.00004 ± 0.45	21.29 ± 1.19	21.29 ± 0.91	0.001209	0.00211709
	Other regulatory regions	6371	0.043722	1.075360	0.00004 ± 0.65	21.05 ± 1.28	21.05 ± 1.02	0.000628	0.00109959

^*1*^*RFI* residual feed intake in kg of DMI per day, *DMI* daily dry matter intake in kg per day, *ADG* average daily gain in kg, *MWT* metabolic body weight in kg

^2^Other regulatory regions consisted of splice regions in intron variants, disruptive in-frame deletion, splice region variants, etc. Detail functional class assignments of DNA variants can be found in (Additional file 3: Table S2)

^3^Number of DNA variants (or SNPs in text for simplicity)

^4^class_mean is the average of squared SNP allele substitution effects (class_mean) for the functional class

^5^Ratio is ratio of the class_mean of the functional class over the weighted average of class_means of all functional classes

^6^V_gf_ ± SE is additive genetic variance of the functional class ± standard error (SE)

^7^V_go_ ± SE is additive genetic variance of the rest of SNPs in other functional classes ± standard error (SE)

^8^Vg_total ± SE is total additive genetic variance of all 7.8 M WGS variants ± standard error (SE)

^9^V_gf_/SNP is additive genetic variance of the functional class per SNP × 10^5^

^10^Vgf_Ratio is ratio of additive genetic variance of the functional class per SNP over the average of additive genetic variance per SNP of all functional classes based on the imputed 7.8 M WGS variant GWAS

### Top significant SNPs associated with RFI and its component traits

**Fig. 1:**
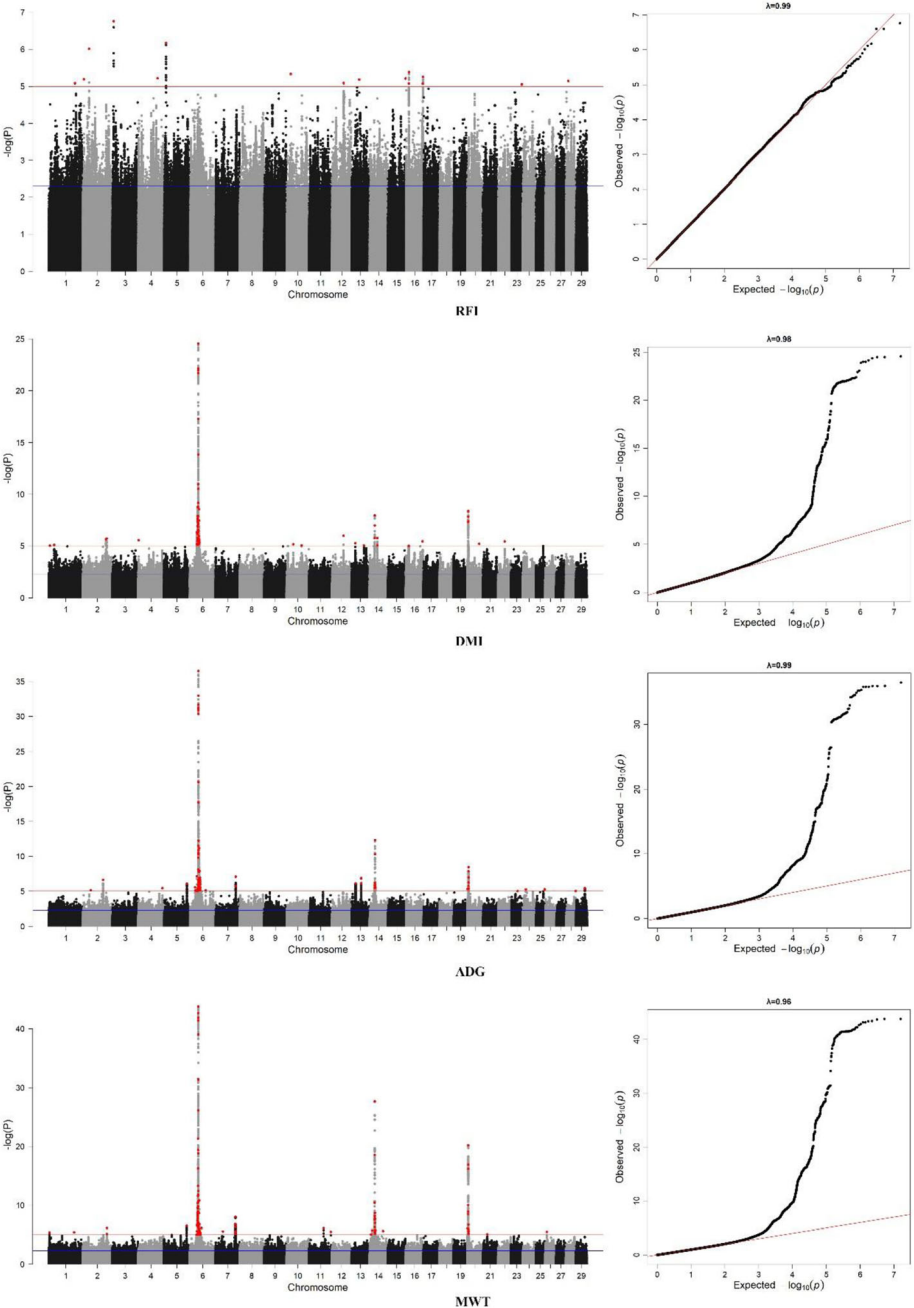
Manhattan (left) and Q-Q (right) plots of GWAS results based on the imputed 7.8 M DNA variant panel for residual feed intake (RFI) and its component traits daily dry matter intake (DMI), average daily gain (ADG), and metabolic body weight (MWT). The blue line indicates a threshold of *P*-value < 0.005 while the red line shows the threshold of *P*-value < 1.00E-05. The red dot is lead SNPs with the threshold of *P*-value < 1.00E-05

Manhattan plots of GWAS results based on the imputed 7.8 M sequence variant panel for RFI and its component traits were presented in Fig. 1. At the suggestive significant level of *P*-value < 0.005, 41,248, 46,455, 44,746, and 47,923 SNPs (i.e. sequence variants) were found to be associated with RFI, DMI, ADG, and MWT, respectively (Table 2). Information on all suggestive significant SNPs was presented in the supplementary excel file of Additional file 2. These SNPs were represented by 4048, 4104, 3881, and 4143 lead suggestive SNPs, respectively, and they were distributed on all the autosomes. When a *P*-value < 1.00E-05 threshold was used, the numbers of lead SNPs were dropped to 16, 72, 88, and 116 for RFI, DMI, ADG, and MWT, respectively (Table 2). These lead SNPs had FDR < 0.10 except for the 16 lead SNPs for RFI, for which FDRs were between 0.66 and 0.72.

**Fig. 2:**
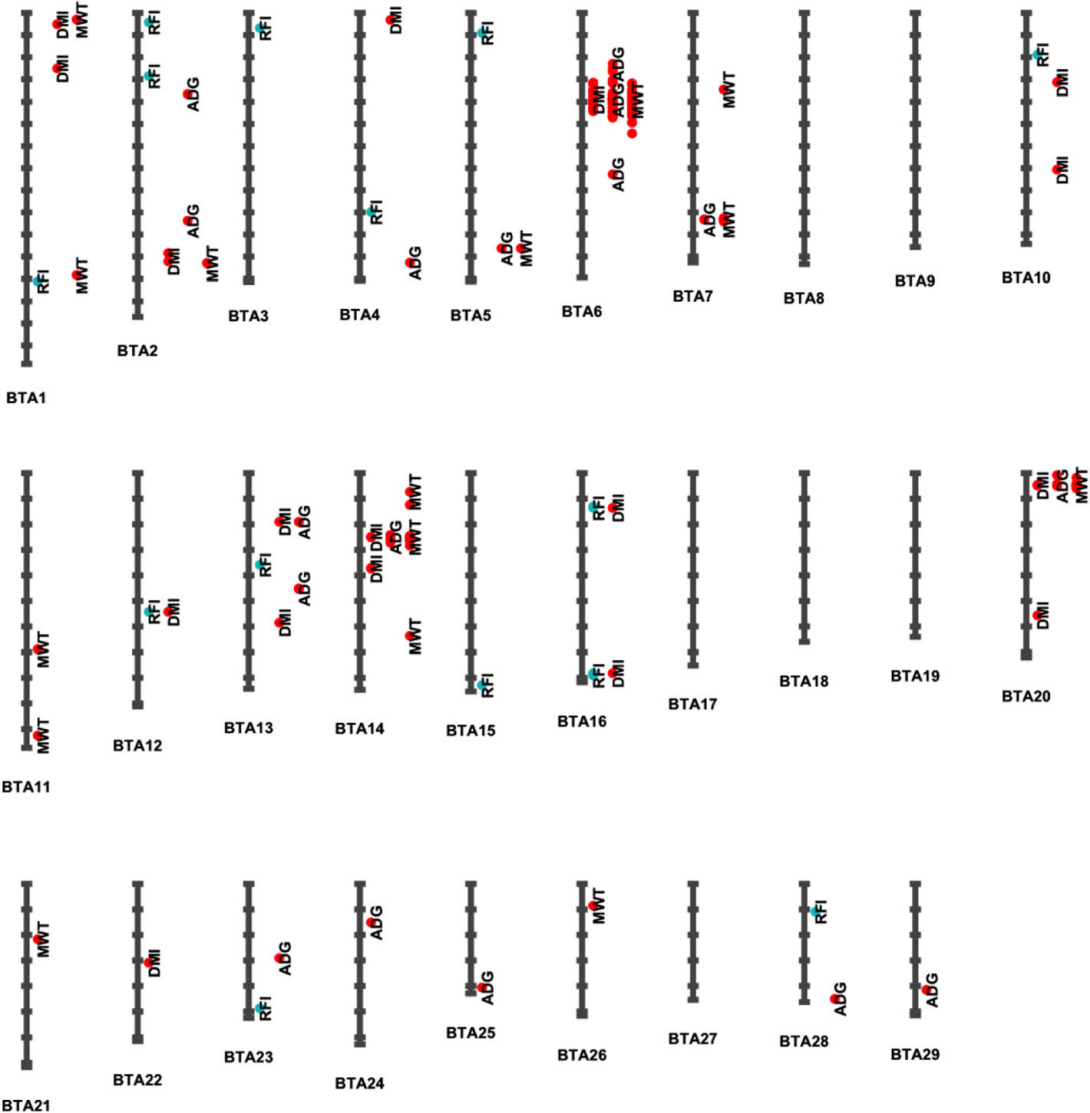
Distribution of lead SNPs at *P*-value < 1.00E-05 on *Bos taurus* autosomes (BTA) for residual feed intake (RFI) and its component traits daily dry matter intake (DMI), average daily gain (ADG), and metabolic body weight (MWT). The blue dot indicates a threshold of *P*-value < 1.00E-05 while the red dot shows the threshold of both *P*-value < 1.00E-05 and genome-wise false discovery rate (FDR) < 0.10

The 16, 72, 88, and 116 lead SNPs for RFI, DMI, ADG, and MWT were distributed on multiple chromosomes for all four traits as depicted in Fig. 2. These lead SNPs explained from 0.24 to 5.8% of the phenotypic variance per SNP for the traits. Top significant lead SNPs of each chromosome that explained more than 0.30% phenotypic variance were presented in Table 4. For RFI, 12 of the 16 lead SNPs explained more than 0.30% phenotypic variance, with 3 SNPs located within a gene. The top lead SNP rs110523019 was located on chromosome 3, explaining 0.43% phenotypic variance. This SNP was annotated to an intronic region of gene *DDR2*. For DMI, 11 of the 72 lead SNPs explained from 0.31 to 3.04% of the total phenotypic variance (Table 4). The lead SNPs for DMI were located on 11 different chromosomes (Table 4), with 8 SNPs annotated to regions between genes and 3 located in an intron or downstream of a gene. SNP rs207689046, which accounted for 3.04% phenotypic variance, was annotated to 113,247 bp from downstream of gene *LCORL*. Lead SNPs on multiple chromosomes were also found to be associated with ADG and MWT (Table 4). Of the 12 lead SNPs that explained more than 0.30% of phenotype variance for ADG, 3 SNPs were annotated to a gene or downstream of a gene. Top lead SNPs rs110987922 and rs134215421 accounted for a relatively large proportion of 4.23 and 1.09% phenotypic variance, respectively. The SNP s110987922 was annotated to 121,223 bp of gene *LCORL* and SNP rs134215421 was located 1166 bp downstream of gene *PLAG1*. For MWT, 10 of the 116 lead SNPs from 10 chromosomes explained more than 0.30% phenotypic variance. Of the 10 top lead SNPs, 6 SNPs were located within a gene while 1 SNP was annotated to downstream of a gene. SNP Chr6:39111019 was the top lead SNP for MWT, accounting for 5.80% of phenotypic variance. This SNP was annotated to 118,907 bp downstream from gene *LCORL*.

**Table 4:** A summary of top lead SNPs of each chromosome in significant associations with RFI and its component traits DMI, ADG, and MWT based on the imputed 7.8 M WGS variant GWAS with a threshold value of *P*-value < 10^−5^ (1.00E-05) in a beef cattle multibreed population

Trait^1^	Lead SNP	Num^2^	Chr	Pos (bp)	Nearest Gene^3^	Distance (bp)^4^	Annotation^5^	P-value	FDR^6^	b ± SE^7^	Var_Phe (%)^8^
RFI	rs109479784	38	1	121,176,492	SNORA70	50,597	intergenic_region	8.27E-06	7.23E-01	− 0.06 ± 0.01	0.35
RFI	rs379241952	41	2	28,511,594	B3GALT1	109,859	intergenic_region	9.69E-07	7.23E-01	0.10 ± 0.020	0.43
RFI	rs110523019	123	3	6,835,555	DDR2	Within	intron_variant	1.74E-07	6.60E-01	−0.14 ± 0.03	0.43
RFI	rs42645457	7	4	89,834,757	GPR37	11,146	intergenic_region	6.12E-06	7.23E-01	−0.07 ± 0.02	0.35
RFI	rs446215391	24	5	9,075,556	SYT1	13,259	intergenic_region	6.77E-07	7.23E-01	0.14 ± 0.03	0.39
RFI	Chr10:18890829	1	10	18,890,829	ENSBTAG00000033344	Within	intron_variant	4.70E-06	7.23E-01	−0.12 ± 0.03	0.36
RFI	rs382972340	154	12	54,262,083	U6	61,906	intergenic_region	8.21E-06	7.23E-01	0.10 ± 0.02	0.33
RFI	rs382536070	1	13	35,856,785	LYZL1	61,216	intergenic_region	6.60E-06	7.23E-01	−0.18 ± 0.04	0.30
RFI	Chr15:82875910	23	15	82,875,910	ENSBTAG00000039917	270	upstream_gene_variant	6.20E-06	7.23E-01	0.18 ± 0.04	0.30
RFI	Chr16:13105979	86	16	13,105,979	RGS2	60,759	intergenic_region	8.38E-06	7.23E-01	−0.07 ± 0.02	0.38
RFI	rs382491772	2	23	48,775,591	F13A1	Within	3’UTR_variant	8.90E-06	7.23E-01	0.14 ± 0.03	0.30
RFI	rs209862831	4	28	10,939,077	ENSBTAG00000046453	4795	upstream_gene_variant	7.19E-06	7.23E-01	0.10 ± 0.02	0.32
DMI	rs211318336	5	1	25,084,372	U2	266,897	intergenic_region	8.30E-06	3.41E-02	−0.18 ± 0.04	0.31
DMI	rs109570141	142	2	112,157,337	U6atac	104,324	intergenic_region	1.96E-06	1.19E-02	0.14 ± 0.03	0.40
DMI	rs472695088	8	4	3,153,240	SNORA31	367,620	intergenic_region	2.80E-06	1.57E-02	−0.20 ± 0.04	0.37
DMI	rs207689046	139	6	39,105,359	LCORL	113,247	intergenic_region	2.77E-25	8.49E-19	0.25 ± 0.02	3.04
DMI	rs109256612	64	10	31,282,009	DPH6	172,888	intergenic_region	7.06E-06	3.10E-02	0.09 ± 0.02	0.35
DMI	rs382972340	154	12	54,262,083	U6	61,906	intergenic_region	1.03E-06	8.63E-03	0.15 ± 0.03	0.40
DMI	rs384869645	425	13	19,004,111	PARD3	Within	intron_variant	5.56E-06	2.64E-02	−0.13 ± 0.03	0.34
DMI	rs110092040	32	14	24,973,953	MOS	1997	downstream_gene_variant	1.12E-08	2.61E-04	−0.15 ± 0.03	0.69
DMI	rs380573663	87	16	78,179,941	CRB1	Within	intron_variant	3.66E-06	1.94E-02	−0.13 ± 0.03	0.36
DMI	rs43357086	73	20	4,791,751	5S_rRNA	14,480	intergenic_region	4.33E-09	1.20E-04	0.12 ± 0.02	0.66
DMI	rs211404023	41	22	30,879,104	5S_rRNA	74,025	intergenic_region	3.71E-06	1.96E-02	−0.09 ± 0.02	0.35
ADG	rs134607538	155	2	93,780,831	ENSBTAG00000010293	77,522	intergenic_region	2.44E-07	1.30E-03	−0.02 ± 0.00	0.47
ADG	Chr4:112725016	34	4	112,725,016	CUL1	31,822	intergenic_region	3.79E-06	1.39E-02	−0.03 ± 0.01	0.37
ADG	rs137822220	151	5	106,247,266	CCND2	6641	intergenic_region	8.25E-07	3.53E-03	0.03 ± 0.01	0.43
ADG	rs110987922	124	6	39,113,335	LCORL	121,223	intergenic_region	3.28E-37	1.74E-30	0.07 ± 0.01	4.23
ADG	rs109901274	62	7	93,244,933	ARRDC3	Within	missense_variant	8.44E-08	5.14E-04	0.03 ± 0.00	0.59
ADG	rs41693642	65	13	45,111,501	ENSBTAG00000046128	57,267	intergenic_region	1.32E-07	7.58E-04	−0.03 ± 0.01	0.48
ADG	rs134215421	59	14	25,006,125	PLAG1	1166	downstream_gene_variant	4.82E-13	1.58E-08	−0.04 ± 0.01	1.09
ADG	rs42661323	50	20	4,916,731	STC2	Within	missense_variant	3.65E-09	4.17E-05	0.03 ± 0.01	0.67
ADG	rs111029508	52	24	15,100,338	snoU54	472,490	intergenic_region	5.47E-06	1.89E-02	0.02 ± 0.00	0.38
ADG	rs448890458	6	25	40,587,255	CARD11	373,301	intergenic_region	5.31E-06	1.86E-02	−0.06 ± 0.01	0.32
ADG	rs469759962	15	28	45,058,986	TMEM72	5434	intergenic_region	9.68E-06	2.96E-02	−0.05 ± 0.01	0.31
ADG	rs137389740	32	29	41,512,334	SCGB1A1	7615	intergenic_region	3.74E-06	1.37E-02	−0.02 ± 0.00	0.36
MWT	rs210255011	37	1	118,345,325	ERICH6	26,609	intergenic_region	3.85E-06	8.48E-03	0.60 ± 0.13	0.40
MWT	rs43320298	7	2	113,058,683	ENSBTAG00000040156	73,281	intergenic_region	7.62E-07	2.07E-03	−1.36 ± 0.28	0.43
MWT	rs110358394	58	5	106,266,665	CCND2	Within	intron_variant	3.20E-07	1.08E-03	0.84 ± 0.17	0.46
MWT	Chr6:39111019	179	6	39,111,019	LCORL	118,907	intergenic_region	1.59E-44	5.39E-38	2.30 ± 0.16	5.80
MWT	rs109901274	88	7	93,244,933	ARRDC3	Within	missense_variant	9.61E-09	5.22E-05	0.76 ± 0.13	0.70
MWT	rs446606774	4	11	68,821,419	GALNT14	Within	intron_variant	7.92E-07	2.12E-03	1.97 ± 0.40	0.39
MWT	rs134215421	59	14	25,006,125	PLAG1	1166	downstream_gene_variant	2.08E-28	1.59E-23	−1.91 ± 0.17	2.69
MWT	rs41934045	193	20	4,563,925	ERGIC1	Within	intron_variant	6.12E-21	2.52E-16	1.42 ± 0.15	1.77
MWT	rs209660822	74	21	21,679,784	AP3S2	Within	3’UTR_variant	8.25E-06	1.65E-02	0.74 ± 0.17	0.35
MWT	rs133223744	6	26	8,545,128	A1CF	Within	intron_variant	3.27E-06	7.45E-03	2.12 ± 0.45	0.30

^1^*RFI* residual feed intake in kg of DMI per day, *DMI* daily dry matter intake in kg per day, *ADG* average daily gain in kg, *MWT* metabolic body weight in kg

^2^The number of significant support SNPs associated with a lead SNP within 70 k bps

^3^The nearest annotated gene to the significant SNP. The annotated gene database was downloaded from https://www.ensembl.org/index.html

^4^SNP designated as in a gene or distance (bp) from a gene region in the UMD3.1 bovine genome assembly

^5^Functional annotation for the SNP

^6^FDR = genome-wise false discovery rate (FDR) calculated followed the Benjamini-Hochberg procedure [32]

^7,8^The allele substitution effect (b) ± standard error (SE) and phenotypic variance explained by the significant SNP, respectively

### Functional enrichment analysis

With the lead significant SNPs for each trait in Table 2, 596, 268, 179, and 532 candidate genes were identified as candidate genes for RFI, DMI, ADG, and MWT, respectively, based on UMD3.1 bovine reference genome annotated autosomal genes (23,431 genes in total) that were downloaded from the Ensembl BioMart database (accessed November 8, 2018). Of the identified candidate genes, 179 unique genes were common to all traits, and 576, 257, 171, and 514 genes for RFI, DMI, ADG, and MWT, respectively, were mapped to the IPA database. In total, we identified 26 cellular and molecular functions for RFI, 25 for DMI, and 27 for both ADG and MWT at a *P*-value < 0.05 as presented in (Additional file 1: Figure S2 to Figure S5). Of the top 5 enriched molecular and cellular functions, lipid metabolism was highly enriched for all four traits. Cell morphology and molecular transport were common between RFI and MWT, whereas nucleic acid metabolism and carbohydrate metabolism were common to DMI and ADG. Additionally, small molecule biochemistry was common to ADG, DMI, and MWT. Table 5 listed genes involved in each of the top five enriched molecular and cellular biological functions for each trait.

**Table 5:** Five topmost significantly enriched biological functions for RFI and its component traits, and genes involved in the specific function

Trait^a^	Biological Function	Genes Involved in the biological function
RFI	1-Cell Morphology	*AMPH, ARHGAP32, ATL1, BID, C3AR1, CAMP, CCND1, CD4, CFTR, CHL1, CLEC11A, CLIC4, CNTFR, CSTB, CTHRC1, CUL3, DVL1, EPO, FGL1, GDF3, GSDMD, HAND1, HAUS4, HELLS, IFNA2, INHA, INTU, KCNE2, KCNK2, KIF11, KIF13B, KIFC1, LGALS1, LIF, LIMK2, MAPK1, MAPT, MMP20,NDUFAB1,NEFHNFIA, NGFR, NMNAT3, NTRK2, OSMR, P2RY12, PALLD, PARD3, PARD6B, PCTP, PEG10, PKP1, PLXNB2, POLG, PPARGC1A, PPARGC1B, PTHLH, PTPN1, RIPK1, RNF4, RXRB, SCYL1, SERPINA3, SERPINE2, SGCE, TRAK2, UCP1UPK2, VDAC1*
	2-Cellular Assembly and Organization	*ADNP, ATG4B, ATG4C, BID, CAMP, CBLB, CCND1, CD4, CHL1, CLEC11A, CLIC4, CTHRC1, ENC1, EXO5, FSCN1, HAND1, IDE, KCNK2, KIF11, KIFC1, KLHDC8B, LANCL1, LGALS1, MAPT, NDUFAB1, NDUFS2, NEFH, NGFR, NLGN1, NR5A1, NTRK2, OLA1, P2RY12, PARD3, POLG, PPARGC1A, PPARGC1B, SERPINA3, SLC25A5, SRCIN1, SS18, SSNA1, TP53INP1, TTR, UCP1, VDAC1*
	3-Cellurar Function and Maintenance	*LANCL1, ST8SIA1, PARD3, ATL1, PPARGC1A, CCDC103, PARD6B, CD4, SS18, RIPK1, CELSR2, CLEC11A, UCP1, COQ7, CTHRC1, LIF, SERPINA3, NFIA, NDUFAB1, CCDC39, EPO, CHL1, CCND1, BID, KCNK2, VDAC1, LGALS1, TP53INP1, TCF7L1, CAMP, KIF11, KIF13B, P2RY12, NLGN1, PPARGC1B, POLG, PLXNB2, ARMC4, CLIC4, MAPT, NTRK2, IFNA2, FSCN1, TRAK2, DVL1, NMNAT3, HAND1, NEFH, NDUFS2, IDE, FCGR2B, NGFR, ARHGAP32, KIFC1, TFCP2L1*
	4-Lipid Metabolism	*ABHD3, ACSL6, AGMO, AKR1C3, AKR1C4, AKR1C1/AKR1C2, ALPI, ANGPTL4, ANGPTL6, ATP5PF, BID, BMP7, CAMP, CD4, CERS5, CFTR, CLDN16, CLEC11A, CNTFR, CTDNEP1, CYP2C18, CYP2J2, CYP7B1, DEGS2, DHRS4, ELOVL4, ERLIN1, FCGR2B, FGL1, GNAI1, GPC3, IL1RN, INHA, KCNE1B, KIF13B, LGALS1, LIF, MAPK1, MOGAT2, MRAS, NGFR, NR5A1, NTRK2, P2RY12, P2RY13, PARD3, PCTP, PDK2, PIGP, PIK3CB, PLA2G2A, PLEKHA3, PLVAP, POLG, PPARGC1A, PPARGC1B, PRKCB, PTHLH, PTPN1, RGS2, SERPINE2, ST8SIA1, TFCP2L1, TRHR, TTR, UCP1, UGT2B4, UGT2B11, UGT2B17*
	5-Molecular Transport	*GPC3, ST8SIA1, KLF15, MRAS, INHA, PIK3CB, ANGPTL4, CLDN16, PPARGC1A, IL1RN, PDK2, P2RY13, PRKCB, FGL1, CD4, CA4, CTDNEP1, PCSK2, CLEC11A, UCP1, MOGAT2, DIO3, LIF, DUOXA2, SLC37A2, ANGPTL6, PTPN1, HBA1/HBA2, CFTR, BID, PLA2G2A, TTR, GNAI1, KCNE2, VDAC1, ALPI, LGALS1, PKN1, TRHR, CAMP, TP53INP1, KIF13B, PPARGC1B, POLG, CLIC4, NTRK2, NR5A1, BMP7, GCNT4, SLC22A6, PTGER1, KCNE1B, SLC20A2, PCTP, FCGR2B, AGMO, PLVAP, NGFR, IP6K1, MAPK1, AOC3, GRPR, PTHLH*
DMI	1-Cabohydrate Metabolism	*GNAI1, ST8SIA1, VDAC1, ALPI, PARD3, LGALS1, MRAS, KDM8, PIK3CB, PIGP, PPARGC1A, PLEKHA3, PPARGC1B, PDK2, PRKCB, BMP7, UGT2B17, CYP2J2, PCTP, CMAS, NGFR, AGMO, MAPK1, GRPR, BID, PTHLH, PLA2G2A*
	2-Nucleoc Acid Metabolism	*GNAI1, ST8SIA1, VDAC1, NUDT9, ATP5PF, PPARGC1A, GART, CMAS, PDK2, PRKCB, MAPK1, CBLB, GRPR, CFTR, BID, OLA1, PTHLH, BMP7*
	3- Small molecule Biochemistry	*ST8SIA1, PARD3, MRAS, INHA, PIK3CB, DHRS4, PIGP, C3AR1, ACSL6, PPARGC1A, PDK2, P2RY13, PRKCB, CBLB, PCSK2, CLEC11A, MOGAT2, TGM1,CYP2J2, AKR1C4, AKR1C3, CFTR, BID, PLA2G2A, GNAI1, KCNE2, VDAC1, ALPI, LGALS1, NUDT9, ERLIN1, ATP5PF, CNTFR, STC2, PLEKHA3, PPARGC1B, NR5A1, BMP7, GBA3, UPK2, UGT2B17, SLC22A6, KCNE1B, ATP6V1G1, PCTP, CMAS, AGMO, NGFR, MAPK1, GRPR, PTHLH, TFCP2L1*
	4-Cellular Development	*KCNK2, ITIH4, LGALS1, NASP, UGT2B17, ITGA11, KCNE1B, INHA, NFIA, PIK3CB, C3AR1, KR1C3, PPARGC1A, NGFR, MAPK1, CLIC4, BID, PTHLH, NR5A1, TFCP2L1, BMP7, FSCN1*
	5-Lipid metabolism	*GNAI1, ST8SIA1, ALPI, PARD3, LGALS1, MRAS, ERLIN1, ATP5PF, INHA, CNTFR, PIK3CB, DHRS4, PIGP, C3AR1, ACSL6, PPARGC1A, PLEKHA3, PPARGC1B, PDK2, P2RY13, PRKCB, NR5A1, BMP7, GBA3, CLEC11A, MOGAT2, UGT2B17, CYP2J2, KCNE1B, AKR1C4, PCTP, AKR1C3, AGMO, NGFR, MAPK1, CFTR, BID, PTHLH, TFCP2L1, PLA2G2A*
ADG	1-Cabohydrate Metabolism	*ST8SIA1, PARD3, LGALS1, UGT2B17, MRAS, KDM8, PIK3CB, PIGP, PPARGC1A, CMAS, PLEKHA3, NGFR, PPARGC1B, PDK2, MAPK1, GRPR, BID, PTHLH, BMP7, PLA2G2A*
	2-Nucleoc Acid Metabolism	*ST8SIA1, CMAS, UGT2B17, PDK2, MAPK1, GRPR, CFTR, BID, BMP7*
	3-Small molecule Biochemistry	*ST8SIA1, PARD3, LGALS1, MRAS, ERLIN1, CNTFR, KDM8, PIK3CB, DHRS4, PIGP, ACSL6, PPARGC1A, PLEKHA3, PPARGC1B, PDK2, P2RY13, NR5A1, BMP7, GBA3, CLEC11A, MOGAT2, TGM1, UGT2B17, SLC22A6, CMAS, NGFR, MAPK1, GRPR, CFTR, BID, PTHLH, PLA2G2A, TFCP2L1*
	4-Lipid Metabolism	*ST8SIA1, PARD3, LGALS1, MRAS, ERLIN1, CNTFR, PIK3CB, DHRS4, PIGP, ACSL6, PPARGC1A, PLEKHA3, PPARGC1B, PDK2, P2RY13, NR5A1, BMP7, GBA3, CLEC11A, MOGAT2, UGT2B17, TGM1, NGFR, MAPK1, CFTR, BID, PTHLH, TFCP2L1, PLA2G2A*
	5-Cell to cell Signal and interaction	*PARD3, LGALS1, MAPK1, CLIC4, GRPR, CNTFR, CFTR, PTHLH, BMP7*
MWT	1-Cellular Compromise	*LANCL1, HPSE, SERPINE2, LIF, CAMP, SIAH1, SCYL1, EPO, NEFH, GCH1, PPARGC1A, PPARGC1B, MAPT, NTRK2, CFTR, PSMC6, BID, PTHLH*
	2-Lipid Metabolism	*GPC3, UGT2B11, ST8SIA1, CYP7B1, PARD3, INHA, PIK3CB, DHRS4, PIGP, ACSL6, PPARGC1A, IL1RN, P2RY13, PRKCB, RGS2, CTDNEP1, DEGS2, CLEC11A, MOGAT2, UCP1, CYP2J2, LIF, AKR1C4, ELOVL4, AKR1C3, CFTR, BID, PLA2G2A, TTR, GNAI1, ALPI, LGALS1, TRHR, SERPINE2, ERLIN1, ATP5PF, CAMP, CNTFR, P2RY12, CYP2C18, PLEKHA3, PPARGC1B, POLG, NR5A1, BMP7, GBA3, UGT2B4, CERS5, UGT2B17, HAND1, PCTP, FCGR2B, AGMO, NGFR, MAPK1, ABHD3, TFCP2L1*
	3-Molecular Transport	*GPC3, HCRTR1, INHA, PIK3CB, ACSL6, PPARGC1A, IL1RN, PDK2, PRKCB, CA4, PCSK2, CLEC11A, UCP1, COQ7, MOGAT2, DIO3, LIF, DUOXA2, SLC37A2, EPO, HBA1/HBA2, CFTR, PLA2G2A, TTR, GNAI1, KCNE2, VDAC1, LGALS1, PKN1, TRHR, CAMP, TP53INP1, P2RY12, PPARGC1B, POLG, OSCP1, CLIC4, NTRK2, NR5A1, BMP7, GCNT4, UPK2, SLC22A6, PTGER1, SLC20A2, SIAH1, NGFR, MAPK1, AOC3, GRPR, PTHLH*
	4-Small Molecule Biochemistry	*GPC3, HCRTR1, UGT2B11, ST8SIA1, CYP7B1, PARD3, INHA, PIK3CB, DHRS4, PIGP, ACSL6, PPARGC1A, IL1RN, PDK2, P2RY13, ADPRHL2, PRKCB, RGS2, CTDNEP1, PCSK2, DEGS2, CLEC11A, MOGAT2, UCP1, DIO3, CYP2J2, LIF, DUOXA2, AKR1C4, ELOVL4, AKR1C3, EPO, CFTR, BID, PLA2G2A, TTR, GNAI1, KCNE2, ALPI, LGALS1, NUDT9, TRHR, SERPINE2, ERLIN1, ATP5PF, CAMP, CNTFR, TP53INP1, P2RY12, CYP2C18, PLEKHA3, PPARGC1B, POLG, OSCP1, NTRK2, NR5A1, BMP7, GBA3, GCNT4, UGT2B4, CERS5, RENBP, UGT2B17, PTGER1, SIAH1, HAND1, PCTP, FCGR2B, CMAS, AGMO, NGFR, DPYD, MAPK1, ABHD3, GRPR, PTHLH, TFCP2L1*
	5-Cell Morphology	*PARD3, GDF3, AMPH, INHA, C3AR1, SCYL1, PALLD, PPARGC1A, CLEC11A, PEG10, UCP1, CSTB, LIF, SERPINA3, NFIA, NDUFAB1, OSMR, EPO, CHL1, CFTR, BID, KCNE2, KCNK2, INTU, VDAC1, LGALS1, SERPINE2, CNTFR, CAMP, P2RY12, PPARGC1B, POLG, PLXNB2, CLIC4, NTRK2, MAPT, CUL3, IFNA2, GSDMD, CERS5, UPK2, PKP1, RXRB, SGCE, NMNAT3, HAND1, PCTP, NEFH, NGFR, MMP20, MAPK1, PTHLH*

^a^ *RFI* residual feed intake in kg of DMI per day, *DMI* daily dry matter intake in kg per day, *ADG* average daily gain in kg, *MWT* metabolic body weight in kg

To illustrate candidate gene interaction and involvement with biological subfunctions/processes within the major cellular and molecular functions, network diagrams were shown in Additional file 1: Figure S2 to Figure S6. For carbohydrate metabolism that was the top biological function for DMI and ADG, the most enriched subfunctions or processes for both traits included uptake of monosaccharide, oxidation of D-glucose, quantity of inositol phosphate, synthesis of CMP-sialic acid, concentration of phosphatidic acid, synthesis of carbohydrate, and uptake of carbohydrate. Additionally, 20 candidate genes including *PLA2G2A, PARD3, PTHLH, CMAS, GRPR, LGALS1, KDM8, NGFR, PLEKHA3, PIGP, ST8SIA1, PIK3CB, PPARGC1B, PPARGC1A, UGT2B17, PDK2, MRAS, BMP7, BID,* and* MAPK1* were common between DMI and ADG. Cell morphology was the top enriched biological function for RFI with transmembrane potential, transmembrane potential of mitochondria, morphology of epithelial cells, axonogenesis, transmembrane potential of mitochondrial membrane as the major subfunctions/processes. For MWT, cellular compromise was the most significantly enriched function with 18 candidate genes that are important in formation of cellular inclusion bodies, oxidative stress response of the heart and atrophy of different cell types such as muscle and neurons. As lipid metabolism was among the top five enriched functions for the four traits, 24 lipid related candidate genes including *TFCP2L1, CLEC11A, P2RY13, DHRS4, BID, PIK3CB, NGFR, PLEKHA3, ST8SIA1, PARD3, PPARGC1B, CNTFR, ACSL6, MAPK1, MOGAT2, PIGP, BMP7, CFTR, ERLIN1, PLA2G2A, LGALS1, NR5A1, PPARGC1A,* and *UGT2B17* were common to all the traits, and steroid metabolism and synthesis of lipid were found to be major subfunctions/processes across the four traits (Additional file 1: Figure S6).

## Discussion

### Imputation from 50 K SNPs to 7.8 M WGS variants improves the power of GWAS

A whole genome sequence variant panel represents the ultimate DNA marker set to dissect genetic controls of complex traits via genetic analyses including genome wide association studies as whole genome sequence variants contain all causative polymorphisms that contribute to the variation of the trait. However, genotyping all whole genome sequence variants for a fairly large number of individuals may not be practically feasible currently, especially for farm animals such as beef cattle. Therefore, imputation has been commonly used to predict missing genotypes based on a reference population genotyped at a higher density DNA marker panel or to predict genotypes that are not directly assayed when a lower density of DNA markers is genotyped [33]. Genotype imputation accuracy is determined by multiple factors including the DNA marker density of the panel, difference of DNA marker numbers between the genotyped lower density panel and the DNA marker panel of the reference population, the genetic relatedness of the sample population and reference population, minor allele frequency of DNA variants and the imputation method [34, 35]. In this study, we were able to impute the genotype of 11,448 Canadian beef cattle animals from the 50 K SNP genotypes to 38,318,974 SNPs and INDELs based on a reference population of 4059 animals on HD and 1570 animals with whole genome sequence variants from run 5 of the 1000 bull genomes project, and achieved an average imputation accuracy of 96.41% (Additional file 3: Table S2). However, when the accuracy of an individual SNP or INDEL was examined, nearly 17.24% of the imputed SNPs were found to have imputation accuracy lower than 95%. Therefore, with a minimal imputation accuracy of greater than 95% and other data editing criteria including MAF and HW deviation, 7,853,211 sequence variants including 7,497,128 SNPs and 356,083 INDELs were used in this study (all INDELs are collectively referred as SNPs throughout the text for simplicity). For the traits investigated, the imputed 7,853,211 SNPs accounted for more than 21.7% additive genetic variance on average for the four traits in comparison to the 50 K SNPs, ranging from 19.6% for MWT to 27.3% for ADG (Table 1). These results concur with the GWAS results that additional or novel significant SNPs were detected at various thresholds with the 7.8 M sequence variants (Table 2), indicating that the 7.8 M SNP panel is able to capture more additive genetic variance and improve the power of QTL detection on the RFI and its component traits in comparison to the lower density panel of 50 K SNPs.

### SNP effect distributions

The SNP allele substitution effects from the 7.8 M variant GWAS results approximated a bell-shaped distribution for all the traits, and the amounts of additive genetic variance explained by a single DNA marker followed a scaled inverse chi-squared distribution to a greater extent. These distributions, however, may be biased as greater LD between DNA markers of the 7.8 M DNA variant panel is expected and a single DNA marker GWAS was used in this study. Nevertheless, these distributions support the assumptions on normal distributions of SNP effects and a scaled inverse chi-squared distribution for locus-specific variance that were used in many studies [17, 36–38].

The distributions of SNP allele substitution effects and additive genetic variances accounted for by individual SNPs indicate that the majority of SNPs in the 7.8 M variant panel have zero or small effects on RFI and its component traits. If we use the suggestive *P*-value as a threshold to indicate that a SNP has a non-zero effect on the traits, of the 7,853,211 DNA variants an average of 99.4% have no effects on the traits, ranging from 99.4% for MWT to 99.5% for RFI. This suggests that a π value of approximately 99% should be used to shrink proportions of DNA variants to no effects in genetic analyses for the feed efficiency traits when a high density of DNA marker panel is used.

One important aspect of genetic architecture of a quantitative trait is whether the trait is affected by a few genes with large and/or modest effects plus genes with small effects or is affected by many genes with small effects. For RFI, the top significant SNPs explained less than 0.5% phenotypic variance (Table 4), suggesting that the trait is unlikely controlled by a few SNPs with large effects. For the component traits ADG, DMI, and MWT, most of the top significant SNPs accounted for less than 1% of phenotypic variance. However, there were 1, 2, and 3 top significant SNPs explaining more than 1% of phenotypic variance for DMI, ADG, and MWT, respectively, indicating that the traits are likely influenced by a few DNA variants with modest to large effects, supplemented by many DNA variants with small effects.

### SNP effects related to SNP functional classes

Annotation of DNA variants to gene functional classes provides an important step forward to identify causative DNA polymorphisms that contribute to genetic variation of a complex trait. Our DNA variant annotation was based on the overlap with gene features described in the Ensembl database (release 81). We also obtained human orthologues of the gene to enhance the annotation. The annotation of the 7,853,211 DNA variants showed that 66.9% DNA variants were intergenic, followed by 25.3% for intronic variants, 3.6% for upstream gene variants, and 3.2% for downstream gene variants. Synonymous variants, missense variants and 3’UTR variants accounted for 0.41, 0.22 and 0.20% of the total DNA variants, respectively, and other functional classes had less than 0.1% of the total DNA variants (Additional file 3: Table S2). The relative proportions of DNA variants in each functional class are consistent with that annotated based on all raw imputed 38,318,974 DNA variants (Additional file 3: Table S3), indicating that 7,853,211 DNA variants are a good sample of all the genome sequence variants. Further classification of the 7,853,211 DNA variants into 9 broader functional classes showed that the number of SNPs within each functional class ranged from 3309 for 5’UTR variants to 5,251,680 for intergenic region variants, which provides a reasonable sample of DNA variants to evaluate relative importance of each functional class in affecting the traits. The GWAS results showed that missense variants, 3’UTR variants, 5’UTR variants, and other regulatory DNA variants are more important functional classes in terms of their average single DNA variant allele substitution effect. The estimates of the average single DNA variant allele substitution effect may be biased due to the LD between DNA markers of different functional classes and the single DNA marker GWAS used. However, these results are supported by the expectation that missense variants alter the peptide sequence of a protein, and regulatory variants participate in coordinating the expression of genes including their effects on transcription through modifying the binding sites. In addition, 3’UTR variants and 5’UTR variants play roles in regulating gene expression and gene translation [39].

The additive genetic variance captured by each functional class was also estimated by fitting the genomic relationship matrix (GRM) of the functional class and GRM constructed from DNA variants of all other functional classes simultaneously. In each run of additive genetic variance partition, the sum of the additive genetic variances captured by the two GRMs (Table 3) was almost identical to the additive genetic variance obtained by the GRM with all the imputed 7.8 M DNA variants for all the traits (Table 1), indicating a reliable partition of additive genetic variance of the functional class variants. It was shown that the intergenic region and intron variants accounted for a greater amount of total additive genetic variance for all the traits. Their amount of additive genetic variance captured per sequence variant was, however, smaller than other functional classes for all the traits investigated. These results are in agreement with the report by Koufariotis et al. [40] that the intron and intergenic variants explained the lowest proportion of the genetic variance per SNP basis for milk and fertility traits in dairy cattle, which is likely due to much larger number of DNA variants in the class and majority of them have small or zero effects on the traits. Notable, 3’UTR and synonymous variants captured a greater amount of additive genetic variance per sequence variant for all the traits, which are in line with the results reported by Koufariotis et al. [41] for milk production traits in dairy cattle. These results indicate a greater role of 3’UTR variants in affecting the complex traits, which is also supported by previous studies that have shown that microRNAs interact with target sites in 3’UTR of mRNA to regulate their gene expressions [42, 43].

Koufariotis et al. [41] also found that the “Splice sites” DNA variants explained more genetic variance per sequence variant than other annotation classes for the dairy traits. In this study, DNA variants in splice sites were classified into other regulatory DNA variants, and they also showed a greater amount of additive genetic variance per sequence variant for ADG. In addition, variants in 5’UTR regions and missense variants were found to account for a relatively larger amount of additive genetic variance for RFI. This relative greater importance of 5’UTR variants in explaining additive genetic variance per sequence variant was also observed for milk production traits in dairy cattle by Koufariotis et al. [41]. However, for missense variants Koufariotis et al. observed a small proportion of genetic variance explained per sequence variant for all the milk production traits [41]. In this study, missense variants had a relatively greater amount of additive genetic variance captured per sequence variant for RFI but smaller amounts of additive genetic variance per sequence variant for other feed efficiency related traits including DMI, ADG, and MWT, suggesting that the relative importance of some functional classes may be trait specific.

Although the intergenic region and intron variant classes had relative smaller effects on the feed efficiency traits, most of the lead SNPs with larger allele substitution effects were located within the intergenic region or intron variants (Table 4). We also examined the support SNPs nearby the lead SNPs and found that most the support SNPs were still located in intergenic or intron regions. Further investigation showed that some SNPs in the functional classes of missense variants and other regulatory regions had relatively larger effects in terms of allele substitution effects but their *P*-values were slightly larger than the lead SNP (Additional file 2), which is likely due to their relatively lower MAF and a larger standard error for their allele substitution effect estimates. Therefore, some SNPs in more important functional classes such as missense variants and regulatory region variants did not show as lead SNPs due to their relative larger *P*-values of significance tests. In addition, the imputed 7.8 M DNA variants are still a sample of whole genome DNA variants. Therefore, the intergenic or intronic SNPs with larger effects are likely in high LD with the causative DNA variant(s) that are not included in the 7.8 M DNA variant panel.

### QTLs for RFI and its component traits in beef cattle

QTLs for RFI and its components ADG, DMI, and MWT have been reported on all the autosome chromosomes as summarized in the Cattle QTL database [21]. In this study, with a suggestive threshold of *P*-value < 0.005, we detected a range of 41,248 to 47,923 SNPs associated with RFI and its component traits on all autosomes based on the 7.8 M DNA variant GWAS as shown in the Manhattan plots (Fig. 1). Due to the nature of single SNP GWAS and based on a previous LD study in a Canadian beef cattle population [44], we used a window of 70 k bp to identify a lead SNP with the lowest P-value among clustered significant SNPs whereas other SNPs within 70 k bp upstream and downstream from the lead SNP were considered as support SNPs. All of the lead significant SNPs had a FDR less than 0.10 ranging from 5.39E-38 for MWT to 3.87E-02 for DMI, except for RFI, for which the 16 SNPs had a range of FDR from 0.66 to 0.72. The relatively smaller number of SNPs associated with RFI with larger *P*-values suggests that RFI as a calculated trait may require more studies to identify biologically relevant candidate genes underlying its component traits.

The lead SNPs at the threshold of *P*-value < 1.00E-05 likely represent the QTL regions that have influence on the traits. These lead SNPs for RFI and its component traits are distributed on multiple chromosomes (Fig. 2). However, no QTLs were detected on BTA 8, 9, 17, 18, 19, and 27 in this study. Of the 16 lead SNPs detected in this study for RFI, only Chr15:82875910 lead SNP on BTA15 was considered overlapped with the QTL (56460) reported in the Cattle QTL database by Saatchi et al. [45] (Additional file 3: Table S5). Of the 72, 88, and 116 lead SNPs for DMI, ADG, and MWT, 0, 21 and 19 SNPs were overlapped with the respective QTL reported in the database within 70 k bp up or 70 k bp downstream window. For ADG, the overlapped QTLs were found on BTA 5, 6, 7, 14, and 20 while for MWT the overlapped QTLs were found on BTA 6 and 20. When we increased the bp window to 1 Mb, additional overlapped QTLs were found for RFI and its component traits DMI, ADG, and MWT on multiple autosomes (Additional file 3: Table S5).

Most of the overlapped SNPs were from QTLs identified by Seabury et al. [46] using actual and imputed 778 K genotypes for 3887 U.S. beef cattle, by Zhang et al. [47] using 3 different GWAS methods (SNP-based, haplotype-based and gene-based) in 1173 Simmental cattle genotyped on the Illumina BovineHD BeadChip, and by Lindholm-Perry et al. [48] using 24 cattle with extreme phenotype values and 406 crossbred beef cattle genotyped on 47 SNPs in and around the *LAP3, NCAPG, and LOC540095* (*LCORL*) gene loci. These overlapped QTLs indicate a greater reliability of the detected significant SNPs for the traits. However, the unique QTL regions could suggest that the underlying causative DNA variants are segregating in the respective populations examined.

### QTL regions and significant lead SNPs that affect multiple traits

To investigate possible pleiotropic effects of SNPs or QTL regions on feed efficiency, we compared the significant lead SNPs with a *P*-value < 1.00E-05 among the four traits. It was found that RFI shared only one lead SNP with DMI (rs382972340), and shared no lead SNPs with ADG or MWT (Additional file 1: Figure S7). This reflects the fact that RFI is phenotypically independent from its component traits ADG and MWT. However, DMI shared 10 lead SNPs with ADG and 15 lead SNPs with MWT while ADG and MWT had 19 lead SNPs in common (Additional file 1: Figure S7). When the lead SNPs of two traits are located within 140 k bp, the QTL with possible pleiotropic effects on the two traits is considered. With this criterion, we found 103 possible pleiotropic SNPs for DMI and ADG traits, 135 possible pleiotropic SNPs for ADG and MWT, and 23 SNPs for all three traits. The chromosome regions that these pleiotropic SNPs represent are graphically depicted in Fig. 2.

The QTL with putative pleiotropic effects between or among DMI, ADG, and MWT may be one of the causes for the observed low to moderate genetic correlations among DMI, ADG, and MWT [13, 14]. QTLs or SNPs with pleiotropic effects were commonly observed in beef cattle quantitative traits [45, 46, 49]. Of the putative pleiotropic QTLs detected in this study, a QTL region on BTA6 showed the largest pleiotropic effect on all three RFI component traits. The lead SNPs that explained 3.04, 4.23, and 5.80% phenotypic variances for DMI, ADG, and MWT, respectively, are located within a window of 7976 bp from 39,105,359 bp to 39,113,335 bp on BTA 6 (Table 4). Gene *LCORL* is the nearest gene to the SNPs (from 113,247 bp to 121,223 bp to the lead SNPs), followed by gene *NCAPG*, which is located 293,308 to 301,284 bp from the lead SNPs. Snelling et al. [50] detected a 570 K bp region, i.e. 37.96–38.53 M on BTA6 spanning *NCAPG*-*LCORL* genes that were associated with DMI and ADG in a crossbred beef cattle population. Lindholm-Perry et al. [48] reported that 16 and 20 markers in the *NCAPG*-*LCORL* region were strongly associated with DMI and ADG, respectively. Furthermore, the differentially expressed genes in the *NCAPG*-*LCORL* region were also identified by Lindholm-Perry et al. [51], which reported a negative correlation between DMI and *LCORL* transcript abundance (*P*-value = 0.05) in adipose tissue, a positive correlation between DMI and *LCORL* transcript abundance (*P*-value = 0.04) and protein expression (*P*-value = 0.01) in muscle tissue, and a positive correlation between *NCAPG* transcript abundance and ADG (*P*-value = 0.04) in muscle tissue. Therefore, *LCORL* and *NCAPG* genes are considered to be candidate genes for the pleiotropic effects on the three traits. Although the lead SNPs are not in either of the genes, 121 support SNPs are within the *LCORL* gene and 122 support SNPs are within *NCAPG*. For *LCORL*, all the 121 support SNPs are located in the intron regions except for rs109572301 in the 3′-untranslated region. For *NCAPG*, 116 SNPs are located in the intronic regions, and 4 SNPs are synonymous variants and 2 SNPs are missense. The missense mutations rs109570900 and rs110251642 in *NCAPG* were simultaneously associated with ADG (FDR = 2.95E-07 and 2.20E-05), DMI (FDR = 1.61E-03 and 1.2E-01) and MWT (FDR = 3.03E-10 and 6.15E-06) in this study (Additional file 2). SNP rs109570900 altering Ile-442 to Met in *NCAPG* was previously proposed as a causative DNA marker associated with carcass and body weight by Setoguchi et al. [52]. Further investigation on genes in the *NCAPG*-*LCORL* region has the potential to identify causative DNA variants with larger effects on DMI and ADG.

Other candidate QTLs that had relatively large pleiotropic effects on DMI, ADG, and MWT included a QTL region on BTA 14 and 20. On BTA 14, the three lead SNPs for the three traits are located within 32,172 bp, and ADG and MWT shared the same lead SNP rs134215421. The genes *PLAG1* and *MOS* are the closest to these lead SNPs. The lead SNPs for ADG and MWT were annotated as the downstream gene variants of *PLAG1* (i.e. within 1166 bp) while the lead SNP for DMI was annotated as the downstream gene variant of gene *MOS* (i.e. within 1997 bp). Pleomorphic adenoma gene 1 (*PLAG1*) regulates many target genes, including a number of growth factors such as insulin-like growth factor 2, and substantial evidence supports the gene as a regulator of growth and reproduction [53]. *MOS* is a serine/threonine kinase involved in the MAPK signaling pathway and oocyte meiosis. Gene Ontology (GO) annotations related to this gene mainly focus on biological processes including transferase activity, transferring phosphorus-containing groups and protein tyrosine kinase activity. DNA variants downstream of a gene have been reported to have roles in regulating gene expression or gene translation [54]. On BTA 20, the three lead SNPs are not the same but the lead SNPs for DMI and ADG are located within 124,980 bp and the lead SNP for MWT is located within 352,806 bp of the region. There are multiple genes in this chromosomal region including *STC2*, *5S_rRNA*, *NKX2–5*, *BNIP1*, *CREBRF*, *RPL26L1,* and *ERGIC1*. However, the SNP rs42661323 accounted for 0.67% phenotypic variance of ADG (*P*-value = 3.65E-09, FDR = 4.17E-05) and is located within gene *STC2* as a missense mutation. The SNP also showed significant effects on DMI (*P*-value = 2.54E-07, FDR = 2.86E-03) and MWT (*P*-value = 8.92E-21, FDR = 3.59E-16) and it explained 0.53 and 1.73% phenotypic variance for DMI and MWT, respectively (Additional file 2). The missense mutation at *STC2* c.178C > G alters the coding amino acid at position 60 from a Proline to an Alanine (p.P60A). *STC2* is a homologue of a glycoprotein hormone playing a role in cell proliferation in multiple cancers [55]. Overexpression of *STC2* gene was shown to down-regulate postnatal growth [56], and reduce bone development and skeletal muscle growth [57] in mice. Notably, *STC2* also played a role in adiposity and obesity in nondiabetic humans [58]. Therefore, *STC2* is considered as the primary positional candidate gene on BTA20 with pleiotropic effects for ADG, DMI and MWT.

On other chromosomes, a few significant SNPs with relatively large pleiotropic effects on DMI, ADG, and MWT were also observed. The lead SNP (rs109901274), a missense variant in *ARRDC3* on BTA7, accounted for 0.59% for DMI (*P*-value = 8.44E-08, FDR = 5.14E-04) and 0.70% for MWT (*P*-value = 9.61E-09, FDR = 5.22E-05) (Additional file 2). The arrestin family member *ARRDC3* was reported as a regulator of growth and progression by affecting β-4 integrin (*ITGβ4*) internalization and degradation. In addition, the lead SNP (rs209660822) explained 0.19% for DMI (*P*-value = 1.17E-03, FDR = 6.49E-01) and 0.35% for MWT (*P*-value = 8.25E-06, FDR = 1.65E-02) (Additional file 2). This lead SNP was annotated as 3’UTR_variant of *AP3S2* gene, which may be related to type 2 diabetes mellitus (T2DM) in humans [59]. The SNPs or QTL regions with larger effects and other SNPs that showed a pleiotropic effect on two or more traits in the supplementary excel file (Additional file 2) provide a valuable reference to further investigate causative DNA variants that affect feed efficiency traits in beef cattle.

### Genetic network compared with RNAseq

Understanding of enriched molecular processes, pathways and gene networks associated with complex traits will help shed some light on the underlying genetic mechanisms and associated genes. These enriched molecular processes are usually inferred based on global transcriptome analyses such as microarray and RNAseq studies. Global transcriptome analyses typically target specific tissues and they focus more on differences of transcripts between groups of extreme phenotypes [60, 61]. On the other hand, GWAS has the potential to identify DNA variants of genes that are associated with an expressed phenotypic trait, and therefore enriched molecular processes, functions, pathways and gene networks can be inferred from the identified candidate genes through functional enrichment analyses. However, the feasibility of GWAS to identify gene variants that regulate the trait largely relies on the power to identify significant DNA markers associated with the traits and also relies on the LD of the DNA markers with the causative gene mutations. With the 7.8 M WGS sequence variants and a sample size of 7573, we were able to identify 596, 268, 179, and 532 candidate genes for RFI, DMI, ADG, and MWT, respectively, using a threshold of *P*-value < 1.00E-05 and a LD window of 70 k bp from the lead SNPs. These candidate genes provide a reasonable reference to infer enriched biological functions for the feed efficiency related traits.

The greater similarity of the top 5 enriched molecular processes between RFI and MWT, and between ADG and DMI are a reflection of more candidate genes shared between RFI and MWT, and between ADG and DMI (Additional file 1: Figure S7). For RFI and MWT, the common cell related functions including cell morphology, cellular assembly and organization, cellular function and maintenance suggest that energy expenditure in cell maintenance is more associated with MWT, and thus determines RFI. In beef cattle, studies have shown that over 70% of energy is for maintenance [12], which supports the enriched molecular processes identified in this study. For ADG and DMI, carbohydrate metabolism is the top shared enriched molecular process for the two traits. Carbohydrate metabolism has also been identified to be the enriched molecular process for ADG and DMI by a jejunum transcriptome analysis [62], indicating that carbohydrate utilization efficiency plays a role in determining the amount of DMI and growth rate.

Of the five major enriched molecular and cellular biological functions, lipid metabolism was common to all the four traits, with synthesis of lipids including triglycerides appearing as a very important subfunction or process. Lipid metabolism has also been identified as an enriched molecular process for feed efficiency traits in beef cattle in several transcriptome studies [63–65]. In finishing beef cattle, deposition of fat requires more energy than protein because protein synthesis is energetically more efficient than fat synthesis [12, 66]. Although a greater maintenance requirement is needed for protein due to protein turnover [66], energy required to deposit fat may play a major role in determining feed efficiency in growing beef cattle. Other studies have also demonstrated that less efficient beef cattle tend to have more backfat and intramuscular fat [13, 63, 65, 67]. Therefore, candidate genes involved in lipid metabolism are of greater interest to pursue to identify causative DNA variants for feed efficiency in beef cattle.

## Conclusions

The imputed 7,853,211 WGS DNA variants captured more genetic variance and the GWAS using the WGS DNA variants identified additional QTL regions associated with the feed efficiency traits in comparison to the 50 K SNPs. The 7.8 M WGS variant GWAS results showed that the SNP allele substitution effects followed a bell shaped distribution while the additive genetic variance explained by individual DNA variants conformed to a scaled inverse chi-squared distribution to a greater extent for all of the feed efficiency traits. On average, missense, 3’UTR, 5’UTR, and other regulatory variants exhibited larger allele substitution effects in comparison to DNA variants that are located between genes and in intronic regions. Intergenic and intronic variants also accounted for a smaller amount of additive genetic variance per DNA variant whereas single regulatory and synonymous variants play a more important role in determining variation of the feed efficiency traits. Residual feed intake in beef cattle is controlled by many DNA variants with relatively small effects whereas DMI, ADG, and MWT are influenced by a few DNA variants with large or modest effects plus many DNA variants with small effects. Lipid metabolism and carbohydrate metabolism were identified as the top enriched cellular and molecular functions for the feed efficiency related traits. The genetic architecture as revealed by the GWAS of the imputed 7,853,211 DNA variants will help improve our understanding on the genetic control of feed efficiency traits in beef cattle, which also represents a step forward to identify causative DNA variants for the traits in beef cattle.

## Methods

### Cattle population and phenotype collection

Animals used in this study were part of previous research projects with multiple Canadian beef cattle populations including Angus, Charolais, Kinsella Composite, Elora crossbred, commercial crossbred from the Phenomic Gap Project (PG1) that consisted of AAFC Lacombe Research and Development Centre crossbreds, and commercial terminal crossbreds (TX/TXX). Descriptions of the herds/populations, cattle breeding, and management of the animals were previously described [13, 14, 26, 44]. Briefly, Angus, Charolais, and Kinsella Composite herds are located at the Roy Berg Kinsella Research Ranch, University of Alberta. The Angus and Charolais herds were mated via artificial insemination and live clean-up bulls registered by the Canadian Angus and Charolais Associations, respectively. The Kinsella Composite herd was produced from crosses between Angus, Charolais, or Alberta Composite bulls and the University of Alberta’s Composite dam line. The crossbred population from the Elora Beef Research Centre, University of Guelph, was made by crossing Angus, Simmental, Charolais, and other cattle breeds. The commercial crossbred (PG1) and terminal crossbred (TX/TXX) animals were from multiple commercial herds with Angus, Charolais, Hereford, Simmental, Limousin, and Gelbvieh being the major breeds used in commercial crossbred beef production.

Phenotypic data were collected on animals from 1998 to 2006 for the Elora crossbred, from 2002 to 2015 for Kinsella Composite, from 2004 to 2015 for Angus and Charolais, and from 2008 to 2011 for PG1 and TX/TXX populations. Feed intake was measured for finishing calves (steers and heifers) using the GrowSafe system (GrowSafe Systems Ltd., Airdrie, Alberta, Canada) at their respective feedlot test stations for a period of 76 to 112 days. Serial body weights (BW) in kg were measured for each animal at the beginning and end of the test and at approximately 14-day interval during the test. The daily dry matter intake (DMI) in kg was calculated as an average of dry matter intake over the test period and was further standardized to 12 MJ ME per kg dry matter for finishing steers and heifers, and 10 MJ ME per kg dry matter for replacement heifers based on the energy content of the diet. The initial BW at the start of the test and average daily gain (ADG) were derived from a linear regression of the serial BW measurements against time (day). The metabolic BW (MWT) in kg was calculated as midpoint BW^0.75^, where the midpoint BW was computed as the sum of the initial BW and the product of ADG multiplied by half of the days on test. Residual feed intake (RFI) in kg of DMI per day was computed as the difference between the standardized daily DMI and the expected DMI that was predicted based on animal’s ADG and MWT. The detail of GrowSafe data quality control and calculation of DMI, ADG, MWT, and RFI was described in previous reports [13, 14, 68, 69]. The phenotype data obtained from each data source were examined and phenotypic values outside the range of 3 standard deviations of the mean were excluded.

### SNP data consolidation and population admixture analyses

Animals with phenotype data from the above herds were also genotyped on bovine 50 K SNP panels under previous projects. In this study, SNP data from various data sources were combined with the same SNP format. Furthermore, to ensure that SNP genotypes from different data sources were merged correctly, alleles of each SNP were examined after merging to ensure that each SNP had only two alleles as expected. In total, 50 K SNP genotypes from 11,448 beef cattle were compiled with a SNP genotype call rate greater than 90%. SNP quality control was also conducted for each data source, where SNPs that had a minor allele frequency less than 0.05, had a missing rate larger than 0.05, or were significantly deviated from Hardy-Weinberg equilibrium test (HWT) (*P*-value < 0.001) were excluded from further analyses. After SNP data editing, 33,321 SNP were retained for further analyses. Sporadic missing SNP genotypes in the SNP data set (< 0.065%) were then imputed via the population-based algorithm implemented in Beagle 3.3.2 [70].

With the 50 K SNP panel (33,321 SNPs) on 11,448 beef cattle, a principal component analysis was conducted to examine the population admixture of the Canadian beef cattle, and the breed composition of each animal was predicted using Admixture software [71]. The principal component analysis confirmed the clustering of known purebred Angus and Charolais populations and presence of crossbreds as shown in (Additional file 1: Figure S8). To predict breed composition for each animal, the known pure breeds Angus and Charolais cattle were randomly assigned into one of 5 validation group and the breed composition of Angus and Charolais for each validation group were predicted using the Admixture software [71]. A K = 6 for the postulated ancestral breed number was found to yield the most accurate breed composition prediction for the Angus and Charolais animals. The composition fraction of each of the 6 postulated ancestral breeds was then predicted for each animal in the data set. Subsequently, phenotypic values were adjusted for animal birth year, sex type, a combination of feedlot test location and pen, breed composition fraction of each postulated ancestral breed, and animal age on test for RFI and its component traits DMI, ADG, and MWT. After removing the animals without phenotypic records, a final combined dataset of 7573 animas with the feed efficiency traits and the SNP genotypes was used for this study including Angus (*N* = 1162), Charolais (*N* = 717), Kinsella Composite (*N* = 1506), Elora crossbred population (*N* = 775), commercial crossbred PG1 (*N* = 1911), and commercial TX/TXX strain terminal crossbreds (*N* = 1502).

### SNP imputation and quality control

Imputation of genotypes from the 50 K SNP panel (33,321 SNPs) to whole genome sequence variants was performed via FImpute 2.2 [72] in a two-step procedure: i.e. first from the 50 K SNP panel (33,321 SNPs) to the Affymetrix Axiom Genome-Wide BOS 1 Array (Affymetrix, Inc., Santa Clara) (termed “HD” hereafter), and then from imputed HD (428,895 SNPs) to the full whole-genome sequence (WGS) variants. A multibreed reference panel made up of 4059 animals genotyped with the Affymetrix HD chip from different Canadian breeds was assembled to impute the 50 K SNP panel (33,321 SNPs) to HD, and a panel of 1570 animals with WGS variants from run 5 of the 1000 Bull Genomes Project [73, 74] was used for imputation from imputed HD to the WGS variants. To start the imputation, the 50 K SNP panel (33,321 SNPs) in the top strand SNP format were converted to Affymetrix HD sequence format based on the DNA strand designation and allele determination in each coding format and using guidelines from Illumina and Affymetrix. The WGS imputation was performed chromosome by chromosome and was submitted in parallel computations to high performance computing facilities at WestGrid (https://www.westgrid.ca/). A dataset of 240 animals with both genotyped 50 K SNP panel (33,321 SNPs) and whole sequence data was used to evaluate the accuracy of imputation in a 5-fold cross validation, assuming that the 240 animals had no whole sequence data. The 240 individuals were randomly split into five groups. Each group or fold of animal (*N* = 48) was then chosen as a validation group in turn, and the rest of individuals were merged into the whole genome sequence reference population to impute WGS genotypes for all animals in the validation group. The accuracy of imputation was calculated as the average proportion of WGS variant genotypes of the animals in the validation group that were correctly imputed assuming that the real genotypes of WGS variants had no errors. In total, 38,318,974 WGS variant genotypes were imputed on all the animals and the average imputation accuracy of each chromosome was provided in (Additional file 3: Table S1), ranging from 93.12% for chromosome (BTA) 12 to 97.13% for BTA2. For each SNP, post-imputation quality control procedures were carried out to filter the imputed WGS variant genotypes if one of the following conditions was met: (1) The concordance rate between the actual 50 K SNP genotypes and WGS variant genotypes of run 5 on the 240 validation animals was less than 95%; (2) The average accuracy of imputation on the 5-fold cross-validation of 240 animals was less than 95%; (3) The imputed WGS variants that are homozygous; (4) Minor allele frequency (MAF) was less than 0.005 in either population/breed; (5) Significant deviations from Hardy–Weinberg exact test at significance levels of *P*-value < 0.00001 in either population/breed. The post-imputation quality control resulted in 7,853,211 DNA variant genotypes that contain 30,155 SNPs from the 50 K SNP genotypes (termed 50 K SNPs or 50 K SNP panel in the text) on all the animals. The 7,853,211 DNA variants included 7,497,128 SNPs and 356,083 INDELs (termed 7.8 M DNA variants or SNPs for simplicity). To facilitate comparison of the 50 K SNPs and 7.8 M DNA variants, the imputed 30,155 SNPs in the 7.8 M DNA variant panel were replaced by their actual genotypes.

### Genome wide association analyses

The GWAS analyses were performed using the *mlma* (mixed linear model association) option as implemented in the GCTA package [75, 76], with the following linear mixed model:

$$ {\mathrm{y}}_{\mathrm{ij}}=\upmu +{\mathrm{b}}_{\mathrm{j}}{\mathrm{x}}_{\mathrm{ij}}+{\mathrm{a}}_{\mathrm{ij}}+{\mathrm{e}}_{\mathrm{ij}} $$

where y_ij_ is the adjusted phenotypic value of the ith animal with the jth SNP (i.e. the ijth animal), b_j_ is the allele substitution effect of the jth SNP, x_ij_ is the jth SNP genotype of animal i and x_ij_ is coded as 0, 1, 2 for genotypes *A*_1_*A*_1_, *A*_1_*A*_2_, and *A*_2_*A*_2_, respectively, a_ij_ is the additive polygenic effect of the ijth animal $\sim N\left(0,\boldsymbol{G}{\sigma}_a^2\right)$, and e_ij_ is the random residual effect $\sim N\left(0,\boldsymbol{I}{\sigma}_e^2\right)$. The GWAS was conducted on the cattle population of 7573 animals for the 7.8 M DNA variant panel on a single SNP marker basis with the genomic relationship matrix **G** (GRM) that was derived as defined in Yang et al. [75, 77]:

$$ {A}_{jk}=\frac{1}{M}{\sum}_{i=1}^M\frac{\left({x}_{ij}-2{p}_i\right)\left({x}_{ik}-2{p}_i\right)}{2{p}_i\left(1-{p}_i\right)} $$

Where *A*_*jk*_ is the off diagonal element of the** G** matrix, i.e. genome relationship of animal *j* and animal *k*, and it represents the diagonal element if j = k, *x*_ij_ = 0, 1, 2 are for genotypes *A*_1_*A*_1_, *A*_1_*A*_2_, and *A*_2_*A*_2_, respectively, *p*_*i*_ is the allele frequency of *A*_2_ at locus *i*, *M* is the total number of SNPs. The **G** matrix was constructed with all the DNA marker genotypes in the 7.8 M DNA variant panel, i.e. the MLM*i* method. As a comparison, GWAS analyses were performed on the 50 K SNP panel using the same approach with the G matrix constructed based on the 30,155 SNPs.

The allele substitution effect of each DNA variant (or SNP) was estimated and the significance test of SNP allele substitution effect was conducted using the GCTA package [75, 77–79]. The phenotypic variance explained by a single SNP was calculated by $\mathrm{Var}\ \left(\%\right)=\frac{2 pq{\beta}^2}{S^2}\ast 100\%$, where *p* and *q* denote the SNP allele frequency of *A*_1_ and *A*_2_, respectively; ß is the SNP allele substitution effect, and 2*pqβ*^2^ is the additive genetic variance, and *S*^2^ is the phenotypic variance.

SNPs that have a nominal *p*-value < 0.005 were considered as suggestively significant SNPs as proposed by Benjamin et al. [80], while SNPs with a nominal *P*-value < 1.00E-05 (i.e.10^− 5^) were classified as significant SNPs based on the recommendation of The Wellcome Trust Case Control Consortium [81]. In addition, genome-wise false discovery rate (FDR) was calculated for each SNP followed the Benjamini-Hochberg procedure [32], which controls the expected proportion of significant results that are false positives in a list of rejected null hypotheses. To identify lead SNPs, significant SNPs were sorted by their *P*-value in non-decreasing order. The SNPs with the lowest *P*-value was identified as the lead SNPs while other SNPs within 70 k bp upstream and downstream from the lead SNP were defined as supportive SNPs. A 70 k bp window was chosen for this study as this was the chromosomal length within which a high LD phase correlation (> 0.77) was maintained across a sample of Canadian beef cattle breeds [44].

To estimate the genomic heritability of a trait, we used the GREML-LDMS approach proposed by Yang et al. [82, 83] that considers linkage disequilibrium (LD) among the DNA markers for both 50 K SNPs and the imputed 7.8 M DNA variant panel, and the variance components were estimated via a restricted maximum likelihood (REML) as implemented in the GCTA package using the above statistical model excluding the single DNA variant effect. The genomic heritability was calculated as a ratio of the additive genetic variance over the phenotypic variance of the trait.

### Functional annotation and inference of genetic architecture based on 7.8 M DNA variant panel

The 7,853,211 DNA variants were assigned to a functional class based on their overlap with gene features described in the Ensembl database (release 81), using an updated version of the NGS-SNP annotation system [84]. Gene function, protein domain and Mendelian disease information from Entrez Gene, UniProt and OMIA (Online Mendelian Inheritance in Animals) were used to assign more specific functional effects to each variant. A summary of number of SNPs in each functional class is provided in (Additional file 3: Table S2) for the 7.8 M DNA variant panel.

Distribution of SNP effects was obtained by plotting SNP allele substitution effects of all the DNA variants in the 7.8 M DNA variant panel, and by plotting the additive genetic variances explained by individual DNA variant in the panel. To examine relative importance of DNA variant functional classes, SNPs were further classified into 9 broad functional classes as shown in (Additional file 3: Table S2), which included intergenic region variants, downstream gene variants, upstream gene variants, synonymous variants, intron variants, missense variants, 3’UTR variants, 5’UTR variants, and other regulatory regions that consisted of splice regions in intron variants, disruptive in-frame deletion, and splice region variants, etc. (Additional file 3: Table S2). The average SNP effect was calculated for each of the 9 functional classes as a mean of squared SNP allele substitution effects for the class. In addition, additive genetic variance captured by each functional class was obtained by fitting the GRM constructed based on the DNA variants of the functional class and the GRM constructed based on the DNA variants of all other functional classes simultaneously in the statistical model using the GCTA package. The amount of additive genetic variance explained per sequence variant was calculated for each functional class by the additive genetic variance captured by the DNA variants in the functional class divided by the number of DNA variants in the class.

### Candidate gene identification and functional enrichment analyses

Genes that are located within 70 k bp upstream and downstream of the lead SNP were considered as candidate genes associated with the trait based on SNP annotation information from the UMD3.1 bovine genome assembly from Ensembl (http://www.ensembl.org/index.html). Ingenuity Pathway Analysis (IPA) web-based software (Redwood City, CA; https://www.qiagenbioinformatics.com/products/ingenuity-pathway-analysis/) (IPA Spring 2019 release) was used for the functional enrichment analyses of the candidate genes identified. Briefly, for the genes with known human orthologues from Ensembl, their gene IDs were replaced with their human orthologous gene IDs, whereas for those without human orthologs their bovine gene IDs were maintained in the gene list. These Ensembl gene IDs were then used as input gene identifiers in IPA and a core analysis was performed on the genes that were mapped to the IPA knowledge base database. Molecular and cellular biological functions were considered significantly enriched if the *P*-value for the overlap comparison test between the input gene list and the IPA knowledge base database for a given biological function was less than 0.05. Additionally, biological processes/subfunctions interaction networks within the most significant molecular and cellular function were generated to show possible biological networks, through which the candidate genes involved in the network were identified.

## Supplementary information

**Additional file 1:** **Figure S1.** Distribution of SNP allele substitution effects (left) and additive genetic variances explained by single SNPs (right) for RFI, DMI, ADG, and MWT based on 7.8 M whole genome sequence (WGS) variants; **Figure S2.** The enriched cellular and molecular functions (top) and cell morphology network (bottom) for RFI; **Figure S3.** The enriched cellular and molecular functions (top) and carbohydrate metabolism network (bottom) for DMI; **Figure S4.** The enriched cellular and molecular functions (top) and carbohydrate metabolism network (bottom) for ADG; **Figure S5.** The enriched cellular and molecular functions (top) and cellular compromise network (bottom) for MWT; **Figure S6.** Lipid metabolism network for RFI and its component traits DMI, ADG, and MWT; **Figure S7.** Common lead significant SNPs with the same IDs (a) and candidate genes (b) among RFI and its three component traits at thresholds *P*-value < 1.00E-05 and FDR < 0.10 based on the imputed 7.8 M DNA variant GWAS; **Figure S8.** Principal component analyses of Canadian beef cattle populations (*N* = 7573) based on the 50 K (a) and 7.8 M WGS (b) panels.

**Additional file 2. **This file contains information of all suggestive significant SNPs/INDELs at P-value < 0.005 based on the 7.8 M sequence variant GWAS for RFI, DMI, ADG, and MWT.

**Additional file 3: Table S1.** Accuracy of genotype imputation from Illumina 50 K SNPs to whole genome sequence (WGS) variant genotypes via HD using Fimpute 2.2; **Table S2.** Functional annotations of 7.8 M WGS variants along with the number of variants in each class, classification of SNP functions, percentage of WGS and 9 functional class assignments; **Table S3.** Functional annotation of all DNA variants (38,318,974) based on DNA variants of the 1000 bulls genome project; **Table S4.** Functional annotations of SNPs in the 50 K SNP panel after quality control along with the number of variants in each class, classification of SNP functions, percentage of WGS, and 9 functional class assignments; **Table S5.** List of lead SNPs that were overlapped with QTLs published in Cattle QTL database for RFI, DMI, ADG, and MWT.

## Data Availability

The datasets supporting the results of this article are included within the article and its additional files. Whole genome sequence datasets generated and/or analyzed during the current study for imputation are available from the NCBI SRA database under BioProjects PRJNA176557 and PRJNA256210. The original genotype and phenotype data sets are available for non-commercial purposes from CL following the execution of a materials transfer agreement.

## Abbreviations

ADG: Average daily gain
BTA: *Bos taurus* autosome
DMI: Dry matter intake
DNA: Deoxyribonucleic acid
FDR: Genome-wise false discovery rate
GRM: Genomic relationship matrix
GWAS: Genome-wide association study
HWT: Hardy-Weinberg equilibrium test
LD: Linkage disequilibrium
MAF: Minor allele frequency
MWT: Metabolic body weight
QTL: Quantitative trait loci
RFI: Residual feed intake
SNP: Single nucleotide polymorphism
WGS: whole genome sequence variants

## References

[1] Shalev BA, Pasternak H (1989). Meat production efficiencies of Turkey, chicken and duck broilers. Worlds Poult Sci J.

[2] Ramsey R, Doye D, Ward C, Mcgrann J, Falconer L, Bevers S (2005). Factors affecting beef cow-herd costs, production, and profits. J Agric Appl Econ.

[3] Van Heugten E. Growing-finishing swine nutrient recommendations and feeding management. National Swine Nutrition Guide (ed DJ, Meisinger) 2010:80–95.

[4] Petty TW, Cecava MJ (1995). Beef cattle feeding and nutrition.

[5] Fitzsimons C, Kenny DA, Deighton MH, Fahey AG, McGee M (2013). Methane emissions, body composition, and rumen fermentation traits of beef heifers differing in residual feed intake. J Anim Sci.

[6] Hegarty R, Goopy J, Herd R, McCorkell B (2007). Cattle selected for lower residual feed intake have reduced daily methane production. J Anim Sci.

[7] Nkrumah JD, Okine EK, Mathison GW, Schmid K, Li C, Basarab JA, Price MA, Wang Z, Moore SS (2006). Relationships of feedlot feed efficiency, performance, and feeding behavior with metabolic rate, methane production, and energy partitioning in beef cattle. J Anim Sci.

[8] Kellner OJ, Goodwin W (1915). The scientific feeding of animals.

[9] Brody S (1945). Bioenergetics and growth; with special reference to the efficiency complex in domestic animals.

[10] Kleiber M (1947). Body size and metabolic rate. Physiol Rev.

[11] Koch RM, Swiger LA, Chambers D, Gregory KE (1963). Efficiency of feed use in beef cattle. J Anim Sci.

[12] Archer J, Richardson E, Herd R, Arthur P (1999). Potential for selection to improve efficiency of feed use in beef cattle: a review. Crop Pasture Sci.

[13] Mao F, Chen L, Vinsky M, Okine E, Wang Z, Basarab J, Crews DH, Li C (2013). Phenotypic and genetic relationships of feed efficiency with growth performance, ultrasound, and carcass merit traits in Angus and Charolais steers. J Anim Sci.

[14] Nkrumah JD, Basarab JA, Wang Z, Li C, Price MA, Okine EK, Crews DH, Moore SS (2007). Genetic and phenotypic relationships of feed intake and measures of efficiency with growth and carcass merit of beef cattle. J Anim Sci.

[15] Chen L, Schenkel F, Vinsky M, Crews DH, Li C (2013). Accuracy of predicting genomic breeding values for residual feed intake in Angus and Charolais beef cattle. J Anim Sci.

[16] Khansefid M, Pryce JE, Bolormaa S, Miller SP, Wang Z, Li C, Goddard ME (2014). Estimation of genomic breeding values for residual feed intake in a multibreed cattle population. J Anim Sci.

[17] Lu D, Akanno EC, Crowley JJ, Schenkel F, Li H, De Pauw M, Moore SS, Wang Z, Li C, Stothard P (2016). Accuracy of genomic predictions for feed efficiency traits of beef cattle using 50K and imputed HD genotypes. J Anim Sci.

[18] Herd R, Arthur P (2009). Physiological basis for residual feed intake. J Anim Sci.

[19] Richardson E, Herd R (2004). Biological basis for variation in residual feed intake in beef cattle. 2. Synthesis of results following divergent selection. Aust J Exp Agric.

[20] Herd R, Oddy V, Richardson E (2004). Biological basis for variation in residual feed intake in beef cattle. 1. Review of potential mechanisms. Aust J Exp Agric.

[21] Hu Z-L, Park CA, Reecy JM (2018). Building a livestock genetic and genomic information knowledgebase through integrative developments of animal QTLdb and CorrDB. Nucleic Acids Res.

[22] Sanchez MP, Govignon-Gion A, Croiseau P, Fritz S, Hoze C, Miranda G, Martin P, Barbat-Leterrier A, Letaief R, Rocha D (2017). Within-breed and multi-breed GWAS on imputed whole-genome sequence variants reveal candidate mutations affecting milk protein composition in dairy cattle. Genet Sel Evol.

[23] Yan G, Qiao R, Zhang F, Xin W, Xiao S, Huang T, Zhang Z, Huang L (2017). Imputation-based whole-genome sequence association study rediscovered the missing QTL for lumbar number in Sutai pigs. Sci Rep.

[24] Frischknecht M, Bapst B, Seefried FR, Signer-Hasler H, Garrick D, Stricker C, Fries R, Russ I, Solkner J, Bieber A (2017). Genome-wide association studies of fertility and calving traits in Brown Swiss cattle using imputed whole-genome sequences. BMC Genomics.

[25] Pausch H, MacLeod IM, Fries R, Emmerling R, Bowman PJ, Daetwyler HD, Goddard ME (2017). Evaluation of the accuracy of imputed sequence variant genotypes and their utility for causal variant detection in cattle. Genet Sel Evol.

[26] Zhang F, Ekine-Dzivenu C, Vinsky M, Basarab JA, Aalhus JL, Dugan MER, Li C (2017). Phenotypic and genetic relationships of residual feed intake measures and their component traits with fatty acid composition in subcutaneous adipose of beef cattle. J Anim Sci.

[27] Arthur PF, Archer JA, Johnston DJ, Herd RM, Richardson EC, Parnell PF (2001). Genetic and phenotypic variance and covariance components for feed intake, feed efficiency, and other postweaning traits in Angus cattle. J Anim Sci.

[28] Arthur PF, Renand G, Krauss D (2001). Genetic parameters for growth and feed efficiency in weaner versus yearling Charolais bulls. Aust J Agric Res.

[29] Crowley JJ, McGee M, Kenny DA, Crews DH, Evans RD, Berry DP (2010). Phenotypic and genetic parameters for different measures of feed efficiency in different breeds of Irish performance-tested beef bulls. J Anim Sci.

[30] Fan LQ, Bailey DR, Shannon NH (1995). Genetic parameter estimation of postweaning gain, feed intake, and feed efficiency for Hereford and Angus bulls fed two different diets. J Anim Sci.

[31] Robinson DL, Oddy VH (2004). Genetic parameters for feed efficiency, fatness, muscle area and feeding behaviour of feedlot finished beef cattle. Livest Prod Sci.

[32] Benjamini Y, Hochberg Y (1995). Controlling the false discovery rate: a practical and powerful approach to multiple testing. J R Stat Soc Ser B Methodol.

[33] Howie BN, Donnelly P, Marchini J (2009). A flexible and accurate genotype imputation method for the next generation of genome-wide association studies. PLoS Genet.

[34] Marchini J, Howie B (2010). Genotype imputation for genome-wide association studies. Nat Rev Genet.

[35] Browning SR (2008). Missing data imputation and haplotype phase inference for genome-wide association studies. Hum Genet.

[36] Chen L, Vinsky M, Li C (2015). Accuracy of predicting genomic breeding values for carcass merit traits in Angus and Charolais beef cattle. Anim Genet.

[37] Rolf MM, Garrick DJ, Fountain T, Ramey HR, Weaber RL, Decker JE, Pollak EJ, Schnabel RD, Taylor JF (2015). Comparison of Bayesian models to estimate direct genomic values in multi-breed commercial beef cattle. Genet Sel Evol.

[38] van Binsbergen R, Calus MP, Bink MC, van Eeuwijk FA, Schrooten C, Veerkamp RF (2015). Genomic prediction using imputed whole-genome sequence data in Holstein Friesian cattle. Genet Sel Evol.

[39] Krebs JE, Goldstein ES, Kilpatrick ST (2009). Lewin’s genes X.

[40] Koufariotis L, Chen YP, Bolormaa S, Hayes BJ (2014). Regulatory and coding genome regions are enriched for trait associated variants in dairy and beef cattle. BMC Genomics.

[41] Koufariotis LT, Chen YP, Stothard P, Hayes BJ (2018). Variance explained by whole genome sequence variants in coding and regulatory genome annotations for six dairy traits. BMC Genomics.

[42] Gu S, Jin L, Zhang F, Sarnow P, Kay MA (2009). Biological basis for restriction of microRNA targets to the 3′ untranslated region in mammalian mRNAs. Nat Struct Mol Biol.

[43] Lai EC (2002). Micro RNAs are complementary to 3′ UTR sequence motifs that mediate negative post-transcriptional regulation. Nat Genet.

[44] Lu D, Sargolzaei M, Kelly M, Li C, Vander Voort G, Wang Z, Plastow G, Moore S, Miller S (2012). Linkage disequilibrium in Angus, Charolais, and crossbred beef cattle. Front Genet.

[45] Saatchi M, Beever JE, Decker JE, Faulkner DB, Freetly HC, Hansen SL, Yampara-Iquise H, Johnson KA, Kachman SD, Kerley MS (2014). QTLs associated with dry matter intake, metabolic mid-test weight, growth and feed efficiency have little overlap across 4 beef cattle studies. BMC Genomics.

[46] Seabury CM, Oldeschulte DL, Saatchi M, Beever JE, Decker JE, Halley YA, Bhattarai EK, Molaei M, Freetly HC, Hansen SL (2017). Genome-wide association study for feed efficiency and growth traits in U.S. beef cattle. BMC Genomics.

[47] Zhang WG, Li JY, Guo Y, Zhang LP, Xu LY, Gao X, Zhu B, Gao HJ, Ni HM, Chen Y (2016). Multi-strategy genome-wide association studies identify the DCAF16-NCAPG region as a susceptibility locus for average daily gain in cattle. Sci Rep.

[48] Lindholm-Perry AK, Sexten AK, Kuehn LA, Smith TPL, King DA, Shackelford SD, Wheeler TL, Ferrell CL, Jenkins TG, Snelling WM (2011). Association, effects and validation of polymorphisms within the NCAPG-LCORL locus located on BTA6 with feed intake, gain, meat and carcass traits in beef cattle. BMC Genet.

[49] Saatchi M, Schnabel RD, Taylor JF, Garrick DJ (2014). Large-effect pleiotropic or closely linked QTL segregate within and across ten US cattle breeds. BMC Genomics.

[50] Snelling W, Allan M, Keele J, Kuehn L, Thallman R, Bennett G, Ferrell C, Jenkins T, Freetly H, Nielsen M (2011). Partial-genome evaluation of postweaning feed intake and efficiency of crossbred beef cattle. J Anim Sci.

[51] Lindholm-Perry AK, Kuehn LA, Oliver WT, Sexten AK, Miles JR, Rempel LA, Cushman RA, Freetly HC (2013). Adipose and muscle tissue gene expression of two genes (NCAPG and LCORL) located in a chromosomal region associated with cattle feed intake and gain. PLoS One.

[52] Setoguchi K, Furuta M, Hirano T, Nagao T, Watanabe T, Sugimoto Y, Takasuga A (2009). Cross-breed comparisons identified a critical 591-kb region for bovine carcass weight QTL (CW-2) on chromosome 6 and the Ile-442-met substitution in *NCAPG* as a positional candidate. BMC Genet.

[53] Juma AR, Damdimopoulou PE, Grommen SV, Wj VDV, De GB (2015). Emerging role of *PLAG1* as a regulator of growth and reproduction. J Endocrinol.

[54] Manning KS, Cooper TA (2017). The roles of RNA processing in translating genotype to phenotype. Nat Rev Mol Cell Biol.

[55] Kita Y, Mimori K, Iwatsuki M, Yokobori T, Ieta K, Tanaka F, Ishii H, Okumura H, Natsugoe S, Mori M (2011). STC2: a predictive marker for lymph node metastasis in esophageal squamous-cell carcinoma. Ann Surg Oncol.

[56] Chang AC, Hook J, Lemckert FA, McDonald MM, Nguyen MA, Hardeman EC, Little DG, Gunning PW, Reddel RR (2008). The murine stanniocalcin 2 gene is a negative regulator of postnatal growth. Endocrinology.

[57] Gagliardi AD, Kuo EY, Raulic S, Wagner GF, DiMattia GE (2005). Human stanniocalcin-2 exhibits potent growth-suppressive properties in transgenic mice independently of growth hormone and IGFs. Am J Physiol Endocrinol Metab.

[58] Sharma NK, Das SK, Mondal AK, Hackney OG, Chu WS, Kern PA, Rasouli N, Spencer HJ, Yao-Borengasser A, Elbein SC (2008). Endoplasmic reticulum stress markers are associated with obesity in nondiabetic subjects. J Clin Endocrinol Metab.

[59] Kazakova EV, Zghuang TW, Li TT, Fang QX, Han J, Qiao H (2017). The Gas6 gene rs8191974 and Ap3s2 gene rs2028299 are associated with type 2 diabetes in the northern Chinese Han population. Acta Biochim Pol.

[60] Conesa A, Madrigal P, Tarazona S, Gomez-Cabrero D, Cervera A, McPherson A, Szczesniak MW, Gaffney DJ, Elo LL, Zhang X (2016). A survey of best practices for RNA-seq data analysis. Genome Biol.

[61] Han Y, Gao S, Muegge K, Zhang W, Zhou B (2015). Advanced applications of RNA sequencing and challenges. Bioinform Biol Insights.

[62] Foote AP, Keel BN, Zarek CM, Lindholm-Perry AK (2017). Beef steers with average dry matter intake and divergent average daily gain have altered gene expression in the jejunum. J Anim Sci.

[63] Alexandre PA, Kogelman LJ, Santana MH, Passarelli D, Pulz LH, Fantinato-Neto P, Silva PL, Leme PR, Strefezzi RF, Coutinho LL (2015). Liver transcriptomic networks reveal main biological processes associated with feed efficiency in beef cattle. BMC Genomics.

[64] Chen L, Ekine-Dzivenu C, Vinsky M, Basarab J, Aalhus J, Dugan ME, Fitzsimmons C, Stothard P, Li C (2015). Genome-wide association and genomic prediction of breeding values for fatty acid composition in subcutaneous adipose and longissimus lumborum muscle of beef cattle. BMC Genet.

[65] Mukiibi R, Vinsky M, Keogh KA, Fitzsimmons C, Stothard P, Waters SM, Li C (2018). Transcriptome analyses reveal reduced hepatic lipid synthesis and accumulation in more feed efficient beef cattle. Sci Rep.

[66] McDonald P, Edwards R, Greenhalgh J, Morgan C (1988). Animal nutrition.

[67] Tizioto PC, Coutinho LL, Oliveira PS, Cesar AS, Diniz WJ, Lima AO, Rocha MI, Decker JE, Schnabel RD, Mourão GB (2016). Gene expression differences in Longissimus muscle of Nelore steers genetically divergent for residual feed intake. Sci Rep.

[68] Basarab JA, Colazo MG, Ambrose DJ, Novak S, McCartney D, Baron VS (2011). Residual feed intake adjusted for backfat thickness and feeding frequency is independent of fertility in beef heifers. Can J Anim Sci.

[69] Lu D, Miller S, Sargolzaei M, Kelly M, Vander Voort G, Caldwell T, Wang Z, Plastow G, Moore S (2013). Genome-wide association analyses for growth and feed efficiency traits in beef cattle. J Anim Sci.

[70] Browning BL, Browning SR (2016). Genotype imputation with millions of reference samples. Am J Hum Genet.

[71] Alexander DH, Novembre J, Lange K (2009). Fast model-based estimation of ancestry in unrelated individuals. Genome Res.

[72] Sargolzaei M, Chesnais JP, Schenkel FS (2014). A new approach for efficient genotype imputation using information from relatives. BMC Genomics.

[73] Daetwyler HD, Capitan A, Pausch H, Stothard P, Van Binsbergen R, Brondum RF, Liao XP, Djari A, Rodriguez SC, Grohs C (2014). Whole-genome sequencing of 234 bulls facilitates mapping of monogenic and complex traits in cattle. Nat Genet.

[74] Stothard P, Liao X, Arantes AS, De Pauw M, Coros C, Plastow GS, Sargolzaei M, Crowley JJ, Basarab JA, Schenkel F (2015). A large and diverse collection of bovine genome sequences from the Canadian cattle genome project. Gigascience.

[75] Yang J, Lee SH, Goddard ME, Visscher PM (2011). GCTA: a tool for genome-wide complex trait analysis. Am J Hum Genet.

[76] Yang J, Zaitlen NA, Goddard ME, Visscher PM, Price AL (2014). Advantages and pitfalls in the application of mixed-model association methods. Nat Genet.

[77] Yang J, Benyamin B, McEvoy BP, Gordon S, Henders AK, Nyholt DR, Madden PA, Heath AC, Martin NG, Montgomery GW (2010). Common SNPs explain a large proportion of the heritability for human height. Nat Genet.

[78] Lee SH, Wray NR, Goddard ME, Visscher PM (2011). Estimating missing heritability for disease from genome-wide association studies. Am J Hum Genet.

[79] Yang J, Manolio TA, Pasquale LR, Boerwinkle E, Caporaso N, Cunningham JM, Andrade M, Feenstra B, Feingold E, Hayes MG (2011). Genome partitioning of genetic variation for complex traits using common SNPs. Nat Genet.

[80] Benjamin DJ, Berger JO, Johannesson M, Nosek BA, Wagenmakers E-J, Berk R, Bollen KA, Brembs B, Brown L, Camerer C (2018). Redefine statistical significance. Nat Hum Behav.

[81] Wellcome Trust Case Control C (2007). Genome-wide association study of 14,000 cases of seven common diseases and 3,000 shared controls. Nature.

[82] Yang J, Bakshi A, Zhu Z, Hemani G, Vinkhuyzen AA, Lee SH, Robinson MR, Perry JR, Nolte IM, van Vliet-Ostaptchouk JV (2015). Genetic variance estimation with imputed variants finds negligible missing heritability for human height and body mass index. Nat Genet.

[83] Yang J, Zeng J, Goddard ME, Wray NR, Visscher PM (2017). Concepts, estimation and interpretation of SNP-based heritability. Nat Genet.

[84] Grant JR, Arantes AS, Liao XP, Stothard P (2011). In-depth annotation of SNPs arising from resequencing projects using NGS-SNP. Bioinformatics.

