# Supplementary material for: Genetic architecture of quantitative traits in beef cattle revealed by genome wide association studies of imputed whole genome sequence variants: I: feed efficiency and component traits
Source: BMC Genomics. 2020 Jan 13;21:36. doi: 10.1186/s12864-019-6362-1 (PMC6956504; doi:10.1186/s12864-019-6362-1)
Supplement: Supplementary file 1 — Additional file 1: Figure S1. Distribution of SNP allele substitution effects (left) and additive genetic variances explained by single SNPs (right) for RFI, DMI, ADG, and MWT based on 7.8 M whole genome sequence (WGS) variants; Figure S2. The enriched cellular and molecular functions (top) and cell morphology network (bottom) for RFI; Figure S3. The enriched cellular and molecular functions (top) and carbohydrate metabolism network (bottom) for DMI; Figure S4. The enriched cellular and molecular functions (top) and carbohydrate metabolism network (bottom) for ADG; Figure S5. The enriched cellular and molecular functions (top) and cellular compromise network (bottom) for MWT; Figure S6. Lipid metabolism network for RFI and its component traits DMI, ADG, and MWT; Figure S7. Common lead significant SNPs with the same IDs (a) and candidate genes (b) among RFI and its three component traits at thresholds P-value < 1.00E-05 and FDR < 0.10 based on the imputed 7.8 M DNA variant GWAS; Figure S8. Principal component analyses of Canadian beef cattle populations (N = 7573) based on the 50 K (a) and 7.8 M WGS (b) panels. [file 12864_2019_6362_MOESM1_ESM.pdf]

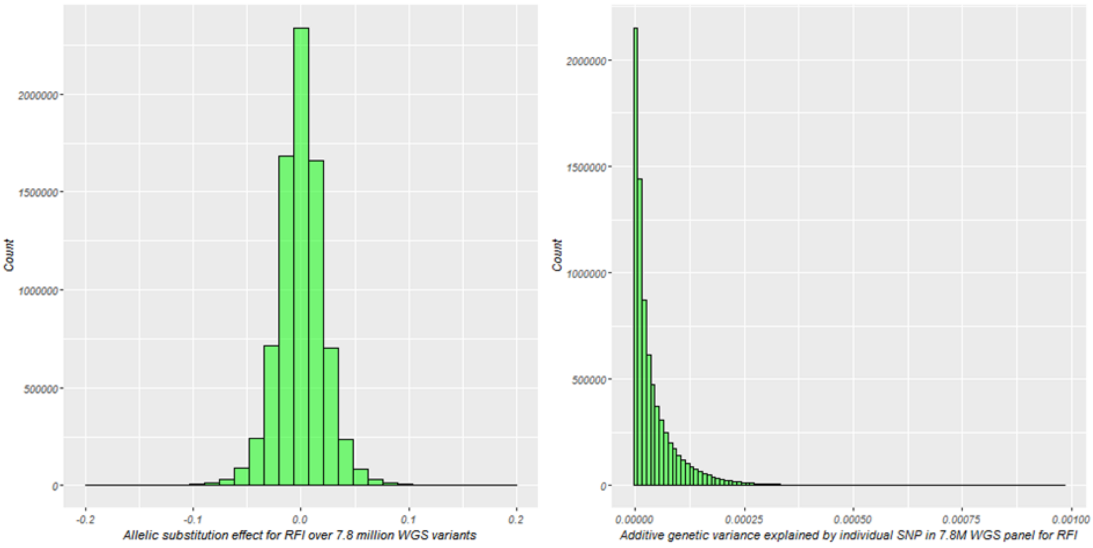

RFI_7.8M GWAS

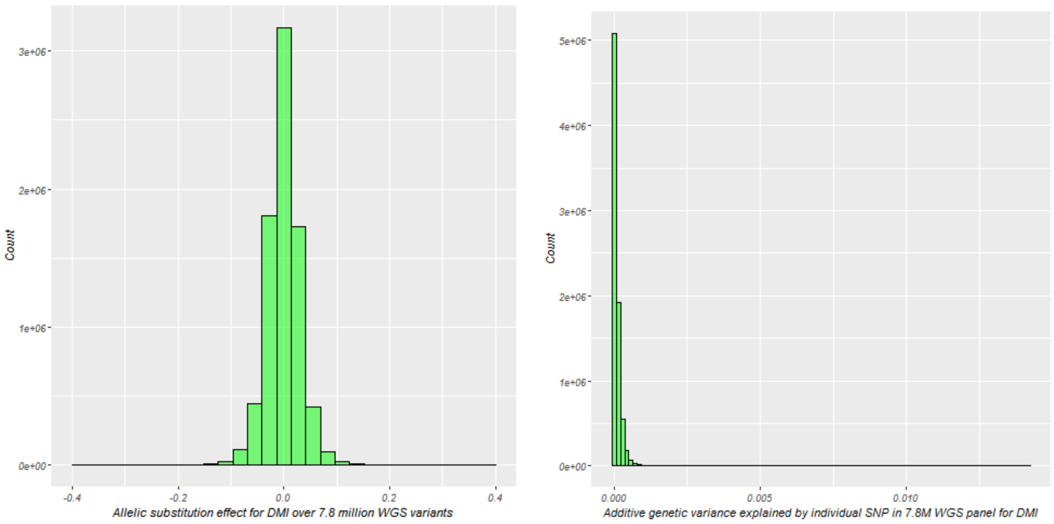

DMI_7.8M GWAS

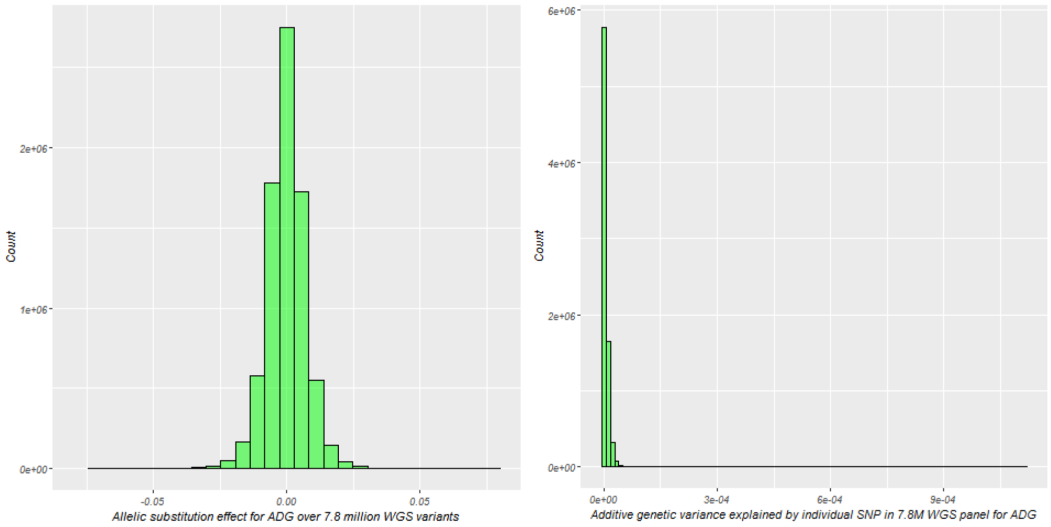

ADG_7.8M GWAS

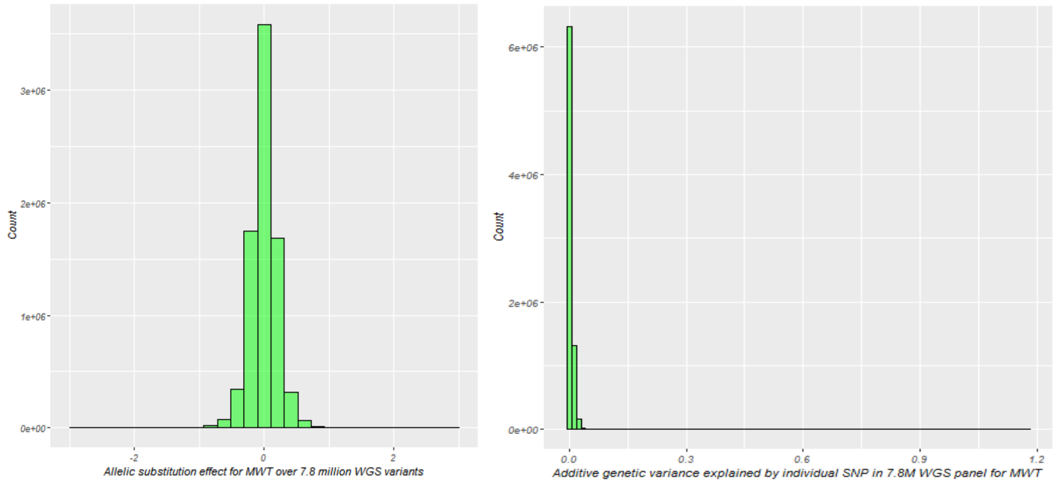

MWT_7.8M GWAS

Figure S1. Distribution of SNP allele substitution effects (left) and additive genetic variances explained by single SNPs (right) for RFI, DMI, ADG, and MWT based on 7.8 M whole genome sequence (WGS) variants.

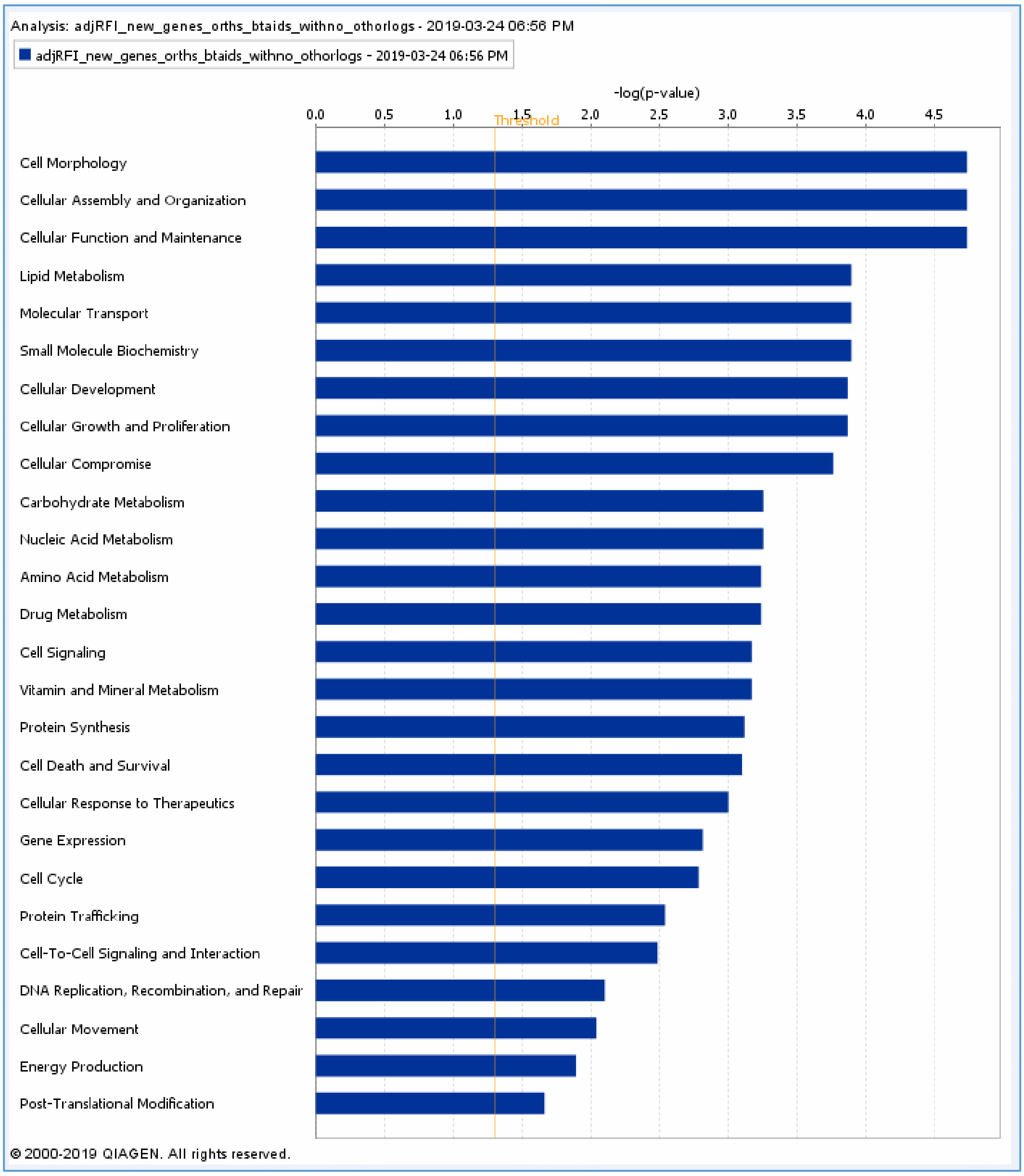
Analysis: adjRFI_new_genes_orths_btaids_withno_othorlogs - 2019-03-24 06:56 PM
adjRFI_new_genes_orths_btaids_withno_othorlogs - 2019-03-24 06:56 PM
-log(p-value)
0.0
0.5
1.0
1.5
2.0
2.5
3.0
3.5
4.0
4.5
Threshold
Cell Morphology
Cellular Assembly and Organization
Cellular Function and Maintenance
Lipid Metabolism
Molecular Transport
Small Molecule Biochemistry
Cellular Development
Cellular Growth and Proliferation
Cellular Compromise
Carbohydrate Metabolism
Nucleic Acid Metabolism
Amino Acid Metabolism
Drug Metabolism
Cell Signaling
Vitamin and Mineral Metabolism
Protein Synthesis
Cell Death and Survival
Cellular Response to Therapeutics
Gene Expression
Cell Cycle
Protein Trafficking
Cell-To-Cell Signaling and Interaction
DNA Replication, Recombination, and Repair
Cellular Movement
Energy Production
Post-Translational Modification
© 2000-2019 QIAGEN. All rights reserved.

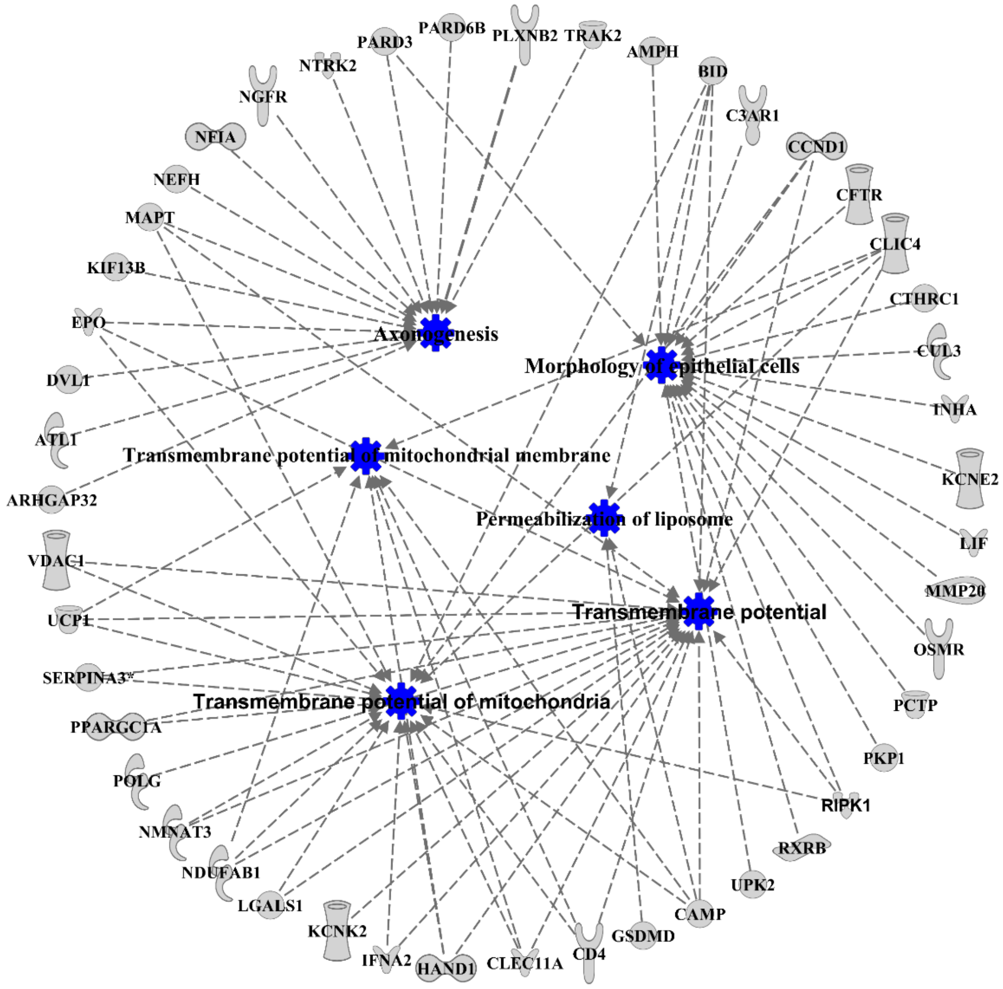

© 2000-2019 QIAGEN. All rights reserved.

Figure S2. The enriched cellular and molecular functions (top) and cell morphology network (bottom) for RFI. The images were generated through the use of Ingenuity Pathway Analysis (IPA) and written permission was granted by QIAGEN Silicon Valley to use and adapt the figure generated by IPA under the terms of the Creative Commons Attribution License (CC BY) 4.0.

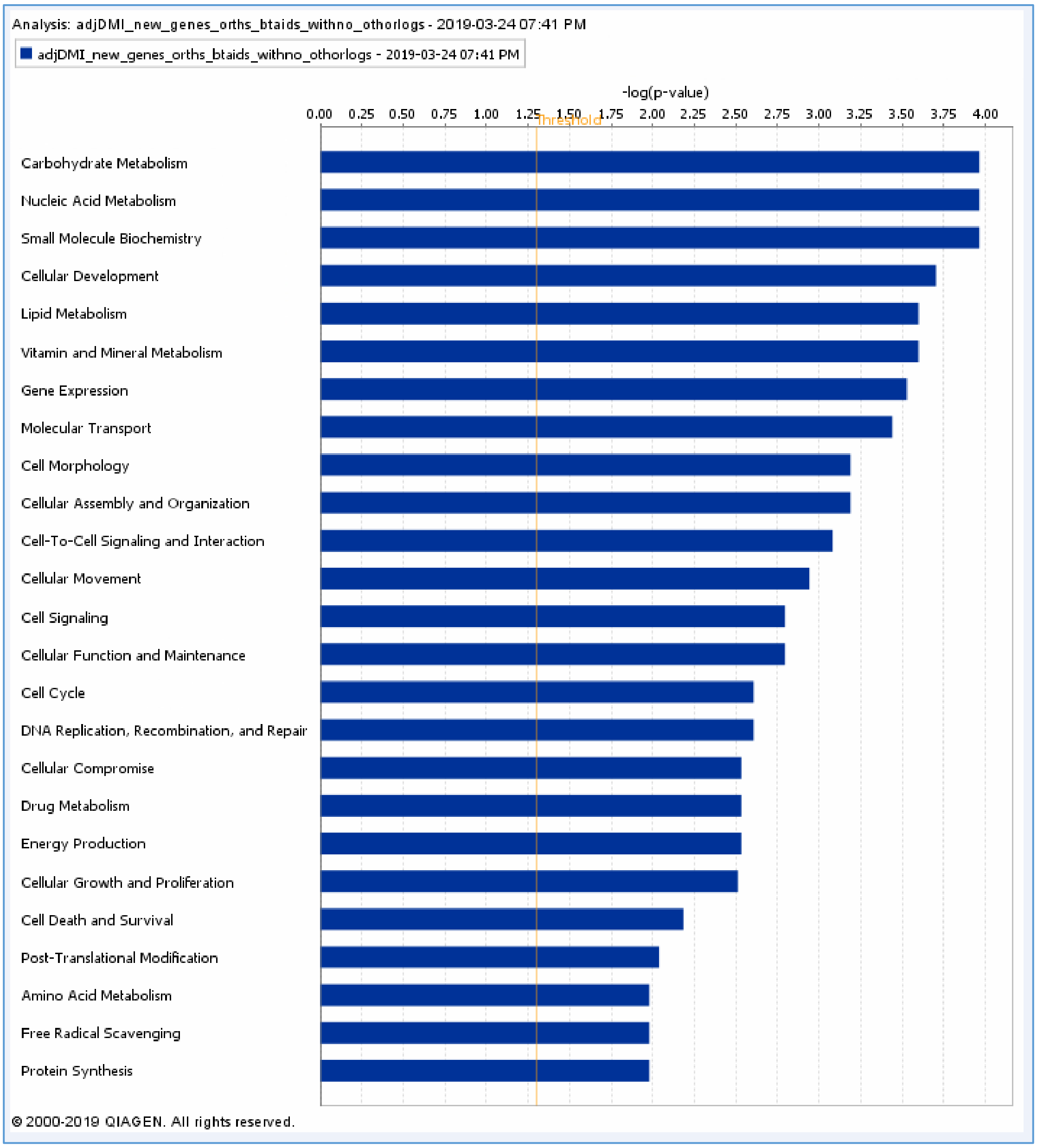
Analysis: adjDMI_new_genes_orths_btaids_withno_othorlogs - 2019-03-24 07:41 PM
adjDMI_new_genes_orths_btaids_withno_othorlogs - 2019-03-24 07:41 PM
-log(p-value)
0.00 0.25 0.50 0.75 1.00 1.25 1.50 1.75 2.00 2.25 2.50 2.75 3.00 3.25 3.50 3.75 4.00
Threshold
Carbohydrate Metabolism
Nucleic Acid Metabolism
Small Molecule Biochemistry
Cellular Development
Lipid Metabolism
Vitamin and Mineral Metabolism
Gene Expression
Molecular Transport
Cell Morphology
Cellular Assembly and Organization
Cell-To-Cell Signaling and Interaction
Cellular Movement
Cell Signaling
Cellular Function and Maintenance
Cell Cycle
DNA Replication, Recombination, and Repair
Cellular Compromise
Drug Metabolism
Energy Production
Cellular Growth and Proliferation
Cell Death and Survival
Post-Translational Modification
Amino Acid Metabolism
Free Radical Scavenging
Protein Synthesis
© 2000-2019 QIAGEN. All rights reserved.

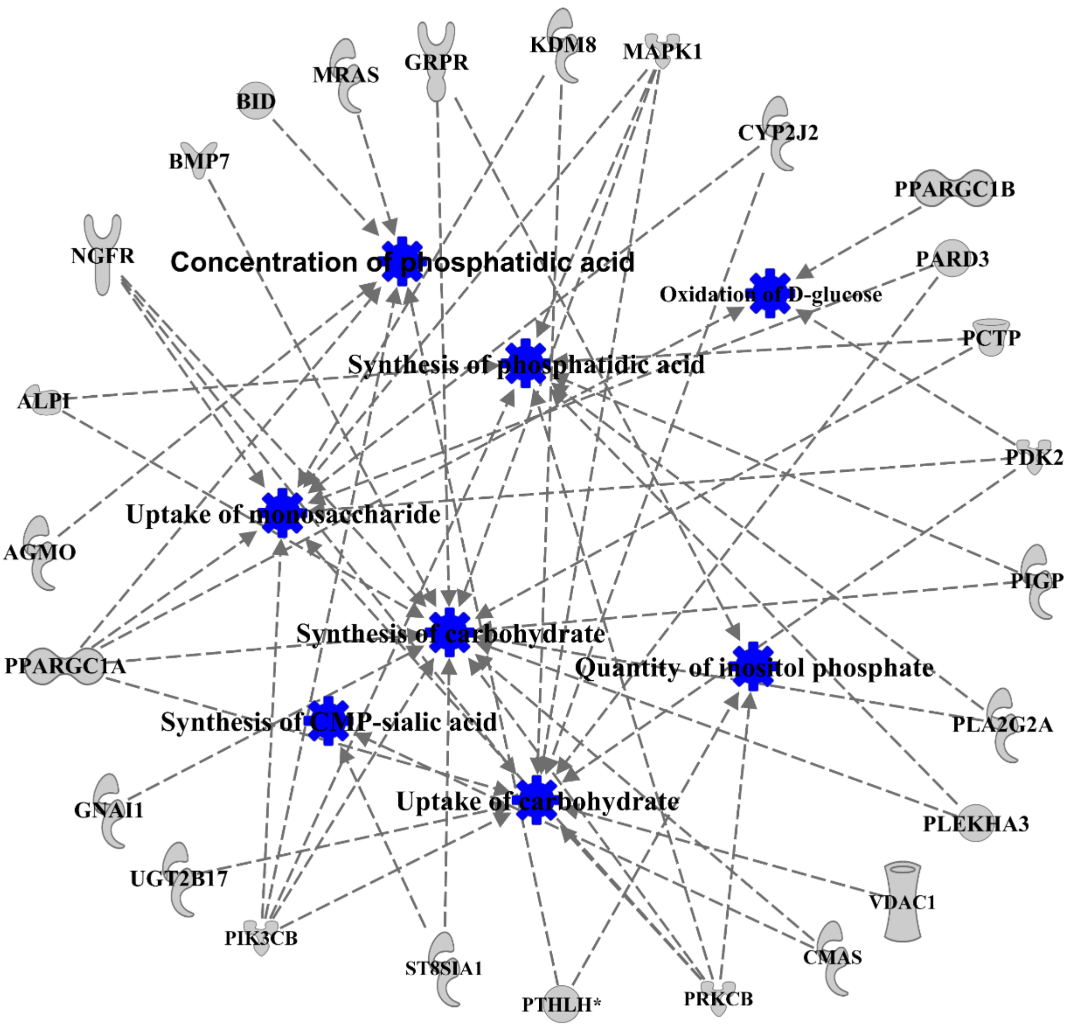

Figure S3. The enriched cellular and molecular functions (top) and carbohydrate metabolism network (bottom) for DMI. The images were generated through the use of Ingenuity Pathway Analysis (IPA) and written permission was granted by QIAGEN Silicon Valley to use and adapt the figure generated by IPA under the terms of the Creative Commons Attribution License (CC BY) 4.0.

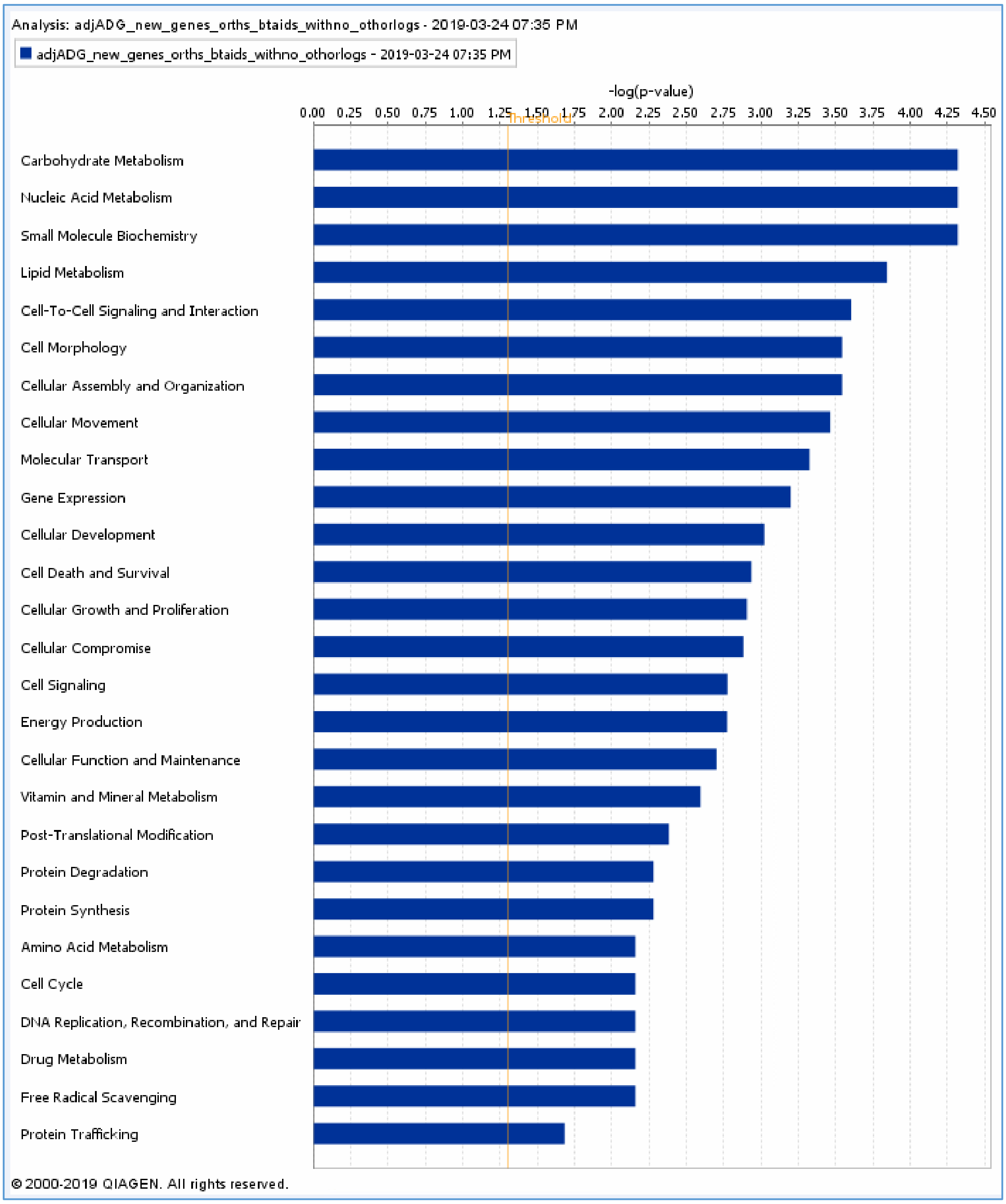
Analysis: adjADG_new_genes_orths_btaids_withno_othorlogs - 2019-03-24 07:35 PM
adjADG_new_genes_orths_btaids_withno_othorlogs - 2019-03-24 07:35 PM
-log(p-value)
0.00 0.25 0.50 0.75 1.00 1.25 1.50 1.75 2.00 2.25 2.50 2.75 3.00 3.25 3.50 3.75 4.00 4.25 4.50
Threshold
Carbohydrate Metabolism
Nucleic Acid Metabolism
Small Molecule Biochemistry
Lipid Metabolism
Cell-To-Cell Signaling and Interaction
Cell Morphology
Cellular Assembly and Organization
Cellular Movement
Molecular Transport
Gene Expression
Cellular Development
Cell Death and Survival
Cellular Growth and Proliferation
Cellular Compromise
Cell Signaling
Energy Production
Cellular Function and Maintenance
Vitamin and Mineral Metabolism
Post-Translational Modification
Protein Degradation
Protein Synthesis
Amino Acid Metabolism
Cell Cycle
DNA Replication, Recombination, and Repair
Drug Metabolism
Free Radical Scavenging
Protein Trafficking
© 2000-2019 QIAGEN. All rights reserved.

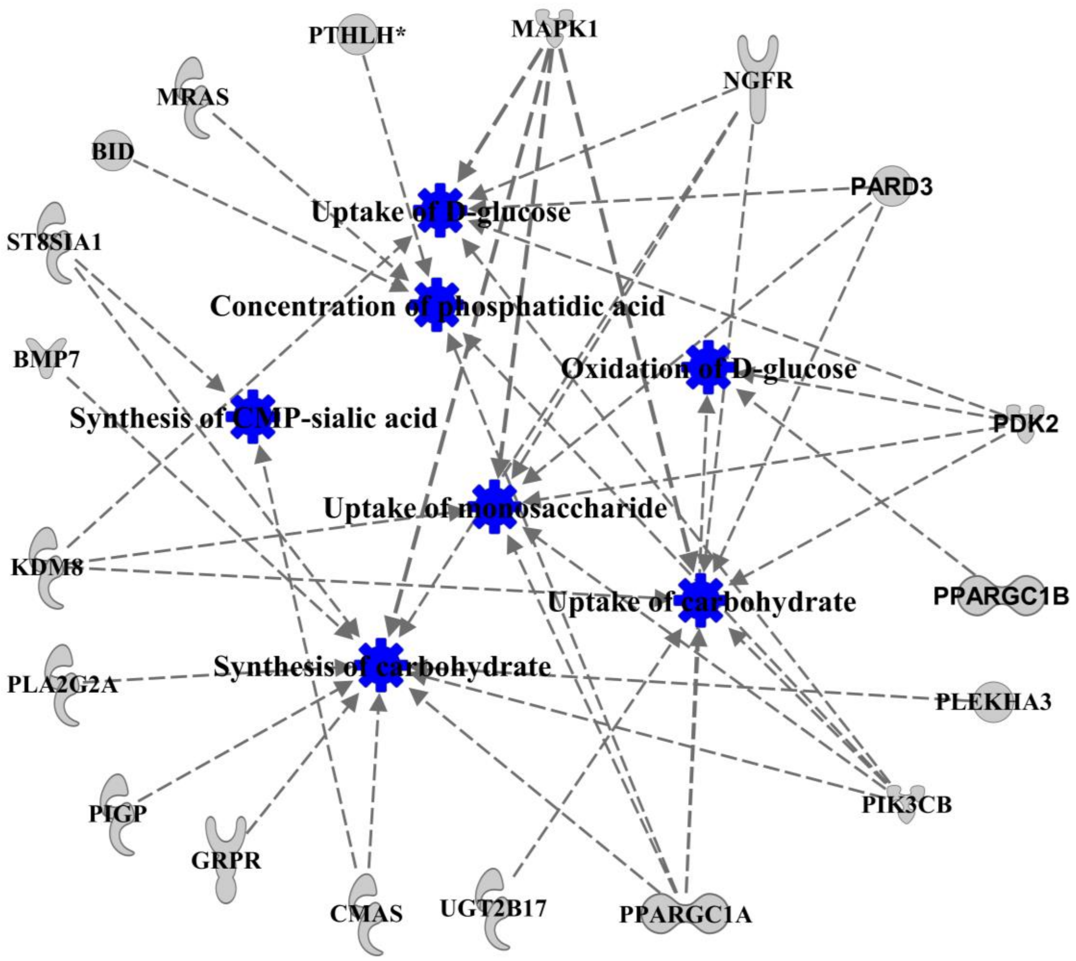

Figure S4. The enriched cellular and molecular functions (top) and carbohydrate metabolism network (bottom) for ADG. The images were generated through the use of Ingenuity Pathway Analysis (IPA) and written permission was granted by QIAGEN Silicon Valley to use and adapt the figure generated by IPA under the terms of the Creative Commons Attribution License (CC BY) 4.0.

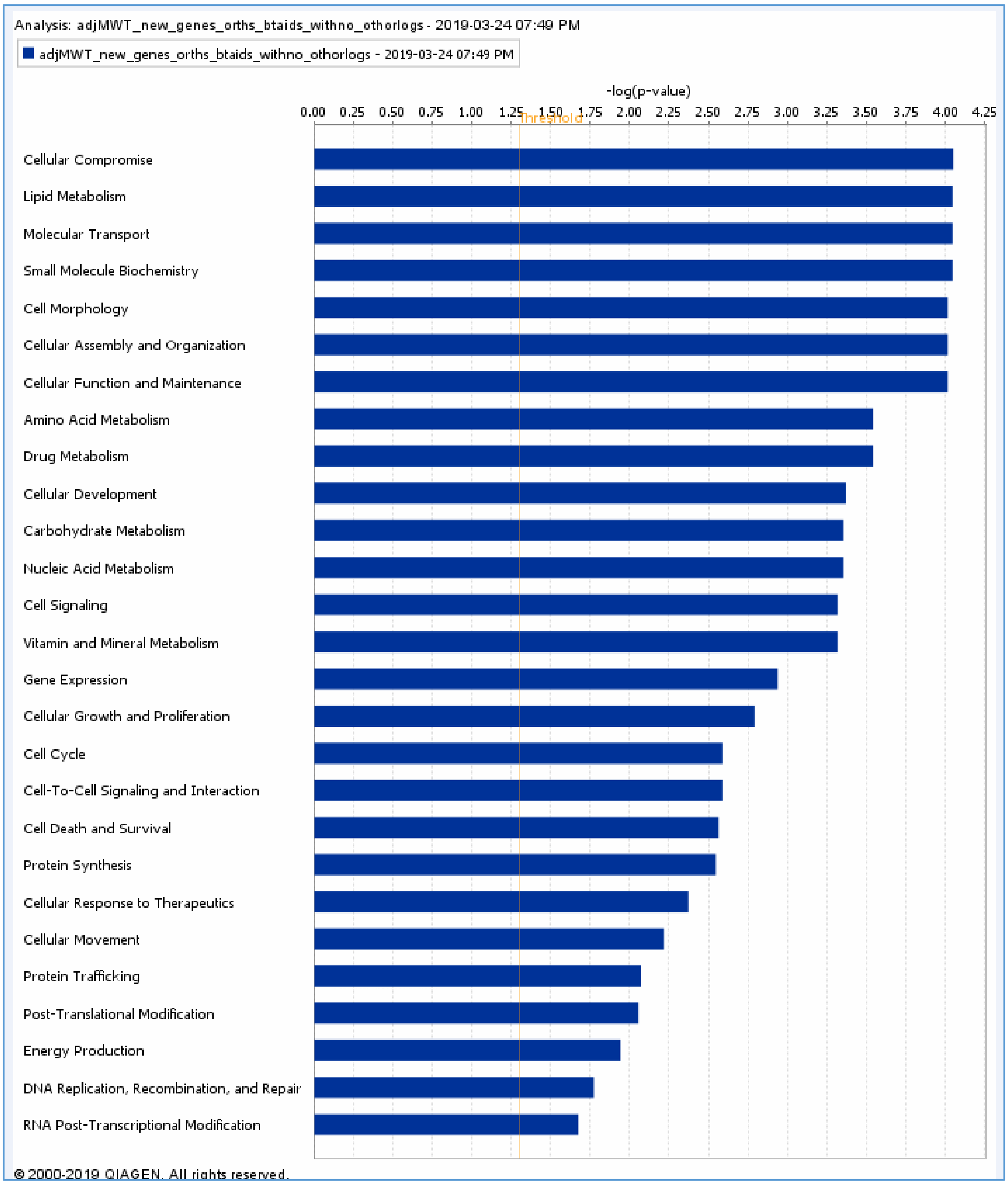
Analysis: adjMWT_new_genes_orths_btaids_withno_othorlogs - 2019-03-24 07:49 PM
adjMWT_new_genes_orths_btaids_withno_othorlogs - 2019-03-24 07:49 PM
-log(p-value)
0.00 0.25 0.50 0.75 1.00 1.25 1.50 1.75 2.00 2.25 2.50 2.75 3.00 3.25 3.50 3.75 4.00 4.25
Threshold
Cellular Compromise
Lipid Metabolism
Molecular Transport
Small Molecule Biochemistry
Cell Morphology
Cellular Assembly and Organization
Cellular Function and Maintenance
Amino Acid Metabolism
Drug Metabolism
Cellular Development
Carbohydrate Metabolism
Nucleic Acid Metabolism
Cell Signaling
Vitamin and Mineral Metabolism
Gene Expression
Cellular Growth and Proliferation
Cell Cycle
Cell-To-Cell Signaling and Interaction
Cell Death and Survival
Protein Synthesis
Cellular Response to Therapeutics
Cellular Movement
Protein Trafficking
Post-Translational Modification
Energy Production
DNA Replication, Recombination, and Repair
RNA Post-Transcriptional Modification
© 2000-2019 QIAGEN. All rights reserved.

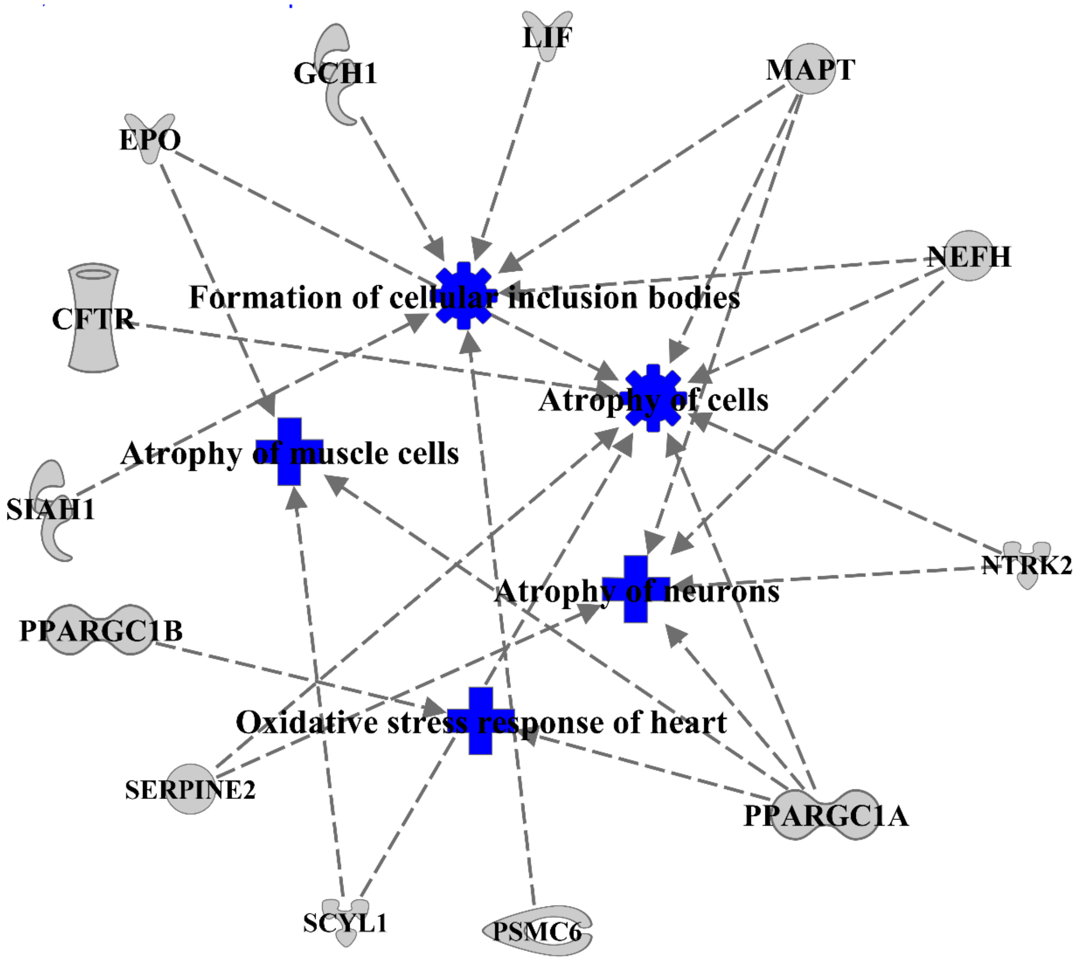

Figure S5. The enriched cellular and molecular functions (top) and cellular compromise network (bottom) for MWT. The images were generated through the use of Ingenuity Pathway Analysis (IPA) and written permission was granted by QIAGEN Silicon Valley to use and adapt the figure generated by IPA under the terms of the Creative Commons Attribution License (CC BY) 4.0.

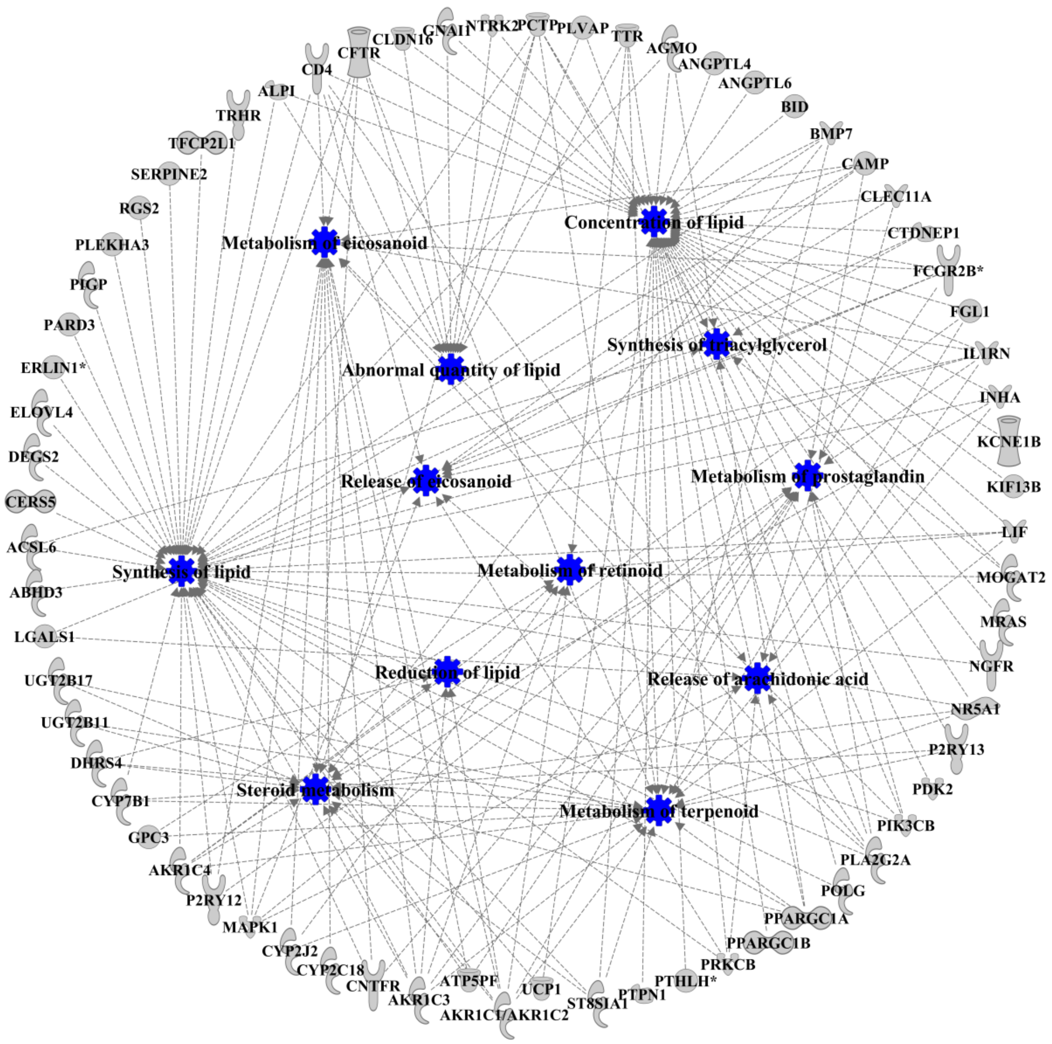

Concentration of lipid
Metabolism of eicosanoid
Abnormal quantity of lipid
Synthesis of triacylglycerol
Release of eicosanoid
Metabolism of prostaglandin
Synthesis of lipid
Metabolism of retinoid
Reduction of lipid
Release of arachidonic acid
Steroid metabolism
Metabolism of terpenoid
GNAI1
NTRK2
PCTP
PLVAP
TTR
CLDN16
CFTR
AGMO
ANGPTL4
ANGPTL6
CD4
ALPI
BID
TRHR
BMP7
TFCP2L1
CAMP
SERPINE2
CLEC11A
RGS2
CTDNEP1
PLEKHA3
FCGR2B*
PIGP
FGL1
PARD3
IL1RN
ERLIN1*
INHA
ELOVL4
KCNE1B
DEGS2
KIF13B
CERS5
LIF
ACSL6
MOGAT2
ABHD3
MRAS
LGALS1
NGFR
UGT2B17
NR5A1
UGT2B11
P2RY13
DHRS4
PDK2
CYP7B1
PIK3CB
GPC3
PLA2G2A
AKR1C4
POLG
P2RY12
PPARGC1A
MAPK1
PPARGC1B
CYP2J2
PRKCB
CYP2C18
PTHLH*
CNTFR
ATP5PF
UCP1
PTPN1
AKR1C3
ST8SIA1
AKR1C1/AKR1C2

RFI

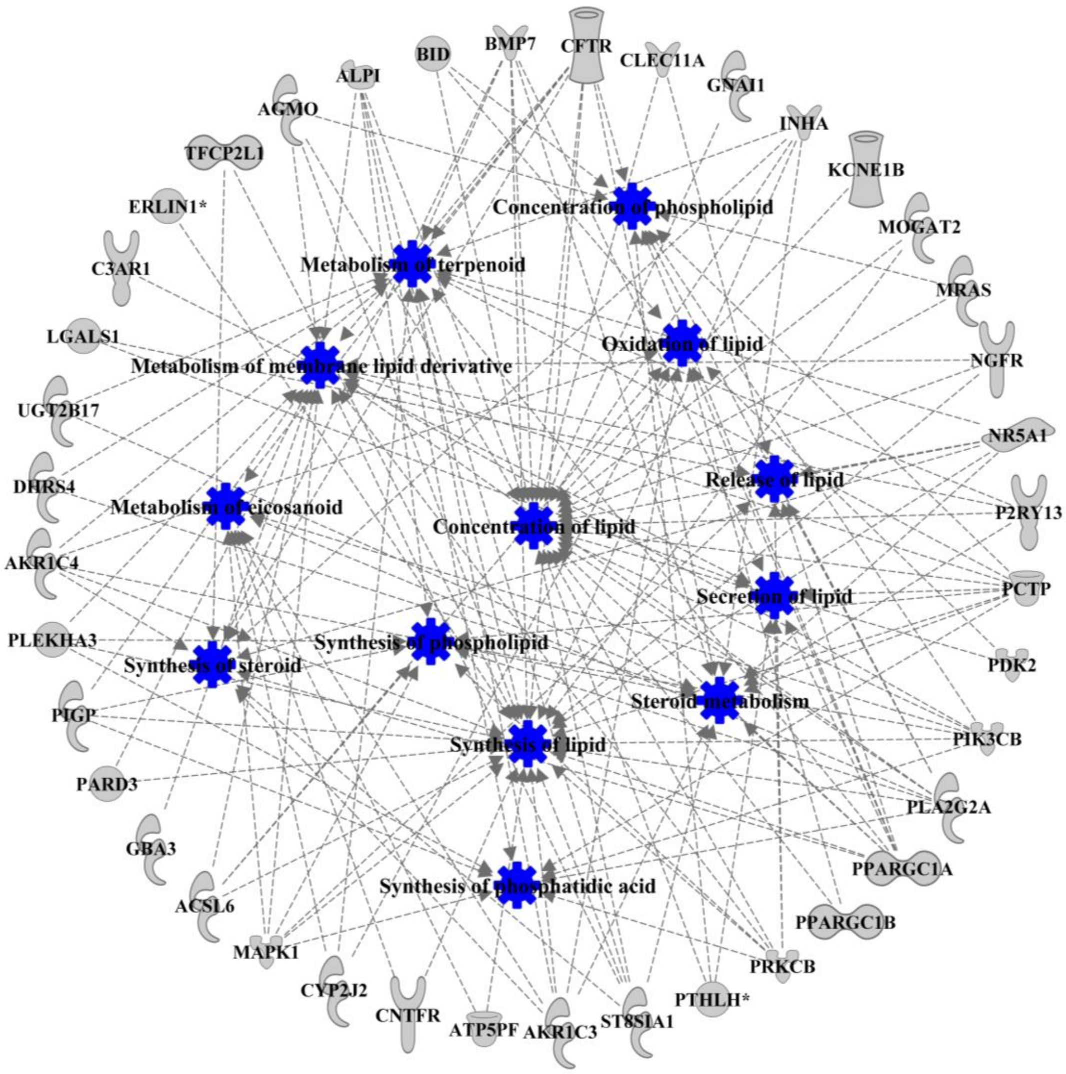
BID
BMP7
CFTR
CLEC11A
GNAI1
ALPI
AGMO
INHA
TFCP2L1
KCNE1B
ERLIN1*
Concentration of phospholipid
MOGAT2
C3AR1
Metabolism of terpenoid
MRAS
LGALS1
Oxidation of lipid
NGFR
Metabolism of membrane lipid derivative
UGT2B17
NR5A1
DHRS4
Release of lipid
Metabolism of eicosanoid
P2RY13
Concentration of lipid
AKR1C4
PCTP
Secretion of lipid
PLEKHA3
Synthesis of phospholipid
PDK2
Synthesis of steroid
Steroid metabolism
PIGP
PIK3CB
Synthesis of lipid
PARD3
PLA2G2A
GBA3
PPARGC1A
Synthesis of phosphatidic acid
ACSL6
PPARGC1B
MAPK1
PRKCB
CYP2J2
PTHLH*
CNTFR
ATP5PF
AKR1C3
ST8SIA1

DMI

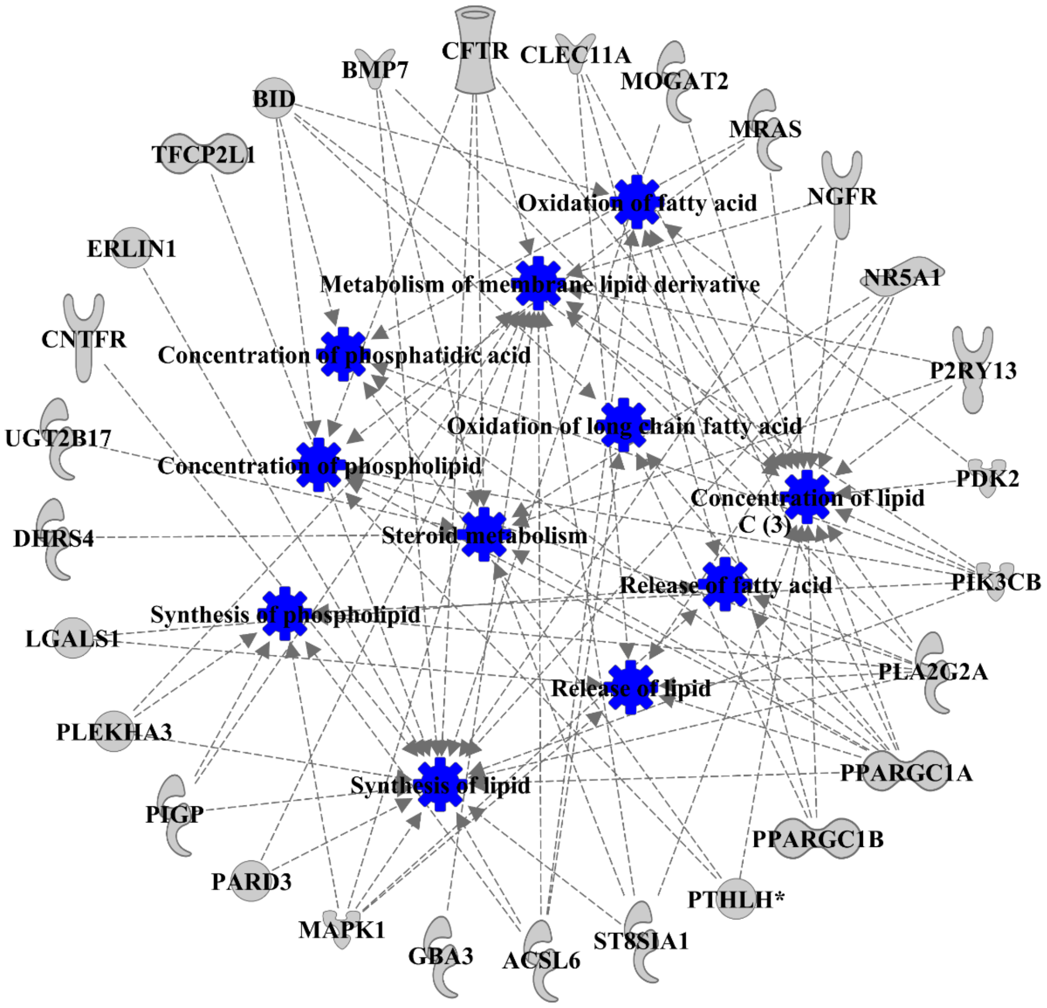
CFTR
CLEC11A
BMP7
MOGAT2
BID
MRAS
TFCP2L1
Oxidation of fatty acid
NGFR
ERLIN1
Metabolism of membrane lipid derivative
NR5A1
CNTFR
Concentration of phosphatidic acid
P2RY13
UGT2B17
Oxidation of long chain fatty acid
Concentration of phospholipid
PDK2
Concentration of lipid
C (3)
DHRS4
Steroid metabolism
Release of fatty acid
PIK3CB
Synthesis of phospholipid
LGALS1
PLA2G2A
Release of lipid
PLEKHA3
PPARGC1A
Synthesis of lipid
PIGP
PPARGC1B
PARD3
PTHLH*
MAPK1
ST8SIA1
GBA3
ACSL6

ADG

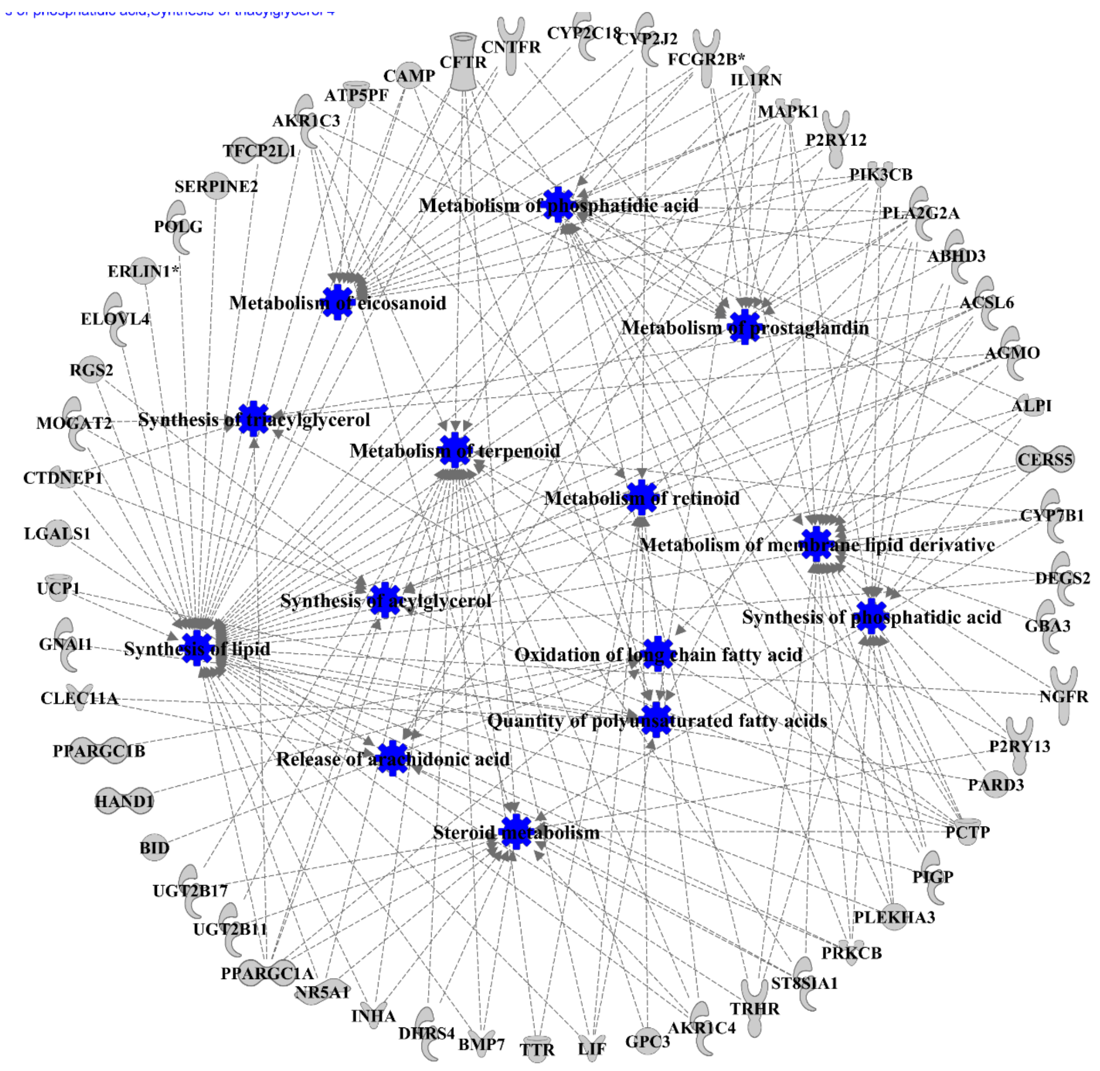

MWT

Figure S6. Lipid metabolism network for RFI and its component traits DMI, ADG, and MWT. The images were generated through the use of Ingenuity Pathway Analysis (IPA) and written permission was granted by QIAGEN Silicon Valley to use and adapt the figure generated by IPA under the terms of the Creative Commons Attribution License (CC BY) 4.0.

a

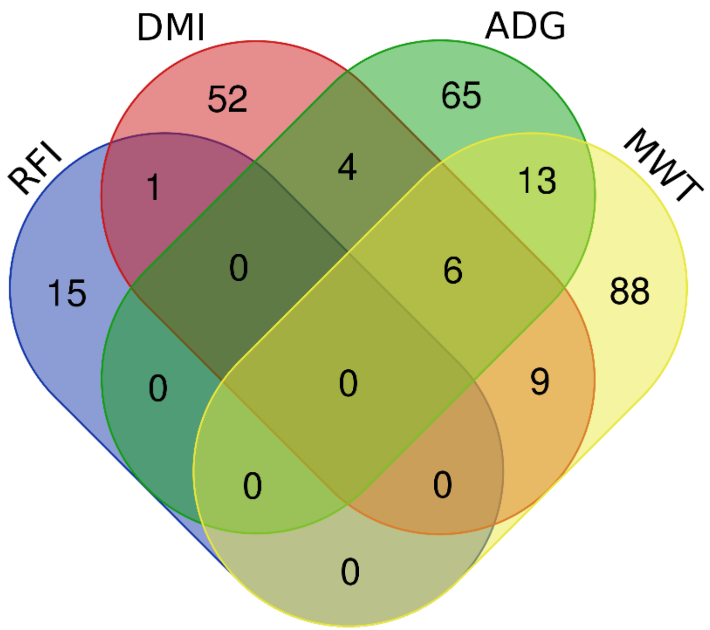

b

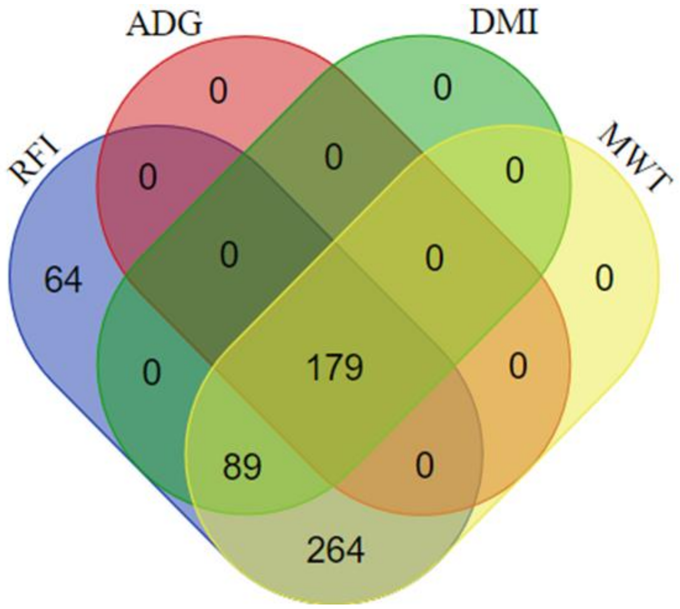

Figure S7. Common lead significant SNPs with the same IDs (a) and candidate genes (b) among RFI and its three component traits at thresholds P-value < 1.00E-05 and FDR < 0.10 based on the imputed 7.8M DNA variant GWAS.

a

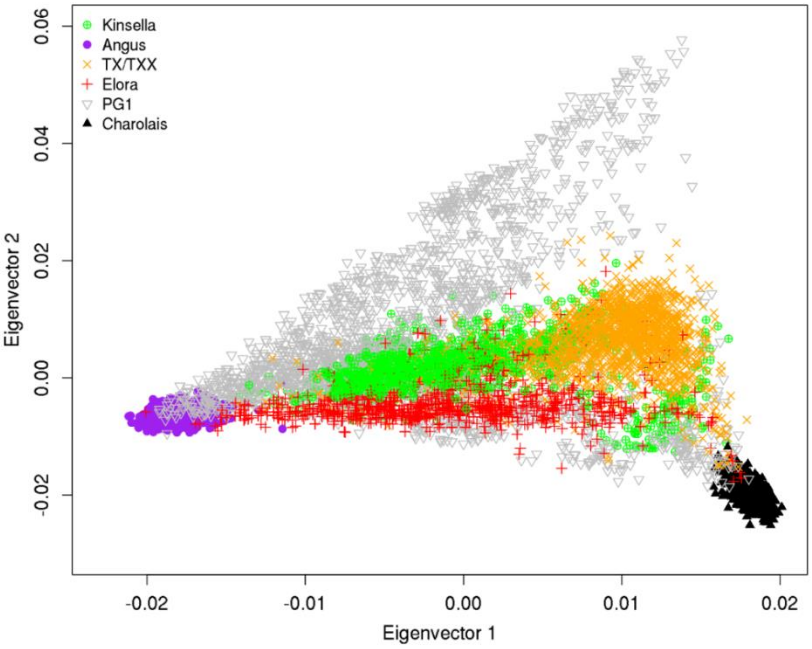

b

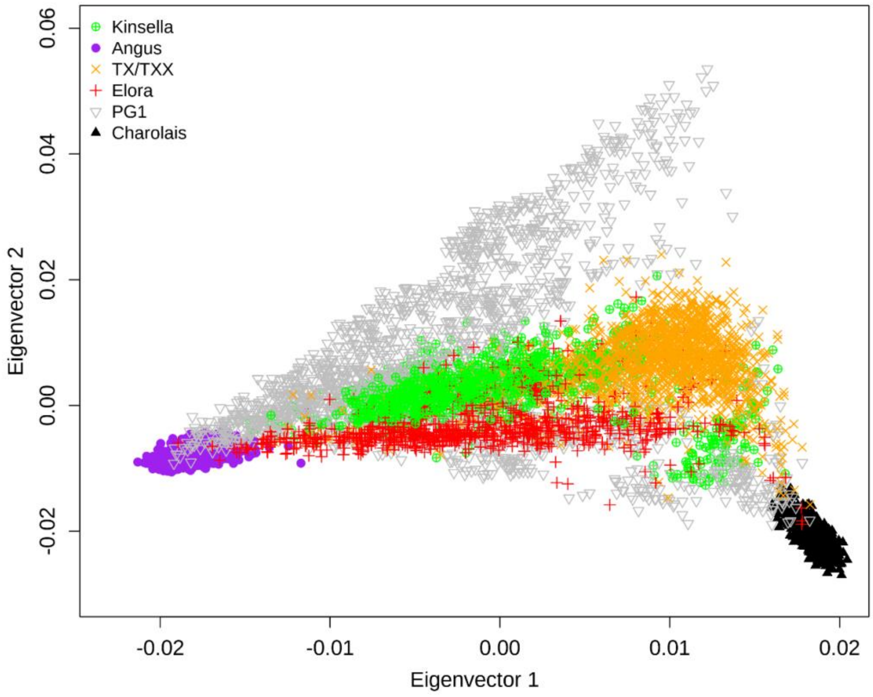

Figure S8. Principal component analyses of Canadian beef cattle populations (N=7573) based on the 50K SNP (a) and 7.8M GWS variant (b) panels.
