# Supplementary material for: Genetic architecture of quantitative traits in beef cattle revealed by genome wide association studies of imputed whole genome sequence variants: I: feed efficiency and component traits
Source: BMC Genomics. 2020 Jan 13;21:36. doi: 10.1186/s12864-019-6362-1 (PMC6956504; doi:10.1186/s12864-019-6362-1)
Supplement: Supplementary file 3 — Additional file 3: Table S1. Accuracy of genotype imputation from Illumina 50 K SNPs to whole genome sequence (WGS) variant genotypes via HD using Fimpute 2.2; Table S2. Functional annotations of 7.8 M WGS variants along with the number of variants in each class, classification of SNP functions, percentage of WGS and 9 functional class assignments; Table S3. Functional annotation of all DNA variants (38,318,974) based on DNA variants of the 1000 bulls genome project; Table S4. Functional annotations of SNPs in the 50 K SNP panel after quality control along with the number of variants in each class, classification of SNP functions, percentage of WGS, and 9 functional class assignments; Table S5. List of lead SNPs that were overlapped with QTLs published in Cattle QTL database for RFI, DMI, ADG, and MWT. [file 12864_2019_6362_MOESM3_ESM.pdf]

Table S1. Accuracy of genotype imputation from Illumina 50K SNPs to whole genome sequence (WGS) variant genotypes via HD using Fimpute 2.2

	Number of total WGS variant genotypes of 240 animals	Number of correctly imputed WGS variant genotypes of 240 animals	Imputation accuracy	Number of imputed WGS variant genotypes per animal
BTA1	579745680	562381176	0.97	2415607
BTA2	494930160	480738782	0.9713	2062209
BTA3	419358240	405858001	0.9678	1747326
BTA4	441735600	427070290	0.9668	1840565
BTA5	429833520	413270103	0.9615	1790973
BTA6	425628240	412122198	0.9683	1773451
BTA7	386629200	373029804	0.9648	1610955
BTA8	389297760	377097399	0.9687	1622074
BTA9	373340160	361456820	0.9682	1555584
BTA10	367824960	353656505	0.9615	1532604
BTA11	371232240	359010340	0.9671	1546801
BTA12	400109760	372585988	0.9312	1667124
BTA13	296662080	287426524	0.9689	1236092
BTA14	296392560	286832216	0.9677	1234969
BTA15	339638400	326968651	0.9627	1415160
BTA16	309841200	298947370	0.9648	1291005
BTA17	277840560	265963664	0.9573	1157669
BTA18	231474480	221864425	0.9585	964477
BTA19	223123920	213964332	0.9589	929683
BTA20	269202000	261302594	0.9707	1121675
BTA21	261251520	251574275	0.963	1088548
BTA22	214242720	207343689	0.9678	892678
BTA23	243928080	233011283	0.9552	1016367
BTA24	238661280	231785947	0.9712	994422
BTA25	160847520	155123815	0.9644	670198
BTA26	187047.12	180321128	0.964	779363
BTA27	167550240	161171728	0.9619	698126
BTA28	185485440	179423584	0.9673	772856
BTA29	213699120	205252771	0.9605	890413
Whole-genome	9,196,553,760	8,866,555,402	0.9641	38,318,974

Table S2. Functional annotations of 7.8M WGS variants along with the number of variants in each class, classification of SNP functions, percentage of WGS and 9 functional class assignments

Functional class annotation	Number of SNPs	Classification of SNP functions	Proportion*
intergenic_region	5251680	intergenic_region	66.87%
downstream_gene_variant	253163	downstream_gene_variant	3.22%
upstream_gene_variant	285798	upstream_gene_variant	3.64%
non_coding_exon_variant	1953	synonymous_variant	0.41%
synonymous_variant	30066	synonymous_variant	
intron_variant	1987366	intron_variant	25.31%
missense_variant	17654	missense_variant	0.22%
3’UTR_variant	15851	3’UTR_variant	0.20%
5’UTR_variant	2868	5’UTR_variant	0.04%
5’UTR_premature_start_codon_gain_variant	441	5’UTR_variant	
splice_region_variant&intron_variant	4189	Other_regulatory	0.08%
missense_variant&splice_region_variant	470	Other_regulatory	
splice_region_variant&synonymous_variant	664	Other_regulatory	
inframe_deletion	98	Other_regulatory	
frameshift_variant	150	Other_regulatory	
splice_donor_variant&intron_variant	118	Other_regulatory	
disruptive_inframe_deletion	52	Other_regulatory	
splice_region_variant	141	Other_regulatory	
splice_acceptor_variant&intron_variant	116	Other_regulatory	
frameshift_variant&splice_donor_variant&splice_region_variant&intron_variant	3	Other_regulatory	
splice_acceptor_variant&splice_donor_variant&intron_variant	19	Other_regulatory	
splice_region_variant&non_coding_exon_variant	61	Other_regulatory	
stop_retained_variant	19	Other_regulatory	

frameshift_variant&splice_region_variant	13	Other_regulatory	
stop_gained	161	Other_regulatory	
splice_acceptor_variant&splice_region_variant&intron_variant	11	Other_regulatory	
splice_region_variant&stop_retained_variant	3	Other_regulatory	
stop_lost&splice_region_variant	9	Other_regulatory	
start_lost	27	Other_regulatory	
disruptive_inframe_insertion	7	Other_regulatory	
inframe_insertion	13	Other_regulatory	
frameshift_variant&stop_lost&splice_region_variant	1	Other_regulatory	
stop_gained&splice_region_variant	9	Other_regulatory	
stop_lost	3	Other_regulatory	
frameshift_variant&splice_acceptor_variant&splice_region_variant&intron_variant	2	Other_regulatory	
frameshift_variant&stop_gained	1	Other_regulatory	
splice_donor_variant&splice_region_variant&intron_variant	5	Other_regulatory	
stop_gained&inframe_insertion	1	Other_regulatory	
initiator_codon_variant&non_canonical_start_codon	1	Other_regulatory	
frameshift_variant&start_lost&splice_region_variant	1	Other_regulatory	
frameshift_variant&stop_lost	1	Other_regulatory	
frameshift_variant&start_lost	1	Other_regulatory	
disruptive_inframe_deletion&splice_region_variant	1	Other_regulatory	
Total	7853211		100.00%

* The proportion was calculated as numbers of SNPs in a class divided by total SNPs/Indel and then multiplied by 100.

Table S3. Functional annotation of all DNA variants (38,318,974) based on DNA variants of the 1000 bulls genome project.

Functional class annotation	Number of SNPs	Classification of SNP functions	Proportion*
intergenic_region	25179655	intergenic_region	65.70%
downstream_gene_variant	1393115	downstream_gene_variant	3.60%
upstream_gene_variant	1591843	upstream_gene_variant	4.20%
non_coding_exon_variant	10973	synonymous_variant	0.43%
synonymous_variant	155200	synonymous_variant	
intron_variant	9693772	intron_variant	25.30%
missense_variant	143708	missense_variant	0.40%
3’UTR_variant	88639	3’UTR_variant	0.20%
5’UTR_variant	16109	5’UTR_variant	0.05%
5’UTR_premature_start_codon_gain_variant	2698	5’UTR_variant	
splice_region_variant&synonymous_variant	3439	Other_regulatory	0.12%
stop_gained	3224	Other_regulatory	
splice_region_variant&intron_variant	23960	Other_regulatory	
splice_region_variant	1039	Other_regulatory	
splice_donor_variant&intron_variant	1614	Other_regulatory	
splice_acceptor_variant&intron_variant	1430	Other_regulatory	
missense_variant&splice_region_variant	3949	Other_regulatory	
inframe_deletion	958	Other_regulatory	
frameshift_variant	1285	Other_regulatory	
inframe_insertion	134	Other_regulatory	
splice_region_variant&non_coding_exon_variant	426	Other_regulatory	
disruptive_inframe_deletion	529	Other_regulatory	
frameshift_variant&stop_gained	19	Other_regulatory	

frameshift_variant&splice_region_variant	133	Other_regulatory
stop_gained&splice_region_variant	152	Other_regulatory
start_lost	173	Other_regulatory
splice_acceptor_variant&splice_donor_variant&intron_variant	182	Other_regulatory
disruptive_inframe_insertion	76	Other_regulatory
splice_donor_variant&splice_region_variant&intron_variant	88	Other_regulatory
splice_acceptor_variant&splice_region_variant&intron_variant	115	Other_regulatory
stop_retained_variant	71	Other_regulatory
disruptive_inframe_deletion&splice_region_variant	11	Other_regulatory
frameshift_variant&splice_acceptor_variant&splice_region_variant&intron_variant	23	Other_regulatory
stop_lost&splice_region_variant	47	Other_regulatory
frameshift_variant&splice_donor_variant&splice_region_variant&intron_variant	28	Other_regulatory
stop_lost	76	Other_regulatory
splice_region_variant&stop_retained_variant	34	Other_regulatory
initiator_codon_variant	21	Other_regulatory
disruptive_inframe_insertion&splice_region_variant	1	Other_regulatory
frameshift_variant&stop_lost	8	Other_regulatory
frameshift_variant&stop_lost&splice_region_variant	4	Other_regulatory
frameshift_variant&start_lost	7	Other_regulatory
stop_gained&inframe_insertion	5	Other_regulatory

frameshift_variant&start_lost&splice_region_variant	1	Other_regulatory	
Total SNP and Indel	38318974		100%

* The proportion was calculated as numbers of SNPs in a class divided by total SNPs/Indel and then multiplied by 100

Table S4. Functional annotations of SNPs in the 50K SNP panel after quality control along with the number of variants in each class, classification of SNP functions, percentage of WGS and 9 functional class assignments

Functional class annotation	Number of SNPs	Classification of SNP functions	Proportion*
intergenic_region	18313	intergenic_region	60.73%
downstream_gene_variant	1138	downstream_gene_variant	3.77%
upstream_gene_variant	1308	upstream_gene_variant	4.34%
non_coding_transcript_exon_variant	15	synonymous_variant	1.75%
synonymous_variant	513	synonymous_variant	
intron_variant	8528	intron_variant	28.28%
missense_variant	68	missense_variant	0.23%
3’UTR_variant	193	3’UTR_variant	0.64%
5’UTR_premature_start_codon_gain_variant	4	5’UTR_variant	0.10%
5’UTR_variant	26	5’UTR_variant	
initiator_codon_variant&non_canonical_start_codon	1	Other_regulatory	0.16%
missense_variant&splice_region_variant	3	Other_regulatory	
splice_region_variant	1	Other_regulatory	
splice_region_variant&intron_variant	27	Other_regulatory	

splice_region_variant&non_coding_transcript_exon_variant	2	Other_regulatory	
splice_region_variant&synonymous_variant	13	Other_regulatory	
stop_retained_variant	2	Other_regulatory	
total	30155		100.00%

* The proportion was calculated as numbers of SNPs in a class divided by total SNPs/Indel and then multiplied by 100

Table S5. List of lead SNPs that were overlapped with QTLs published in Cattle QTL database within 1M bp up or 1M bp downstream for RFI, DMI, ADG, and MWT, where the last column shows a list of QTL ids with reference numbers in square brackets. Overlapped QTLs within 70k bp up or 70k bp downstream were highlighted in bold. References were listed at the end of the file.

Trait	SNP	Chr	bp	No. of overlapped QTLs	Overlapped QTL ids [reference]
RFI	rs109479784	1	121176492	1	121956[7]
RFI	rs110523019	3	6835555	1	21045[27]
RFI	rs382972340	12	54262083	1	21067[27]
RFI	Chr15:82875910	15	82875910	3	131319[2], 131337[2], **56460[14]**
DMI	rs384466084	6	31945021	1	23691[22]
DMI	rs463483203	6	32301275	1	23691[22]
DMI	rs384405067	6	39719047	1	23734[22]
DMI	rs383608859	6	39967379	1	23734[22]
DMI	rs110280465	6	40041892	1	23734[22]
DMI	rs136513257	6	40422038	1	23734[22]

DMI	rs110650030	6	41213430	1	23734[22]
DMI	rs210962809	6	41333075	1	23734[22]
DMI	rs110785425	10	70651353	1	23800[22]
ADG	rs382130743	2	36556122	1	23641[22]
ADG	Chr4:112725016	4	112725016	1	20670[28]
ADG	rs137822220	5	106247266	13	**131017[2], 131018[2], 131034[2], 131035[2], 131036[2], 131038[2], 131039[2], 131040[2], 131042[2], 131043[2], 131044[2], 131045[2], 56473[14]**
ADG	rs380377923	6	31546181	2	102120[8], **22808[23]**
ADG	rs136764476	6	31656705	2	102120[8], 22808[23]
ADG	rs110967603	6	35951727	5	102117[8], 102118[8], 102122[8], 102124[8], **25113[18]**
ADG	rs378678939	6	36714098	1	25113[18]
ADG	rs385661392	6	37389583	1	102014[9]
ADG	rs133323815	6	37790429	33	124027[5], 124028[5], 124029[5], 124030[5], 124031[5], 124032[5], 124033[5], 124034[5], 124035[5], 124044[5], 124045[5], 124046[5], 124047[5], 124048[5], 124049[5], 124053[5], 124058[5], 124059[5], 124060[5], 124061[5], 124062[5], 124067[5], 124068[5], 124070[5], 124071[5], 124073[5], 124074[5], 124079[5], 102014[9], 20086[30], 20088[30], 20089[30], 20091[30]
ADG	Chr6:37862012	6	37862012	47	124027[5], 124028[5], 124029[5], 124030[5], 124031[5], 124032[5], 124033[5], 124034[5], 124035[5], 124036[5], 124037[5], 124038[5], 124039[5], 124043[5], 124044[5], 124045[5], 124046[5], 124047[5], 124048[5], 124049[5], 124050[5], 124051[5], 124052[5], 124053[5], 124057[5], 124058[5], 124059[5], 124060[5], 124061[5], 124062[5], 124063[5], 124064[5], 124065[5], 124067[5], 124068[5], 124070[5], 124071[5], 124073[5], 124074[5], 124079[5], 102014[9], 20086[30], 20088[30], 20089[30], 20091[30], 20094[30], 20095[30]

ADG	rs381043912	6	37957342	55	124027[5], 124028[5], 124029[5], 124030[5], 124031[5], 124032[5], 124033[5], 124034[5], 124035[5], 124036[5], 124037[5], 124038[5], 124039[5], 124040[5], 124041[5], 124042[5], 124043[5], 124044[5], 124045[5], 124046[5], 124047[5], 124048[5], 124049[5], 124050[5], 124051[5], 124052[5], 124053[5], 124054[5], 124055[5], 124057[5], 124058[5], 124059[5], 124060[5], 124061[5], 124062[5], 124063[5], 124064[5], 124065[5], 124067[5], 124068[5], 124069[5], 124070[5], 124071[5], 124073[5], 124074[5], 124079[5], 102014[9], 20086[30], 20088[30], 20089[30], 20091[30], 20094[30], 20095[30], 20097[30], 20099[30]
ADG	rs383638885	6	38031945	56	124027[5], 124028[5], 124029[5], 124030[5], 124031[5], 124032[5], 124033[5], 124034[5], 124035[5], 124036[5], 124037[5], 124038[5], 124039[5], 124040[5], 124041[5], 124042[5], 124043[5], 124044[5], 124045[5], 124046[5], 124047[5], 124048[5], 124049[5], 124050[5], 124051[5], 124052[5], 124053[5], 124054[5], 124055[5], 124057[5], 124058[5], 124059[5], 124060[5], 124061[5], 124062[5], 124063[5], 124064[5], 124065[5], 124067[5], 124068[5], 124069[5], 124070[5], 124071[5], 124073[5], 124074[5], 124079[5], 102014[9], 20086[30], 20088[30], 20089[30], 20091[30], 20094[30], 20095[30], 20097[30], 20099[30], 20101[30]
ADG	rs210353492	6	38107063	56	124027[5], 124028[5], 124029[5], 124030[5], 124031[5], 124032[5], 124033[5], 124034[5], 124035[5], 124036[5], 124037[5], 124038[5], 124039[5], 124040[5], 124041[5], 124042[5], 124043[5], 124044[5], 124045[5], 124046[5], 124047[5], 124048[5], 124049[5], 124050[5], 124051[5], 124052[5], 124053[5], 124054[5], 124055[5], 124057[5], 124058[5], 124059[5], 124060[5], 124061[5], 124062[5], 124063[5], 124064[5], 124065[5], 124067[5], 124068[5], 124069[5], 124070[5], 124071[5], 124073[5], 124074[5], 124079[5], 102014[9], 20086[30], 20088[30], 20089[30], 20091[30], 20094[30], 20095[30], 20097[30], 20099[30], 20101[30]
ADG	rs207550149	6	38180681	56	124027[5], 124028[5], 124029[5], 124030[5], 124031[5], 124032[5], 124033[5], 124034[5], 124035[5], 124036[5], 124037[5], 124038[5], 124039[5], 124040[5], 124041[5], 124042[5], 124043[5], 124044[5], 124045[5], 124046[5], 124047[5], 124048[5], 124049[5], 124050[5], 124051[5], 124052[5], 124053[5], 124054[5], 124055[5], 124057[5], 124058[5], 124059[5], 124060[5], 124061[5], 124062[5], 124063[5], 124064[5], 124065[5], 124067[5], 124068[5], 124069[5], 124070[5], 124071[5], 124073[5], 124074[5], 124079[5], 102014[9], 20086[30], 20088[30], 20089[30], 20091[30], 20094[30], 20095[30], 20097[30], 20099[30], 20101[30]

ADG	rs209915052	6	38259595	56	124027[5], 124028[5], 124029[5], 124030[5], 124031[5], 124032[5], 124033[5], 124034[5], 124035[5], 124036[5], 124037[5], 124038[5], 124039[5], 124040[5], 124041[5], 124042[5], 124043[5], 124044[5], 124045[5], 124046[5], 124047[5], 124048[5], 124049[5], 124050[5], 124051[5], 124052[5], 124053[5], 124054[5], 124055[5], 124057[5], 124058[5], 124059[5], 124060[5], 124061[5], 124062[5], 124063[5], 124064[5], 124065[5], 124067[5], 124068[5], 124069[5], 124070[5], 124071[5], 124073[5], 124074[5], 124079[5], 102014[9], 20086[30], 20088[30], 20089[30], 20091[30], 20094[30], 20095[30], 20097[30], 20099[30], 20101[30]
ADG	rs382462900	6	38429007	56	124027[5], 124028[5], 124029[5], 124030[5], 124031[5], 124032[5], 124033[5], 124034[5], 124035[5], 124036[5], 124037[5], 124038[5], 124039[5], 124040[5], 124041[5], 124042[5], 124043[5], 124044[5], 124045[5], 124046[5], 124047[5], 124048[5], 124049[5], 124050[5], 124051[5], 124052[5], 124053[5], 124054[5], 124055[5], 124057[5], 124058[5], 124059[5], 124060[5], 124061[5], 124062[5], 124063[5], 124064[5], 124065[5], 124067[5], 124068[5], 124069[5], 124070[5], 124071[5], 124073[5], **124074[5]**, 124079[5], 102014[9], 20086[30], 20088[30], 20089[30], 20091[30], 20094[30], 20095[30], 20097[30], 20099[30], 20101[30]
ADG	rs385289515	6	38509747	56	124027[5], 124028[5], 124029[5], 124030[5], 124031[5], 124032[5], 124033[5], 124034[5], 124035[5], 124036[5], 124037[5], 124038[5], 124039[5], 124040[5], 124041[5], 124042[5], 124043[5], 124044[5], 124045[5], 124046[5], 124047[5], **124048[5]**, 124049[5], 124050[5], 124051[5], 124052[5], 124053[5], 124054[5], 124055[5], 124057[5], 124058[5], 124059[5], 124060[5], 124061[5], 124062[5], 124063[5], 124064[5], 124065[5], 124067[5], 124068[5], 124069[5], 124070[5], 124071[5], 124073[5], **124074[5]**, 124079[5], 102014[9], 20086[30], 20088[30], 20089[30], 20091[30], 20094[30], 20095[30], 20097[30], 20099[30], 20101[30]
ADG	Chr6:38606864	6	38606864	56	124027[5], 124028[5], 124029[5], 124030[5], 124031[5], 124032[5], 124033[5], 124034[5], 124035[5], 124036[5], 124037[5], 124038[5], 124039[5], 124040[5], 124041[5], 124042[5], 124043[5], 124044[5], 124045[5], 124046[5], 124047[5], **124048[5]**, 124049[5], 124050[5], 124051[5], 124052[5], 124053[5], 124054[5], 124055[5], 124057[5], 124058[5], 124059[5], 124060[5], 124061[5], 124062[5], 124063[5], 124064[5], 124065[5], 124067[5], 124068[5], 124069[5], **124070[5]**, 124071[5], 124073[5], 124074[5], **124079[5]**, 102014[9], 20086[30], 20088[30], 20089[30], 20091[30], 20094[30], 20095[30], 20097[30], 20099[30], 20101[30]

ADG	rs381763403	6	38681815	56	**124027[5], 124028[5]**, 124029[5], **124030[5]**, 124031[5], **124032[5]**, **124033[5]**, 124034[5], **124035[5]**, 124036[5], 124037[5], 124038[5], 124039[5], 124040[5], 124041[5], 124042[5], 124043[5], **124044[5], 124045[5], 124046[5], 124047[5],** 124048[5], **124049[5]**, 124050[5], 124051[5], 124052[5], 124053[5], 124054[5], 124055[5], 124057[5], 124058[5], 124059[5], 124060[5], 124061[5], 124062[5], 124063[5], 124064[5], 124065[5], **124067[5]**, **124068[5]**, 124069[5], **124070[5]**, **124071[5], 124073[5]**, 124074[5], **124079[5]**, 102014[9], 20086[30], 20088[30], 20089[30], 20091[30], 20094[30], 20095[30], 20097[30], 20099[30], 20101[30]
ADG	rs110302982	6	38760889	56	**124027[5], 124028[5], 124029[5], 124030[5], 124031[5], 124032[5], 124033[5], 124034[5], 124035[5], 124036[5], 124037[5]**, 124038[5], 124039[5], 124040[5], 124041[5], 124042[5], 124043[5], **124044[5], 124045[5], 124046[5], 124047[5]**, 124048[5], **124049[5], 124050[5], 124051[5], 124052[5], 124053[5]**, 124054[5], 124055[5], 124057[5], **124058[5], 124059[5], 124060[5], 124061[5], 124062[5], 124063[5]**, 124064[5], 124065[5], **124067[5], 124068[5]**, 124069[5], 124070[5], **124071[5], 124073[5]**, 124074[5], 124079[5], 102014[9], **20086[30], 20088[30], 20089[30], 20091[30], 20094[30], 20095[30]**, 20097[30], 20099[30], 20101[30]
ADG	rs109696064	6	38837730	56	124027[5], 124028[5], 124029[5], 124030[5], 124031[5], 124032[5], 124033[5], 124034[5], 124035[5], **124036[5], 124037[5], 124038[5], 124039[5], 124040[5], 124041[5], 124042[5], 124043[5]**, 124044[5], 124045[5], 124046[5], 124047[5], 124048[5], 124049[5], **124050[5], 124051[5], 124052[5]**, 124053[5], **124054[5], 124055[5], 124057[5], 124058[5], 124059[5], 124060[5], 124061[5], 124062[5], 124063[5], 124064[5], 124065[5],** 124067[5], **124068[5], 124069[5]**, 124070[5], 124071[5], 124073[5], 124074[5], 124079[5], 102014[9], **20086[30], 20088[30], 20089[30], 20091[30], 20094[30], 20095[30], 20097[30], 20099[30]**, 20101[30]
ADG	rs110995268	6	38914196	56	124027[5], 124028[5], 124029[5], 124030[5], 124031[5], 124032[5], 124033[5], 124034[5], 124035[5], 124036[5], 124037[5], 124038[5], 124039[5], **124040[5], 124041[5], 124042[5], 124043[5]**, 124044[5], 124045[5], 124046[5], 124047[5], 124048[5], 124049[5], 124050[5], 124051[5], 124052[5], 124053[5], **124054[5], 124055[5]**, 124057[5], 124058[5], 124059[5], 124060[5], 124061[5], 124062[5], 124063[5], 124064[5], **124065[5]**, 124067[5], 124068[5], **124069[5]**, 124070[5], 124071[5], 124073[5], 124074[5], 124079[5], 102014[9], 20086[30], 20088[30], 20089[30], 20091[30], 20094[30], 20095[30], **20097[30], 20099[30]**, 20101[30]

ADG	rs110001259	6	39026381	56	124027[5], 124028[5], 124029[5], 124030[5], 124031[5], 124032[5], 124033[5], 124034[5], 124035[5], 124036[5], 124037[5], 124038[5], 124039[5], 124040[5], 124041[5], 124042[5], 124043[5], 124044[5], 124045[5], 124046[5], 124047[5], 124048[5], 124049[5], 124050[5], 124051[5], 124052[5], 124053[5], 124054[5], 124055[5], 124057[5], 124058[5], 124059[5], 124060[5], 124061[5], 124062[5], 124063[5], 124064[5], 124065[5], 124067[5], 124068[5], 124069[5], 124070[5], 124071[5], 124073[5], 124074[5], 124079[5], 102014[9], 20086[30], 20088[30], 20089[30], 20091[30], 20094[30], 20095[30], 20097[30], 20099[30], **20101[30]**
ADG	rs110987922	6	39113335	56	124027[5], 124028[5], 124029[5], 124030[5], 124031[5], 124032[5], 124033[5], 124034[5], 124035[5], 124036[5], 124037[5], 124038[5], 124039[5], 124040[5], 124041[5], 124042[5], 124043[5], 124044[5], 124045[5], 124046[5], 124047[5], 124048[5], 124049[5], 124050[5], 124051[5], 124052[5], 124053[5], 124054[5], 124055[5], 124057[5], 124058[5], 124059[5], 124060[5], 124061[5], 124062[5], 124063[5], 124064[5], 124065[5], 124067[5], 124068[5], 124069[5], 124070[5], 124071[5], 124073[5], 124074[5], 124079[5], 102014[9], 20086[30], 20088[30], 20089[30], 20091[30], 20094[30], 20095[30], 20097[30], 20099[30], 20101[30]
ADG	rs110218841	6	39214342	56	124027[5], 124028[5], 124029[5], 124030[5], 124031[5], 124032[5], 124033[5], 124034[5], 124035[5], 124036[5], 124037[5], 124038[5], 124039[5], 124040[5], 124041[5], 124042[5], 124043[5], 124044[5], 124045[5], 124046[5], 124047[5], 124048[5], 124049[5], 124050[5], 124051[5], 124052[5], 124053[5], 124054[5], 124055[5], 124057[5], 124058[5], 124059[5], 124060[5], 124061[5], 124062[5], 124063[5], 124064[5], 124065[5], 124067[5], 124068[5], 124069[5], 124070[5], 124071[5], 124073[5], 124074[5], 124079[5], 102014[9], 20086[30], 20088[30], 20089[30], 20091[30], 20094[30], 20095[30], 20097[30], 20099[30], 20101[30]
ADG	rs110747048	6	39353897	55	124027[5], 124028[5], 124029[5], 124030[5], 124031[5], 124032[5], 124033[5], 124034[5], 124035[5], 124036[5], 124037[5], 124038[5], 124039[5], 124040[5], 124041[5], 124042[5], 124043[5], 124044[5], 124045[5], 124046[5], 124047[5], 124048[5], 124049[5], 124050[5], 124051[5], 124052[5], 124053[5], 124054[5], 124055[5], 124057[5], 124058[5], 124059[5], 124060[5], 124061[5], 124062[5], 124063[5], 124064[5], 124065[5], 124067[5], 124068[5], 124069[5], 124070[5], 124071[5], 124073[5], 124074[5], 124079[5], 20086[30], 20088[30], 20089[30], 20091[30], 20094[30], 20095[30], 20097[30], 20099[30], 20101[30]

ADG	rs109433941	6	39444050	55	124027[5], 124028[5], 124029[5], 124030[5], 124031[5], 124032[5], 124033[5], 124034[5], 124035[5], 124036[5], 124037[5], 124038[5], 124039[5], 124040[5], 124041[5], 124042[5], 124043[5], 124044[5], 124045[5], 124046[5], 124047[5], 124048[5], 124049[5], 124050[5], 124051[5], 124052[5], 124053[5], 124054[5], 124055[5], 124057[5], 124058[5], 124059[5], 124060[5], 124061[5], 124062[5], 124063[5], 124064[5], 124065[5], 124067[5], 124068[5], 124069[5], 124070[5], 124071[5], 124073[5], 124074[5], 124079[5], 20086[30], 20088[30], 20089[30], 20091[30], 20094[30], 20095[30], 20097[30], 20099[30], 20101[30]
ADG	rs133877406	6	39571260	54	124027[5], 124028[5], 124029[5], 124030[5], 124031[5], 124032[5], 124033[5], 124034[5], 124035[5], 124036[5], 124037[5], 124038[5], 124039[5], 124040[5], 124041[5], 124042[5], 124043[5], 124044[5], 124045[5], 124046[5], 124047[5], 124048[5], 124049[5], 124050[5], 124051[5], 124052[5], 124053[5], 124054[5], 124055[5], 124057[5], 124058[5], 124059[5], 124060[5], 124061[5], 124062[5], 124063[5], 124064[5], 124065[5], 124067[5], 124068[5], 124069[5], 124070[5], 124071[5], 124073[5], 124079[5], 20086[30], 20088[30], 20089[30], 20091[30], 20094[30], 20095[30], 20097[30], 20099[30], 20101[30]
ADG	rs385370479	6	39642714	53	124027[5], 124028[5], 124029[5], 124030[5], 124031[5], 124032[5], 124033[5], 124034[5], 124035[5], 124036[5], 124037[5], 124038[5], 124039[5], 124040[5], 124041[5], 124042[5], 124043[5], 124044[5], 124045[5], 124046[5], 124047[5], 124049[5], 124050[5], 124051[5], 124052[5], 124053[5], 124054[5], 124055[5], 124057[5], 124058[5], 124059[5], 124060[5], 124061[5], 124062[5], 124063[5], 124064[5], 124065[5], 124067[5], 124068[5], 124069[5], 124070[5], 124071[5], 124073[5], 124079[5], 20086[30], 20088[30], 20089[30], 20091[30], 20094[30], 20095[30], 20097[30], 20099[30], 20101[30]
ADG	rs384405067	6	39719047	48	124027[5], 124028[5], 124029[5], 124030[5], 124031[5], 124032[5], 124033[5], 124034[5], 124036[5], 124037[5], 124038[5], 124039[5], 124040[5], 124041[5], 124042[5], 124043[5], 124044[5], 124045[5], 124046[5], 124049[5], 124050[5], 124051[5], 124052[5], 124053[5], 124054[5], 124055[5], 124057[5], 124058[5], 124059[5], 124060[5], 124061[5], 124062[5], 124063[5], 124064[5], 124065[5], 124067[5], 124068[5], 124069[5], 124073[5], 20086[30], 20088[30], 20089[30], 20091[30], 20094[30], 20095[30], 20097[30], 20099[30], 20101[30]
ADG	rs382149180	6	39947443	3	102116[8], 102121[8], 20101[30]
ADG	rs110280465	6	40041892	2	102116[8], 102121[8]
ADG	rs43458971	6	40302828	2	102116[8], 102121[8]

ADG	rs109823986	6	40702520	2	102116[8], 102121[8]
ADG	rs385648596	6	40900316	2	102116[8], **102121[8]**
ADG	rs134606084	6	40970705	2	102116[8], 102121[8]
ADG	rs43464551	6	41047507	2	102116[8], 102121[8]
ADG	rs135202983	6	41229657	3	102116[8], 102121[8], 124076[5]
ADG	rs43466307	6	42370579	2	124076[5], 22810[23]
ADG	rs209393630	6	42510243	2	124076[5], 22810[23]
ADG	rs381205796	6	42636544	2	124076[5], 22810[23]
ADG	Chr6:43316828	6	43316828	1	**22810[23]**
ADG	Chr6:44029020	6	44029020	1	22810[23]
ADG	rs137370999	6	44133101	1	22810[23]
ADG	rs135988356	6	46224508	1	23755[22]
ADG	Chr6:46325186	6	46325186	1	23755[22]
ADG	rs110990855	6	46460427	1	**23755[22]**
ADG	rs381809466	6	46569119	1	**23755[22]**
ADG	rs134334613	6	46729101	1	23755[22]
ADG	rs379061965	6	46871416	1	23755[22]
ADG	rs134663272	6	47128886	1	23755[22]
ADG	rs110114429	7	93148272	55	131019[2], 131020[2], 131021[2], 131022[2], 131023[2], 131024[2], **131025[2], 131026[2], 131027[2], 131028[2]**, 131029[2], **131037[2], 131041[2],** 131046[2], 131047[2], 131048[2], 131049[2], **131050[2], 131051[2],** 131052[2], 131055[2], **131056[2], 131057[2]**, 131058[2], 131059[2], 131060[2], 131063[2], 131064[2], 131072[2], 131073[2], 131074[2], 131075[2], 131076[2], 131077[2], 131078[2], 131079[2], 131080[2], 131081[2], 131082[2], 131083[2], 131095[2], 131096[2], 131097[2], 131099[2], 131100[2], 131101[2], 131110[2], 131112[2], 131119[2], 131120[2], 131123[2], 101982[9], **22785[23]**, 131098[2], **56474[14]**

ADG	rs109901274	7	93244933	55	**131019[2], 131020[2], 131021[2], 131022[2], 131023[2], 131024[2], 131025[2], 131026[2], 131027[2], 131028[2], 131029[2]**, 131037[2], 131041[2], 131046[2], 131047[2], 131048[2], 131049[2], **131050[2], 131051[2],** 131052[2], 131055[2], **131056[2], 131057[2], 131058[2], 131059[2], 131060[2]**, 131063[2], 131064[2], 131072[2], 131073[2], 131074[2], 131075[2], 131076[2], 131077[2], 131078[2], 131079[2], 131080[2], 131081[2], 131082[2], 131083[2], 131095[2], 131096[2], 131097[2], 131099[2], 131100[2], 131101[2], 131110[2], 131112[2], 131119[2], 131120[2], 131123[2], **101982[9]**, **22785[23]**, 131098[2], **56474[14]**
ADG	rs41681899	13	18998047	1	23831[22]
ADG	rs41693642	13	45111501	1	23220[19]
ADG	Chr13:45255892	13	45255892	1	23220[19]
ADG	rs42853892	13	45349576	1	23220[19]
ADG	rs208583895	14	24153141	2	102021[9], 20873[25]
ADG	rs110220085	14	24235302	3	102021[9], 20873[25], 20848[26]
ADG	rs211486269	14	24508890	8	102021[9], 20873[25], 20848[26], 20849[26], 20850[26], 20851[26], 20852[26], 20853[26]
ADG	rs41724405	14	24626845	8	102021[9], 20873[25], 20848[26], 20849[26], 20850[26], 20851[26], 20852[26], 20853[26]
ADG	rs110144371	14	24895889	8	102021[9], 20873[25], 20848[26], 20849[26], 20850[26], 20851[26], 20852[26], 20853[26]
ADG	rs134215421	14	25006125	8	**102021[9]**, **20873[25],** 20848[26], 20849[26], 20850[26], 20851[26], 20852[26], 20853[26]
ADG	rs109060535	14	26223578	6	20848[26], 20849[26], 20850[26], 20851[26], 20852[26], 20853[26]
ADG	rs210931678	20	4277046	3	130810[2], 22839[23], 22840[23]
ADG	rs41933279	20	4583717	2	22839[23], 22840[23]
ADG	rs43349801	20	4662257	2	22839[23], 22840[23]
ADG	rs43349740	20	4778613	2	**22839[23],** 22840[23]
ADG	rs42661323	20	4916731	2	**22839[23], 22840[23]**
ADG	rs473496654	20	5006104	2	**22839[23], 22840[23]**
ADG	rs448890458	25	40587255	2	101999[9], 102041[9]
MWT	rs110358394	5	106266665	1	140481[1]

MWT	rs210609295	6	31853453	1	131411[2]
MWT	rs384466084	6	31945021	1	**131411[2]**
MWT	rs386082192	6	32467576	1	131411[2]
MWT	rs109672376	6	35634137	1	25112[18]
MWT	Chr6:35727960	6	35727960	1	25112[18]
MWT	rs463408175	6	35815378	1	25112[18]
MWT	rs109168002	6	35892044	1	**25112[18]**
MWT	rs135391932	6	35985958	1	**25112[18]**
MWT	Chr6:36063637	6	36063637	1	25112[18]
MWT	Chr6:36142577	6	36142577	1	25112[18]
MWT	rs207985227	6	36217568	1	25112[18]
MWT	Chr6:36358198	6	36358198	1	25112[18]
MWT	rs110173598	6	36430643	1	25112[18]
MWT	rs207995078	6	36597371	1	25112[18]
MWT	rs137290140	6	37007648	1	130915[2]
MWT	rs208802803	6	37312750	2	130915[2], 25114[18]
MWT	rs385661392	6	37389583	2	130915[2], 25114[18]
MWT	rs211048534	6	37461131	2	130915[2], 25114[18]
MWT	rs109100620	6	37595062	3	131414[2], 130915[2], 25114[18]
MWT	Chr6:37666690	6	37666690	3	131414[2], 130915[2], 25114[18]
MWT	rs110111900	6	37791785	4	131414[2], **130915[2]**, 25114[18], 23717[22]
MWT	rs383473904	6	37872592	4	131414[2], **130915[2]**, 25114[18], 23717[22]
MWT	Chr6:37959886	6	37959886	5	131414[2], 130915[2], 25114[18], 23717[22], 23723[22]
MWT	rs383638885	6	38031945	5	131414[2], 130915[2], **25114[18]**, 23717[22], 23723[22]
MWT	rs210353492	6	38107063	5	131414[2], 130915[2], **25114[18]**, 23717[22], 23723[22]

MWT	rs207550149	6	38180681	5	131414[2], 130915[2], 25114[18], 23717[22], 23723[22]
MWT	rs379490408	6	38253085	9	131388[2], 131389[2], 131393[2], 131394[2], 131414[2], 130915[2], 25114[18], 23717[22], 23723[22]
MWT	rs382462900	6	38429007	18	131382[2], 131383[2], 131384[2], 131385[2], 131388[2], 131389[2], 131390[2], 131391[2], 131392[2], 131393[2], 131394[2], 131395[2], 131398[2], 131414[2], 130915[2], 25114[18], 23717[22], 23723[22]
MWT	rs385289515	6	38509747	18	131382[2], 131383[2], 131384[2], 131385[2], 131388[2], 131389[2], 131390[2], 131391[2], 131392[2], 131393[2], 131394[2], 131395[2], 131398[2], **131414[2]**, 130915[2], 25114[18], 23717[22], 23723[22]
MWT	rs134828344	6	38587035	18	131382[2], 131383[2], 131384[2], 131385[2], 131388[2], 131389[2], 131390[2], 131391[2], 131392[2], 131393[2], 131394[2], 131395[2], 131398[2], **131414[2],** 130915[2], 25114[18], 23717[22], 23723[22]
MWT	rs109355965	6	38657124	18	131382[2], 131383[2], 131384[2], 131385[2], 131388[2], 131389[2], 131390[2], 131391[2], 131392[2], 131393[2], 131394[2], 131395[2], 131398[2], 131414[2], 130915[2], 25114[18], **23717[22]**, 23723[22]
MWT	rs109843602	6	38750035	18	131382[2], 131383[2], 131384[2], 131385[2], 131388[2], 131389[2], 131390[2], 131391[2], 131392[2], 131393[2], 131394[2], 131395[2], 131398[2], 131414[2], 130915[2], 25114[18], **23717[22]**, 23723[22]
MWT	rs109732906	6	38872172	17	131382[2], 131383[2], 131384[2], 131385[2], 131388[2], 131389[2], 131390[2], 131391[2], 131392[2], 131393[2], 131394[2], 131395[2], 131398[2], 131414[2], 25114[18], 23717[22], **23723[22]**
MWT	rs110369727	6	38947564	17	131382[2], 131383[2], 131384[2], 131385[2], 131388[2], 131389[2], 131390[2], 131391[2], 131392[2], 131393[2], 131394[2], 131395[2], 131398[2], 131414[2], 25114[18], 23717[22], **23723[22]**
MWT	rs378858709	6	39020582	17	131382[2], 131383[2], 131384[2], 131385[2], 131388[2], 131389[2], 131390[2], 131391[2], 131392[2], 131393[2], 131394[2], 131395[2], 131398[2], 131414[2], 25114[18], 23717[22], 23723[22]
MWT	Chr6:39111019	6	39111019	16	131382[2], 131383[2], 131384[2], 131385[2], 131388[2], 131389[2], 131390[2], 131391[2], 131392[2], 131393[2], 131394[2], 131395[2], 131398[2], 131414[2], 23717[22], 23723[22]
MWT	rs109658371	6	39213566	16	**131382[2], 131383[2], 131384[2], 131385[2], 131388[2], 131389[2], 131390[2], 131391[2], 131392[2], 131393[2], 131394[2], 131395[2], 131398[2]**, 131414[2], 23717[22], 23723[22]

MWT	rs109840670	6	39364500	16	131382[2], 131383[2], 131384[2], 131385[2], 131388[2], 131389[2], 131390[2], 131391[2], 131392[2], 131393[2], 131394[2], 131395[2], 131398[2], 131414[2], 23717[22], 23723[22]
MWT	rs109433941	6	39444050	16	131382[2], 131383[2], 131384[2], 131385[2], 131388[2], 131389[2], 131390[2], 131391[2], 131392[2], 131393[2], 131394[2], 131395[2], 131398[2], 131414[2], 23717[22], 23723[22]
MWT	rs133877406	6	39571260	16	131382[2], 131383[2], 131384[2], 131385[2], 131388[2], 131389[2], 131390[2], 131391[2], 131392[2], 131393[2], 131394[2], 131395[2], 131398[2], 131414[2], 23717[22], 23723[22]
MWT	rs385370479	6	39642714	16	131382[2], 131383[2], 131384[2], 131385[2], 131388[2], 131389[2], 131390[2], 131391[2], 131392[2], 131393[2], 131394[2], 131395[2], 131398[2], 23717[22], 23723[22], 23733[22]
MWT	rs384405067	6	39719047	15	131382[2], 131383[2], 131384[2], 131385[2], 131388[2], 131389[2], 131390[2], 131391[2], 131392[2], 131393[2], 131394[2], 131395[2], 131398[2], 23723[22], 23733[22]
MWT	rs381670901	6	39868660	15	131382[2], 131383[2], 131384[2], 131385[2], 131388[2], 131389[2], 131390[2], 131391[2], 131392[2], 131393[2], 131394[2], 131395[2], 131398[2], 23723[22], 23733[22]
MWT	rs382149180	6	39947443	14	131382[2], 131383[2], 131384[2], 131385[2], 131388[2], 131389[2], 131390[2], 131391[2], 131392[2], 131393[2], 131394[2], 131395[2], 131398[2], 23733[22]
MWT	rs378255303	6	40029961	14	131382[2], 131383[2], 131384[2], 131385[2], 131388[2], 131389[2], 131390[2], 131391[2], 131392[2], 131393[2], 131394[2], 131395[2], 131398[2], 23733[22]
MWT	rs43456300	6	40141451	14	131382[2], 131383[2], 131384[2], 131385[2], 131388[2], 131389[2], 131390[2], 131391[2], 131392[2], 131393[2], 131394[2], 131395[2], 131398[2], 23733[22]
MWT	rs111031148	6	40245181	14	131382[2], 131383[2], 131384[2], 131385[2], 131388[2], 131389[2], 131390[2], 131391[2], 131392[2], 131394[2], 131395[2], 131398[2], 23733[22], 23738[22]
MWT	rs43070338	6	40421678	2	23733[22], 23738[22]
MWT	rs385648596	6	40900316	2	23733[22], 23738[22]
MWT	rs43461215	6	41190295	2	23733[22], **23738[22]**
MWT	rs385048356	6	41705949	1	23738[22]
MWT	Chr6:41779679	6	41779679	1	23738[22]
MWT	rs381611273	6	49301672	1	131345[2]

MWT	rs385825767	6	49388298	1	131345[2]
MWT	rs211227700	20	4110200	14	131135[2], 131137[2], 131138[2], 131146[2], 131173[2], 131174[2], 131190[2], 131191[2], 131410[2], 131412[2], 131415[2], 131416[2], 131417[2], 131418[2]
MWT	Chr20:4205219	20	4205219	14	131135[2], 131137[2], 131138[2], 131146[2], 131173[2], 131174[2], 131190[2], 131191[2], 131410[2], 131412[2], 131415[2], 131416[2], 131417[2], 131418[2]
MWT	rs210931678	20	4277046	14	131135[2], 131137[2], 131138[2], 131146[2], 131173[2], 131174[2], 131190[2], 131191[2], 131410[2], 131412[2], 131415[2], 131416[2], 131417[2], 131418[2]
MWT	rs41934045	20	4563925	14	131135[2], **131137[2], 131138[2]**, 131146[2], **131173[2], 131174[2]**, 131190[2], 131191[2], 131410[2], 131412[2], 131415[2], 131416[2], 131417[2], 131418[2]
MWT	rs43349810	20	4637730	14	131135[2], **131137[2], 131138[2]**, 131146[2], **131173[2], 131174[2]**, 131190[2], 131191[2], 131410[2], 131412[2], 131415[2], 131416[2], 131417[2], 131418[2]
MWT	rs43349740	20	4778613	14	131135[2], 131137[2], 131138[2], 131146[2], 131173[2], 131174[2], 131190[2], 131191[2], 131410[2], 131412[2], 131415[2], 131416[2], 131417[2], 131418[2]
MWT	rs41574252	20	4863507	14	**131135[2]**, 131137[2], 131138[2], **131146[2]**, 131173[2], 131174[2], **131190[2]**, **131191[2], 131410[2], 131412[2], 131415[2], 131416[2], 131417[2], 131418[2]**
MWT	rs41574253	20	4964834	14	**131135[2]**, 131137[2], 131138[2], 131146[2], 131173[2], 131174[2], **131190[2], 131191[2]**, 131410[2], 131412[2], 131415[2], 131416[2], **131417[2]**, 131418[2]
MWT	rs211028837	20	5043189	14	131135[2], 131137[2], 131138[2], 131146[2], 131173[2], 131174[2], 131190[2], 131191[2], 131410[2], 131412[2], 131415[2], 131416[2], 131417[2], 131418[2]
MWT	rs483267895	20	5375886	14	131135[2], 131137[2], 131138[2], 131146[2], 131173[2], 131174[2], 131190[2], 131191[2], 131410[2], 131412[2], 131415[2], 131416[2], 131417[2], 131418[2]
MWT	rs207865196	20	5482087	15	130854[2], 131135[2], 131137[2], 131138[2], 131146[2], 131173[2], 131174[2], 131190[2], 131191[2], 131410[2], 131412[2], 131415[2], 131416[2], 131417[2], 131418[2]
MWT	rs42517118	20	5983010	1	130854[2]

List of references for QTLs in the Cattle QTL Database for feed efficiency related traits

Reference Number	Title	Description	Details	ShortDetails	Identifiers
1	The genetic and biological basis of feed efficiency in mid-lactation Holstein dairy cows.	Hardie LC, VandeHaar MJ, Tempelman RJ, Weigel KA, Armentano LE, Wiggans GR, Veerkamp RF, de Haas Y, Coffey MP, Connor EE, Hanigan MD, Staples C, Wang Z, Dekkers JCM, Spurlock DM.	J Dairy Sci. 2017 Nov;100(11):9061-9075. doi: 10.3168/jds.2017-12604. Epub 2017 Aug 23.	J Dairy Sci. 2017	PMID:28843688
2	Genome-wide association study for feed efficiency and growth traits in U.S. beef cattle.	Seabury CM, Oldeschulte DL, Saatchi M, Beever JE, Decker JE, Halley YA, Bhattarai EK, Molaei M, Freetly HC, Hansen SL, Yampara-Iquise H, Johnson KA, Kerley MS, Kim J, Loy DD, Marques E, Neibergs HL, Schnabel RD, Shike DW, Spangler ML, Weaber RL, Garrick DJ, et al.	BMC Genomics. 2017 May 18;18(1):386. doi: 10.1186/s12864-017-3754-y.	BMC Genomics. 2017	PMID:28521758
3	Genome-Wide Association Study for Carcass Traits in an Experimental Nelore Cattle Population.	Medeiros de Oliveira Silva R, Bonvino Stafuzza N, de Oliveira Fragomeni B, Miguel Ferreira de Camargo G, Matos Ceacero T, Noely Dos Santos GonÃ§alves Cyrillo J, Baldi F, Augusti Boligon A, Zerlotti Mercadante ME, Lino Lourenco D, Misztal I, GalvÃ£o de Albuquerque L.	PLoS One. 2017 Jan 24;12(1):e0169860. doi: 10.1371/journal.pone.0169860. eCollection 2017.	PLoS One. 2017	PMID:28118362
4	Genome scan for postmortem carcass traits in Nellore cattle.	JÃºnior GA, Costa RB, de Camargo GM, Carvalheiro R, Rosa GJ, Baldi F, Garcia DA, Gordo DG, Espigolan R, Takada L, MagalhÃ£es AF, Bresolin T, Feitosa FL, Chardulo LA, de Oliveira HN, de Albuquerque LG.	J Anim Sci. 2016 Oct;94(10):4087-4095. doi: 10.2527/jas.2016-0632.	J Anim Sci. 2016	PMID:27898882

5	Multi-strategy genome-wide association studies identify the DCAF16-NCAPG region as a susceptibility locus for average daily gain in cattle.	Zhang W, Li J, Guo Y, Zhang L, Xu L, Gao X, Zhu B, Gao H, Ni H, Chen Y.	Sci Rep. 2016 Nov 28;6:38073. doi: 10.1038/srep38073.	Sci Rep. 2016	PMID:27892541
6	Genetic association of PLAG1, SCD, CYP7B1 and FASN SNPs and their effects on carcass weight, intramuscular fat and fatty acid composition in Hanwoo steers (Korean cattle).	Kim HJ, Sharma A, Lee SH, Lee DH, Lim DJ, Cho YM, Yang BS, Lee SH.	Anim Genet. 2017 Apr;48(2):251-252. doi: 10.1111/age.12523. Epub 2016 Nov 22. No abstract available.	Anim Genet. 2017	PMID:27878829
7	Genomic Regions Associated with Feed Efficiency Indicator Traits in an Experimental Nellore Cattle Population.	Olivieri BF, Mercadante ME, Cyrillo JN, Branco RH, Bonilha SF, de Albuquerque LG, Silva RM, Baldi F.	PLoS One. 2016 Oct 19;11(10):e0164390. doi: 10.1371/journal.pone.0164390. eCollection 2016. Erratum in: PLoS One. 2017 Feb 6;12 (2):e0171845.	PLoS One. 2016	PMID:27760167
8	Genome wide association study on beef production traits in Marchigiana cattle breed.	Sorbolini S, Bongiorni S, Cellesi M, Gaspa G, Dimauro C, Valentini A, Macciotta NP.	J Anim Breed Genet. 2017 Feb;134(1):43-48. doi: 10.1111/jbg.12227. Epub 2016 Jun 22.	J Anim Breed Genet. 2017	PMID:27329851
9	Genome-wide association studies of growth traits in three dairy cattle breeds using whole-genome sequence data.	Mao X, Sahana G, De Koning DJ, Guldbrandtsen B.	J Anim Sci. 2016 Apr;94(4):1426-37. doi: 10.2527/jas.2015-9838.	J Anim Sci. 2016	PMID:27136002

10	Genome-wide association study for carcass traits, fatty acid composition, chemical composition, sugar, and the effects of related candidate genes in Japanese Black cattle.	Sasago N, Abe T, Sakuma H, Kojima T, Uemoto Y.	Anim Sci J. 2017 Jan;88(1):33-44. doi: 10.1111/asj.12595. Epub 2016 Apr 25.	Anim Sci J. 2017	PMID:27112906
11	Multiple Linkage Disequilibrium Mapping Methods to Validate Additive Quantitative Trait Loci in Korean Native Cattle (Hanwoo).	Li Y, Kim JJ.	Asian-Australas J Anim Sci. 2015 Jul;28(7):926-35. doi: 10.5713/ajas.15.0077.	Asian-Australas J Anim Sci. 2015	PMID:26104396
12	A genomewide association mapping study using ultrasound-scanned information identifies potential genomic regions and candidate genes affecting carcass traits in Nellore cattle.	Santana MH, Ventura RV, Utsunomiya YT, Neves HH, Alexandre PA, Oliveira Junior GA, Gomes RC, Bonin MN, Coutinho LL, Garcia JF, Silva SL, Fukumasu H, Leme PR, Ferraz JB.	J Anim Breed Genet. 2015 Dec;132(6):420-7. doi: 10.1111/jbg.12167. Epub 2015 May 27.	J Anim Breed Genet. 2015	PMID:26016521
13	Additive and epistatic genome-wide association for growth and ultrasound scan measures of carcass-related traits in Brahman cattle.	Ali AA, Khatkar MS, Kadarmideen HN, Thomson PC.	J Anim Breed Genet. 2015 Apr;132(2):187-97. doi: 10.1111/jbg.12147. Epub 2015 Mar 6.	J Anim Breed Genet. 2015	PMID:25754883
14	QTLs associated with dry matter intake, metabolic mid-test weight, growth and feed efficiency have little overlap across 4 beef cattle studies.	Saatchi M, Beever JE, Decker JE, Faulkner DB, Freetly HC, Hansen SL, Yampara-Iquise H, Johnson KA, Kachman SD, Kerley MS, Kim J, Loy DD, Marques E, Neibergs HL, Pollak EJ, Schnabel RD, Seabury CM, Shike DW, Snelling WM, Spangler ML, Weaber RL, Garrick DJ, et al.	BMC Genomics. 2014 Nov 20;15:1004. doi: 10.1186/1471-2164-15-1004.	BMC Genomics. 2014	PMID:25410110

15	Whole genome association study identifies regions of the bovine genome and biological pathways involved in carcass trait performance in Holstein-Friesian cattle.	Doran AG, Berry DP, Creevey CJ.	BMC Genomics. 2014 Oct 1;15:837. doi: 10.1186/1471-2164-15-837.	BMC Genomics. 2014	PMID:25273628
16	Validation of genetic polymorphisms on BTA14 associated with carcass trait in a commercial Hanwoo population.	Sharma A, Dang CG, Kim KS, Kim JJ, Lee HK, Kim HC, Yeon SH, Kang HS, Lee SH.	Anim Genet. 2014 Dec;45(6):863-7. doi: 10.1111/age.12204. Epub 2014 Aug 28.	Anim Genet. 2014	PMID:25164077
17	Large-effect pleiotropic or closely linked QTL segregate within and across ten US cattle breeds.	Saatchi M, Schnabel RD, Taylor JF, Garrick DJ.	BMC Genomics. 2014 Jun 6;15:442. doi: 10.1186/1471-2164-15-442.	BMC Genomics. 2014	PMID:24906442
18	Single nucleotide polymorphisms for feed efficiency and performance in crossbred beef cattle.	Abo-Ismail MK, Vander Voort G, Squires JJ, Swanson KC, Mandell IB, Liao X, Stothard P, Moore S, Plastow G, Miller SP.	BMC Genet. 2014 Jan 30;15:14. doi: 10.1186/1471-2156-15-14.	BMC Genet. 2014	PMID:24476087
19	Bivariate genome-wide association analysis of the growth and intake components of feed efficiency.	SerÃ£o NV, GonzÃ¡lez-PeÃ±a D, Beever JE, Bollero GA, Southey BR, Faulkner DB, Rodriguez-Zas SL.	PLoS One. 2013 Oct 29;8(10):e78530. doi: 10.1371/journal.pone.0078530. eCollection 2013.	PLoS One. 2013	PMID:24205251
20	Genome-wide association study identifies major loci for carcass weight on BTA14 in Hanwoo (Korean cattle).	Lee SH, Choi BH, Lim D, Gondro C, Cho YM, Dang CG, Sharma A, Jang GW, Lee KT, Yoon D, Lee HK, Yeon SH, Yang BS, Kang HS, Hong SK.	PLoS One. 2013 Oct 7;8(10):e74677. doi: 10.1371/journal.pone.0074677. eCollection 2013.	PLoS One. 2013	PMID:24116007
21	Genome-wide association analyses for carcass quality in crossbred beef cattle.	Lu D, Sargolzaei M, Kelly M, Vander Voort G, Wang Z, Mandell I, Moore S, Plastow G, Miller SP.	BMC Genet. 2013 Sep 11;14:80. doi: 10.1186/1471-2156-14-80.	BMC Genet. 2013	PMID:24024930

22	Genome-wide association analyses for growth and feed efficiency traits in beef cattle.	Lu D, Miller S, Sargolzaei M, Kelly M, Vander Voort G, Caldwell T, Wang Z, Plastow G, Moore S.	J Anim Sci. 2013 Aug;91(8):3612-33. doi: 10.2527/jas.2012-5716. Epub 2013 Jul 12.	J Anim Sci. 2013	PMID:23851991
23	Bayesian genome-wide association analysis of growth and yearling ultrasound measures of carcass traits in Brangus heifers.	Peters SO, Kizilkaya K, Garrick DJ, Fernando RL, Reecy JM, Weaber RL, Silver GA, Thomas MG.	J Anim Sci. 2012 Oct;90(10):3398-409. doi: 10.2527/jas.2012-4507. Erratum in: J Anim Sci. 2013 Mar;91(3):1522.	J Anim Sci. 2012	PMID:23038745
24	Genome-wide association study identified three major QTL for carcass weight including the PLAG1-CHCHD7 QTN for stature in Japanese Black cattle.	Nishimura S, Watanabe T, Mizoshita K, Tatsuda K, Fujita T, Watanabe N, Sugimoto Y, Takasuga A.	BMC Genet. 2012 May 20;13:40. doi: 10.1186/1471-2156-13-40.	BMC Genet. 2012	PMID:22607022
25	Genetic variation in PLAG1 associates with early life body weight and peripubertal weight and growth in Bos taurus.	Littlejohn M, Grala T, Sanders K, Walker C, Waghorn G, Macdonald K, Coppieters W, Georges M, Spelman R, Hillerton E, Davis S, Snell R.	Anim Genet. 2012 Oct;43(5):591-4. doi: 10.1111/j.1365-2052.2011.02293.x. Epub 2011 Dec 7.	Anim Genet. 2012	PMID:22497486
26	Genetic markers on BTA14 predictive for residual feed intake in beef steers and their effects on carcass and meat quality traits.	Lindholm-Perry AK, Kuehn LA, Snelling WM, Smith TP, Ferrell CL, Jenkins TG, King DA, Shackelford SD, Wheeler TL, Freetly HC.	Anim Genet. 2012 Oct;43(5):599-603. doi: 10.1111/j.1365-2052.2011.02307.x. Epub 2012 Feb 9.	Anim Genet. 2012	PMID:22497335
27	Genome-wide association analysis for feed efficiency in Angus cattle.	Rolf MM, Taylor JF, Schnabel RD, McKay SD, McClure MC, Northcutt SL, Kerley MS, Weaber RL.	Anim Genet. 2012 Aug;43(4):367-74. doi: 10.1111/j.1365-2052.2011.02273.x. Epub 2011 Oct 24.	Anim Genet. 2012	PMID:22497295

28	Evaluation of Bovine chemerin (RARRES2) Gene Variation on Beef Cattle Production Traits.	Lindholm-Perry AK, Kuehn LA, Rempel LA, Smith TP, Cushman RA, McDaneld TG, Wheeler TL, Shackelford SD, King DA, Freetly HC.	Front Genet. 2012 Mar 29;3:39. doi: 10.3389/fgene.2012.00039. eCollection 2012.	Front Genet. 2012	PMID:22479267
29	Whole-genome QTL scan for ultrasound and carcass merit traits in beef cattle using Bayesian shrinkage method.	Nalaila SM, Stothard P, Moore SS, Li C, Wang Z.	J Anim Breed Genet. 2012 Apr;129(2):107-19. doi: 10.1111/j.1439-0388.2011.00954.x. Epub 2011 Sep 8.	J Anim Breed Genet. 2012	PMID:22394233
30	Association, effects and validation of polymorphisms within the NCAPG - LCORL locus located on BTA6 with feed intake, gain, meat and carcass traits in beef cattle.	Lindholm-Perry AK, Sexten AK, Kuehn LA, Smith TP, King DA, Shackelford SD, Wheeler TL, Ferrell CL, Jenkins TG, Snelling WM, Freetly HC.	BMC Genet. 2011 Dec 14;12:103. doi: 10.1186/1471-2156-12-103.	BMC Genet. 2011	PMID:22168586
